# Simple sequence repeat marker development from bacterial artificial chromosome end sequences and expressed sequence tags of flax (*Linum usitatissimum* L.)

**DOI:** 10.1007/s00122-012-1860-4

**Published:** 2012-04-07

**Authors:** Sylvie Cloutier, Evelyn Miranda, Kerry Ward, Natasa Radovanovic, Elsa Reimer, Andrzej Walichnowski, Raju Datla, Gordon Rowland, Scott Duguid, Raja Ragupathy

**Affiliations:** 1 Cereal Research Centre, Agriculture and Agri-Food Canada, 195 Dafoe Road, Winnipeg, MB R3T 2M9 Canada; 2 Department of Plant Science, University of Manitoba, 66 Dafoe Road, Winnipeg, MB R3T 2N2 Canada; 3 Plant Biotechnology Institute, National Research Council, 110 Gymnasium Place, Saskatoon, SK S7N 0W9 Canada; 4 Crop Development Centre, University of Saskatchewan, 51 Campus Drive, Saskatoon, SK S7N 5A8 Canada; 5 Morden Research Station, Agriculture and Agri-Food Canada, 101 Route 100, Unit 100, Morden, MB R6M 1Y5 Canada

## Abstract

Flax is an important oilseed crop in North America and is mostly grown as a fibre crop in Europe. As a self-pollinated diploid with a small estimated genome size of ~370 Mb, flax is well suited for fast progress in genomics. In the last few years, important genetic resources have been developed for this crop. Here, we describe the assessment and comparative analyses of 1,506 putative simple sequence repeats (SSRs) of which, 1,164 were derived from BAC-end sequences (BESs) and 342 from expressed sequence tags (ESTs). The SSRs were assessed on a panel of 16 flax accessions with 673 (58 %) and 145 (42 %) primer pairs being polymorphic in the BESs and ESTs, respectively. With 818 novel polymorphic SSR primer pairs reported in this study, the repertoire of available SSRs in flax has more than doubled from the combined total of 508 of all previous reports. Among nucleotide motifs, trinucleotides were the most abundant irrespective of the class, but dinucleotides were the most polymorphic. SSR length was also positively correlated with polymorphism. Two dinucleotide (AT/TA and AG/GA) and two trinucleotide (AAT/ATA/TAA and GAA/AGA/AAG) motifs and their iterations, different from those reported in many other crops, accounted for more than half of all the SSRs and were also more polymorphic (63.4 %) than the rest of the markers (42.7 %). This improved resource promises to be useful in genetic, quantitative trait loci (QTL) and association mapping as well as for anchoring the physical/genetic map with the whole genome shotgun reference sequence of flax.

**Electronic supplementary material:**

The online version of this article (doi:10.1007/s00122-012-1860-4) contains supplementary material, which is available to authorized users.

## Introduction

Flax (*Linum usitatissimum* L.) has been cultivated for several thousand years mainly for its seed oil and its high-quality stem fibres. In North America, flax is grown primarily as an oilseed crop used for food and feed as well as in bio-product applications such as linoleum flooring, paint and varnishes. Most oilseed flax varieties are rich in omega-3 (alpha linolenic acid, 55–57 %) fatty acid which has been functionally associated with numerous health claims. ‘Solin’ varieties with loss of function mutations in the fatty acid desaturase 3 (*fad3*) genes are low in omega-3 (2–3 %) and high in omega-6 (~70 %) fatty acids, characteristics required for margarine processing. Flax oil extraction generates a meal which is rich in protein and is sought after as animal feed.

Until recently, the stems of North American oilseed flax were considered undesirable because the persistence of straw in the field was problematic. However, in the last decade, the fibre industry has placed substantial effort into the development of high-value products from oilseed flax stems with applications in the pulp, technical fibre and bio-fuel industries. A shift from oilseed flax towards dual purpose or total utilization flax is currently occurring. To assist breeding efforts towards simultaneous improvements of seed and stem traits, breeders need a good grasp of the complexity of the genetic mechanisms underlying traits such as oil content, fatty acid composition, stem fibre content and fibre composition. Knowledge of the existing genetic diversity for these traits in primary and secondary gene pools is also essential to accelerate their introgression in breeding programs. Quantitative trait loci (QTL) and association mapping (AM) studies have the ability to provide some insights into genetic mechanisms of complex traits and provide molecular markers to implement marker-assisted breeding.

To date, only a limited number of useful markers have been developed in flax and, as a consequence, genetic maps and QTL studies remain limited (Cloutier et al. 2011; Oh et al. 2000; Spielmeyer et al. 1998). While some isozyme, RAPD and AFLP markers have been developed in flax (Everaert et al. 2001; Fu 2006; Krulickova et al. 2002; Spielmeyer et al. 1998), such marker systems are either labour-intensive or suffer from low reproducibility. Microsatellites or simple sequence repeats (SSRs) consist of tandemly repeated short motifs of 2–6 nucleotides. SSR markers are based on the amplification size polymorphism generated when lines have variable numbers of these short tandem repeats in a particular locus. The abundance, distribution, reproducibility and generally codominant nature of SSR markers make them highly suitable for linkage mapping and genetic diversity studies (Cloutier et al. 2009; Soto-Cerda et al. 2011a; Wiesner et al. 2001). SSR markers have been developed through SSR-enriched library screening and, more recently, through the more economical mining of EST or genomic sequence data. A total of 508 SSR markers have been reported as follows: 10 (Wiesner et al. 2001), 23 (Roose-Amsaleg et al. 2006), 35 (Deng et al. 2010), 60 (Soto-Cerda et al. 2011a), 38 (Deng et al. 2011), 9 (Kale et al. 2011), 20 (Rachinskaya et al. 2011), 42 (Bickel et al. 2011), 248 (Cloutier et al. 2009) and 23 (Soto-Cerda et al. 2011b). In addition, commercially available inter simple sequence repeat (ISSR) primers from the University of British Columbia (UBC) collection have been used, mostly in genetic diversity studies of *L. usitatissimum* L. or its wild progenitor *L. bienne* Mill. (Chen et al. 1998; Rajwade et al. 2010; Uysal et al. 2010; Wiesnerova and Wiesner 2004).

The ability to detect QTL using genetic maps of a segregating population or by linkage disequilibrium (LD) in AM studies depends on the marker saturation, the distribution and the accuracy of the phenotypic characterization of the traits. Single nucleotide polymorphism (SNP) markers promise to provide the high level of saturation (several thousands) that is paramount to QTL identification by AM in low LD regions of the genome. Developing and applying thousands of SSR markers would be comparatively costly due to the labour involved in their assessment. However, SSRs remain an excellent marker system for the construction of skeletal genetic maps onto which SNPs can be added (Allen et al. 2011). The aim of this project was to increase the number of publicly available SSR markers in flax to over 1,000, on par with other major crops. To realize that goal, we mined additional ESTs, sequenced more than 80,000 flax bacterial artificial chromosome (BAC) ends and mined them for the presence of SSRs. Polymorphism was assessed on a set of 16 flax genotypes and comparative analyses of EST-SSRs and gDNA-SSRs was performed.

## Materials and methods

### Plant materials

A set of 16 flax accessions were grown in a growth cabinet. The leaf and stem tissue of plantlets at the first branching stage were collected and DNA was extracted using a modified CTAB method (Cloutier et al. 2001). The DNA was quantified using a fluorometer and diluted to a 10 ng/μL working solution. The 16 genotypes represent oilseed types with different fatty acid profiles as well as fibre types (Table 1).

**Table 1:** Description of the 16 flax genotypes used to assess polymorphism of the SSR markers

Accession	Country	Type	Fatty acid characteristics	Reference
AC Emerson	Canada	Oilseed	Conventional	Kenaschuk et al. (1996)
CDC Bethune	Canada	Oilseed	Conventional	Rowland et al. (2002)
Lirina	Latvia	Oilseed	Conventional	
Macbeth	Canada	Oilseed	Conventional	Duguid et al. (2003)
Double Low	Unknown	Oilseed		
Prairie Grande	Canada	Oilseed	Conventional	CFIA Application no. 07-5916
SP2047	Canada	Oilseed	Low linolenic	Dribnenki et al. (2003)
UGG5-5	Canada	Oilseed	High linolenic	
Atlas	Sweden	Oilseed		Åkerman et al. (1951)
Bolley Golden	USA	Oilseed		USDA (1931) CN19160
E1747	Canada	Oilseed	Low linolenic	Rowland (1991)
Hermes	France	Fibre		
Linola 989	Canada	Oilseed	Low linolenic	Dribnenki et al. (1996)
Shape	Canada	Oilseed	Conventional	CFIA certificate no. 3840
Tabor	Czech Rep.	Fibre		
Viking	USA	Fibre		USDA (1945)

### SSR design

A BAC library of cultivar CDC Bethune was constructed (Table 2). A total of 43,776 clones were sequenced from both ends by the BC Cancer Agency Genome Sciences Centre (Vancouver, Canada) using universal primers, Sanger’s dideoxy chain termination method with Big Dye V3.1 chemistry and resolved on an ABI 3730xl (Applied Biosystems, Foster City, USA). Trimmed high-quality BAC-end sequences (BESs) totalling ~56 Mb were mined with the Perl script MISA (Thiel et al. 2003) for the presence of putative SSRs using criteria of a minimum of nine repeats for dinucleotide, six repeats for trinucleotide and five repeats for tetra-, penta- and hexa-nucleotide motifs. Primers were designed from BES containing putative SSR motifs as previously described (Cloutier et al. 2009).

**Table 2:** CDC Bethune flax BAC library and BAC-end sequences

Genotype	CDC Bethune		
BAC vector	*p*IndigoBAC-5		
*E. coli* host	DH10B		
Enzymes	*Hin*dIII	*Bam*HI	Total
Number of clones	40,704	51,456	92,160
Average insert size (kb)	150	135	142
Genome coverage^a^	16.5×	18.8×	35.4×
Number of BAC clones sequenced	20,352	23,424	43,776
Number of BESs	40,704	46,848	87,552
Number of failed sequences	1,313	2,807	4,120
Number of short sequences (<100 bp)	890	923	1,813
Number of high-quality sequences	38,501	43,118	81,619
Average sequence length (bp) of high-quality BESs	674	694	684
Total sequence length (bp)	25,979,571	29,944,023	55,923,594

^a^Based on estimated genome size for CDC Bethune of 370 Mb (Ragupathy et al. 2011)

A total of 243,272 flax Expressed Sequence Tags (ESTs) from flax generated by the NAPGEN consortium, the TUFGEN project (Venglat et al. 2011) or publicly available in GenBank, were assembled into 34,156 unigenes comprising 14,374 contigs and 19,782 singletons using criteria previously described (Cloutier et al. 2009). A total of 33,163 unigenes of high quality and sufficient length were mined with the same criteria as described above for the BES. Putative SSRs previously detected and assessed from an original set of 146,611 assembled ESTs (Cloutier et al. 2009) were ignored and only the putative SSRs from novel contigs and/or singletons were retained for primer design performed using Primer3 (Rozen and Skaletsky 2000).

### Polymorphism assessment

Each primer pair was assessed using DNA from the 16-genotype panel as previously described (Cloutier et al. 2009). Most amplicons were resolved using the GeneScan 500 ROX size standard (ABI) but amplicons larger than 450 bp were resolved using MapMarker 1000 (BioVentures Inc, Murfreesboro, USA) which gave improved sizing accuracy for larger fragments. Allele sizes were recorded for each genotype of the panel. Primer pairs were considered polymorphic if at least one of the 16 genotypes had a different allele size; monomorphic when all lines amplified the same size fragment; and failed when no consistent PCR product was observed after two additional PCR attempts at 58 and 49 °C, respectively. Primer pairs that amplified more than one polymorphic locus were scored independently. The polymorphic information content (PIC) value was estimated for each marker to determine their potential usefulness in determining the genetic variability of other *Linum* accessions (Botstein et al. 1980). To illustrate the genetic relationship of the 16 flax accessions, we constructed a dendrogram using the neighbour-joining method (Nei 1973) as implemented in PowerMarker (Liu and Muse 2005).

## Results

A total of 1,660 BESs were identified to have at least one putative SSR, from which 1,164 primer pairs were designed. A total of 673 (57.8 %) primer pairs were polymorphic and detected 720 loci with 43 primer pairs detecting two polymorphic loci and two primer pairs detecting three polymorphic loci (Supplementary Table S1). The monomorphic BES-SSR markers totalled 478 (41.1 %) and only 13 (1.1 %) failed (did not work, DNW).

A total of 382 putative novel EST-SSRs were identified from the EST assembly and 342 primer pairs were designed. Roughly the same proportion of EST-SSR primer pairs was polymorphic (145; 42.4 %; Supplementary Table S2) and monomorphic (153; 44.7 %) while 44 failed (12.9 %). The 145 polymorphic EST-SSRs detected 149 loci.

The number of alleles detected at a single polymorphic locus ranged from 2 to 9 with an average of 2.76 in the BES-SSRs and ranged from 2 to 6 with an average of 2.36 in the EST-SSRs (Supplementary Tables S1 and S2). The mean polymorphism information content (PIC) value was 0.39 (0.12–0.85) and 0.34 (0.12–0.70) for the BES-SSRs and EST-SSRs, respectively. Frequency distribution of PIC values of SSR loci showed that nearly 25 % of the markers had PIC values greater than 0.5 (Supplementary Figure S1).

**Fig. 1:**
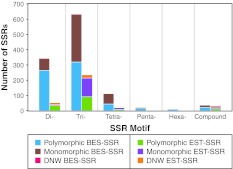
Number of SSRs classified based on motif type, source (*BES* or *EST*) and polymorphism. *DNW* (did not work) represents primer pairs that failed to amplify reproducible amplicons

The total number of SSR markers and their associated polymorphism for each motif length is illustrated in Fig. 1. Regardless of the source of SSRs trinucleotide SSRs were the most abundant representing 54.6 and 68.7 % of the BES-SSRs and EST-SSRs, respectively. These trinucleotide motifs also displayed a higher proportion of monomorphic amplicons regardless of the source. Dinucleotide motifs were only 30.6 and 16.8 % of the two SSR categories but they represented 40.6 and 24.8 % of the total polymorphic SSRs. Compound SSRs represented sequences that had two SSR motifs within 100 bp. These motifs were generally different from one another and compound SSRs only represented 3.1 and 9.6 % of all BES-SSRs and EST-SSRs, respectively. In total, 77 and 63 % of the dinucleotide, 50 and 39 % of the trinucleotide and 39 and 43 % of the tetranucleotide motif SSRs were polymorphic in the BES and EST datasets, respectively.

**Fig. 2:**
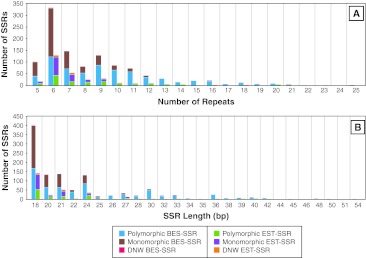
Number of SSRs and associated polymorphism based on **a** the number of repeats and **b** the SSR length (bp). SSRs are also classified as per their source (*BES* or *EST*). *DNW* (did not work) represents primer pairs that failed to amplify reproducible amplicons

BES-SSRs tended to have a higher number of repeats per locus with 41.1 % having 9 or more repeats as compared to 26.9 % for EST-SSRs (Fig. 2a). SSRs with higher numbers of repeats tended to be more polymorphic. 41.1 % of the BES-SSRs with 9–25 repeats accounted for 56.1 % of the polymorphic BES-SSRs while 26.9 % of the EST-SSRs with nine or more repeats accounted for 38.3 % of the polymorphic EST-SSRs. SSR length is a measurement of the motif length and the number of repeats. Long SSRs (25–54 bp) represented a higher proportion of the total SSRs detected in BES (24.1 %) as compared to ESTs (15.9 %) and were more polymorphic than shorter SSR loci (Fig. 2b).

**Fig. 3:**
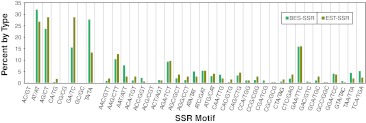
Percentage of dinucleotide and trinucleotide SSRs classified based on their source (*BES* or *EST*) and their motif

Of the eight possible dinucleotide motifs, four, namely AT/AT, AG/CT, GA/TC and TA/TA, accounted for 99.4 and 98 % of the dinucleotide motifs from BES-SSRs and EST-SSRs, respectively (Fig. 3). The motifs AC/GT and GA/TC were represented by only four SSRs in total in both datasets and CG/CG and GC/GC were completely absent. Approximately twice as many GA/TC motifs were observed in EST-SSRs (28.8 %) as compared to BES-SSRs (15.6 %) while the opposite trend was true for the TA/TA motif. Of the 30 possible trinucleotide motifs, AAG/CTT, AGA/TCT and GAA/TTC were most frequent representing 36 and 38.7 % of the BES- and EST-SSRs, respectively (Fig. 3). Some motifs were represented by one or two SSRs only and motif ACG/CGT was not detected at all in either type of sequence. The proportion of SSRs originating from each motif was similar between the two datasets with a few exceptions such as AAT/ATT, TAA/TTA and TCA/TGA that were proportionately higher in BES-SSRs as compared to EST-SSRs. Frequency distributions for tetra-, penta- and hexa-nucleotide motif SSRs are more difficult to discern because they represent only a small proportion of the total SSRs detected. Nevertheless, there seem to be some biases, i.e., some motifs were present more frequently than expected by random distribution such as AAAG/CTTT and ATTA/TAAT which appeared ten and eight times in the BES-SSRs, respectively.

**Fig. 4:**
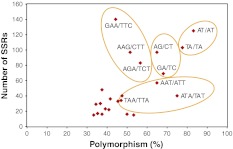
Diagram illustrating that two dinucleotide and two trinucleotide motifs and their respective iterations represent the majority of the SSRs in flax and are also the most polymorphic

Polymorphism rates varied greatly among motifs. However, this type of data is only valid for high-frequency motifs because percent polymorphism associated with a rare motif does not provide an accurate estimate. Considering only motifs represented by at least 15 SSRs, a scatter plot of the total number of SSRs by motif against the percentage of polymorphic SSR per motif for 23 different motifs (including 4 dinucleotide and 19 trinucleotide motifs) showed that 2 dinucleotide (AT/TA and AG/GA) and 2 trinucleotide (AAT/ATA/TAA and GAA/AGA/AAG) motifs and their iterations contributed the majority (536) of the polymorphic SSRs (Fig. 4).

Using all 869 SSR markers, a dendrogram of the 16 accessions was constructed (Supplementary Figure S2). Fibre and oilseed flax belonged to separate clades. Also, within the oilseed group, the low linolenic accessions clustered together.

## Discussion

Here, we described the development and analysis of 818 novel polymorphic SSR primer pairs in flax detecting 869 loci, of which 145 primer pairs were derived from ESTs and 673 from BESs, more than doubling the combined previously available flax SSR collections of 508 (Table 3). With a total of 1,326 SSR markers now publicly available, flax compares favourably to other major crops (Varshney et al. 2006b, 2010). Taken together, these resources should prove valuable in genetic, QTL and association mapping, for anchoring the physical map and integration of the whole genome shotgun sequence assembly.

**Table 3:** Simple sequence repeats (SSRs) currently available for flax including reference, source, number of genotypes tested and SSR statistics

Reference	SSR source^a^	Genotypes tested	Polymorphic primer pairs	Loci detected	Loci per primer pair	Alleles per locus^b^	PIC^b,c^
Wiesner et al. (2001)	Genomic	8	10	–	–	3.7 (2–8)	0.60 (0.25–1.00)
Roose-Amsaleg et al. (2006)	Genomic	93	23	28	1.22	3.3 (2–10)	0.33 (0.02–0.73)
Cloutier et al. (2009)	ESTs	23	248	275	1.11	2.3 (2–7)	0.35 (0.08–0.82)
Soto-Cerda et al. (2011a)	Genomic	60	60	66	1.10	3.0 (2–8)	0.39 (0.06–0.87)
Deng et al. (2010)	Genomic	8	35	37	1.06	3.5 (2–6)	0.60 (0.23–0.84)
Soto-Cerda et al. (2011b)	ESTs	61	23	23	1.00	2.3 (2–4)	0.38 (0.08–0.55)
Deng et al. (2011)	Genomic	8	38	38	1.00	3.4 (2–12)	0.43 (0.20–0.88)
Kale et al. (2011)	Genomic	27	9	–	–	–	–
Bickel et al. (2011)	Genomic	19	42	42	1.00	3.3 (2–8)	0.47 (0.10–0.86)
Rachinskaya et al. (2011)	Genomic	15	20	22	1.10	3.0 (2–7)	0.42 (0.03–0.77)
Cloutier et al. (this publication)	ESTs	16	145	149	1.03	2.4 (2–6)	0.34 (0.12–0.70)
Cloutier et al. (this publication)	BESs	16	673	720	1.07	2.8 (2–9)	0.39 (0.12–0.85)
Total			1,326	1,400	1.07		

^a^Genomic library or published genomic sequences; expressed sequence tags (ESTs); BAC-end sequences (BESs)

^b^Average value followed by range in brackets

^c^Polymorphism information content

Traditionally, SSRs were developed from SSR-enriched libraries which represented a major bottleneck (Kalia et al. 2011). However, with technological advances in generating large-scale sequence data (EST, exome, genomic surveys, BES, whole genome sequence, etc.) and their availability in public domain, in silico approaches to the identification of putative SSRs have become practical (Tang et al. 2008). Here, we capitalized on the availability of ESTs (Cloutier et al. 2009; Venglat et al. 2011) and BESs (Ragupathy et al. 2011) to develop the largest collection of flax SSRs to date.

The number of SSRs assessed herein is sufficiently large to provide general conclusions regarding source (EST vs. genomic DNA), motif type, length, sequence and evolution of SSR loci. Overall, no major difference existed between EST-SSRs and BES-SSRs with the exception of the failure rate that was higher in EST-SSRs, probably as a result of poor primer binding due to their design over a splice site, mismatches caused by poor sequence quality or the presence of a large intron hindering amplification (Tang et al. 2008).

Trinucleotide SSRs were the most abundant in *Arabidopsis* (Mun et al. 2006; Tian et al. 2004), *Medicago* (Mun et al. 2006), soybean (Hisano et al. 2007; Mun et al. 2006; Tian et al. 2004), rice (Mun et al. 2006), pea (Gong et al. 2010), sugarcane (Cordeiro et al. 2001; Parida et al. 2009a), chickpea (Choudhary et al. 2009), wheat (Peng and Lapitan 2005; Yu et al. 2004), barley (Thiel et al. 2003; Varshney et al. 2006a), pepper (Yi et al. 2006), *Lotus japonicus* (Mun et al. 2006) and citrus (Chen et al. 2006). These estimates could be construed as biased because in the majority of the above studies, SSRs were mined from ESTs which are known to display a prevalence of trinucleotides (Cavagnaro et al. 2010; Li et al. 2004; Morgante et al. 2002; Tian et al. 2004). Of the 12 published plant genomes analyzed by Ragupathy et al. (2011), only *Brachypodium* had a higher percentage of trinucleotide SSRs. In flax, trinucleotide SSRs were also the most abundant but dinucleotide SSRs were the most polymorphic, as reported for several other crops (Blair et al. 2009; Cavagnaro et al. 2010; Hisano et al. 2007; Mun et al. 2006). The above SSR motif results are not always consistent and readily comparable because the thresholds of parameters used for identification of the SSR loci, especially the number of repeats per motif, are not uniform across species and even across research reports of the same species. Mononucleotide repeats, often not accounted for, were reported to be the most abundant in *Brachypodium*, rice, sorghum, *Arabidopsis*, *Medicago* and *Populus* (Cardle et al. 2000; Gupta and Prasad 2009; Mun et al. 2006; Sonah et al. 2011).

In flax, trinucleotides were more numerous in ESTs (68.7 %) compared to BESs (54.6 %) likely because of the suppression of non-trimeric SSRs in coding regions which could result in changes in reading frames (Kalia et al. 2011). Polymorphism was also positively correlated with the number of repeats per locus and the overall locus length. Among sources, BES-SSRs (58 %, average PIC 0.39) displayed a significantly higher level of polymorphism than EST-SSRs (42 %, average PIC 0.34) as previously reported (Eujayl et al. 2002; Kalia et al. 2011). Even with only ~40 % of EST-SSRs being polymorphic, flax has a higher polymorphism than wheat, barley, soybean and cotton (Eujayl et al. 2002; Han et al. 2004; Hisano et al. 2007; Thiel et al. 2003; Varshney et al. 2006a). However, the polymorphism level can vary significantly across studies because it is also a reflection of the number of lines surveyed in the panel and its genetic diversity. Here, we used 16 flax oilseed and fibre accessions providing a good genetic diversity of the breeding material but not necessarily of *Linum usitatissimum* because all accessions investigated were varieties or advanced breeding lines.

A distinct bias towards certain motifs was evident: two dinucleotide and two trinucleotide motifs and their iterations accounted for 65.5 % (536/818) of all polymorphic motifs (Fig. 4). Of the 1,506 target sequences from which primers were designed, these motifs represented more than half (845) and their polymorphism level greatly exceeded that of all the other motifs taken together (63.4 vs. 42.7 %). At the opposite end of the spectrum, motif ACG/CGT and its iterations (CGA/TCG and GAC/GTC) represented only 14 of the 1,506 SSRs (<1 %) with only four being polymorphic while dinucleotide CG/GC was not detected in either flax ESTs or BESs (Supplementary Table 2). The biases observed were both in relative abundance and polymorphism level. Motif abundance seems to be species specific due to factors such as genome content and composition, variation in rate of mutation across genome including rate of slippage and codon usage (Buschiazzo and Gemmell 2006; Sonah et al. 2011). The trinucleotide motif AGC/GCA/CAG was the most abundant trinucleotide motif in 8 crops and AGG/GGA/GAG in 4 while AG/GA was the most abundant dinucleotide in 14 crops and AC/CA in 4 (Yu et al. 2009). In flax, GAA/AAG/AGA with 320 and AT/TA with 228 were the most abundant trinucleotide and dinucleotide motifs, respectively, indicative of its unique SSR genome composition as compared to other crops.

Repeat numbers of EST-SSRs tended to be lower than gDNA-SSRs (Morgante et al. 2002). This was particularly true in flax where 5–7 repeat SSRs represented 64.7 % of all EST-SSRs but only 51.6 % of the BES-SSRs. These short SSRs were less polymorphic than the longer ones regardless of the source as previously reported (Wierdl et al. 1997; Ellegren 2004; Cavagnaro et al. 2010; Blair et al. 2009) and somewhat in disagreement with Tang et al. (2008). However, in this latter case, they considered SSRs with as few as 4 or 5 repeats even for dinucleotides which was not the case in this study because the SSR identification was performed using ESTs and BES from a single genotype (CDC Bethune) while Tang et al. (2008) used multiple genotypes. Surprisingly, these short SSRs were more polymorphic than long SSRs, possibly because in this case, they were derived exclusively from ESTs where long SSRs can be deleterious (Sureshkumar et al. 2009; Tang et al. 2008).

Predominant distribution of long alleles of SSR loci in genomic regions containing both coding [~26.8 % in flax, Ragupathy et al. (2011)] and non-coding sequences compared to EST-SSRs in this study could be associated with factors contributing to SSR origin and evolution per se. For instance, a dinucleotide motif arrayed 3 or 4 times in a locus may originate from cryptically simple sequences by both substitutions and indel mutations (Buschiazzo and Gemmell 2006). These substrate sequences further expand to an array of repeating units in a given locus, mainly through two mechanisms: slippage during DNA replication in a repeat domain (stabilized with an inefficient DNA mismatch repair-MMR system of the host) and unequal crossing over (Ellegren 2004). Studies of SSR evolution in the human genome suggested a mutational bias leading to an increase in SSR length at an individual locus (expansion) rather than a decrease in repeat number (contraction) on the evolutionary timescale (Ellegren 2004). Also, neutrality of mutations in the SSR loci present in non-coding regions of the genome favour an increase in repeat length, however, in coding sequences selective constraints against frame shift mutations weed out expansion or contraction of motifs except for triplets (Li et al. 2004). Although repeats containing proteins are well characterized (Faux et al. 2007), expansion of amino acid homopolymer domains beyond a threshold length impacts protein functionality (Kashi and King 2006). Also, conserved regulatory roles associated with some genic SSR sites favoured by selective forces curtail expansion in EST-SSRs despite the potential adaptive advantage (Li et al. 2004; Parida et al. 2009b).

The SSR markers described herein promise to be useful to characterize the genetic variability of other *Linum* accessions. The number of alleles and their relative frequencies are both indicators of a marker’s usefulness (Shete et al. 2000) and are taken into account in the PIC value estimates. More than 200 of the markers described have PIC values greater than 0.5 (Supplementary Figure S1) which should make them particularly useful in characterizing *Linum* collections as illustrated by the relationship observed in the dendrogram of the 16 accessions (Supplementary Figure S2). Indeed, accessions of similar lineage clustered together as predicted by the type (fibre vs. oilseed) and end-use quality (conventional vs. low or high linolenic acid content).

## Conclusion

Here, we described the development of the largest collection of SSRs in flax to date bringing the overall number to over 1,300, comparable to many other major crops. A comprehensive comparative analysis of the composition and polymorphism of SSRs developed from ESTs and BES was performed showing some important differences between flax and other crops. The SSR resource described herein will be useful in genetic, QTL and association mapping. Map-based cloning, physical anchoring of the WGS reference genome and other downstream applications in breeding such as marker-assisted selection are likely to benefit from this important resource, paving the way for genetic improvement of flax.

## Electronic supplementary material

Below is the link to the electronic supplementary material.

Supplementary material 1 (PDF 14.5 kb)

Supplementary material 2 (PDF 6.90 kb)

Supplementary material 3 (PDF 194 kb)

Supplementary material 4 (PDF 246 kb)
