# Supplementary material for: Simple sequence repeat marker development from bacterial artificial chromosome end sequences and expressed sequence tags of flax (Linum usitatissimum L.)
Source: Theor Appl Genet. 2012 Apr 7;125(4):685–94. doi: 10.1007/s00122-012-1860-4 (PMC3405236; doi:10.1007/s00122-012-1860-4)
Supplement: Supplementary file 1 — Supplementary material 1 (PDF 14.5 kb) [file 122_2012_1860_MOESM1_ESM.pdf]

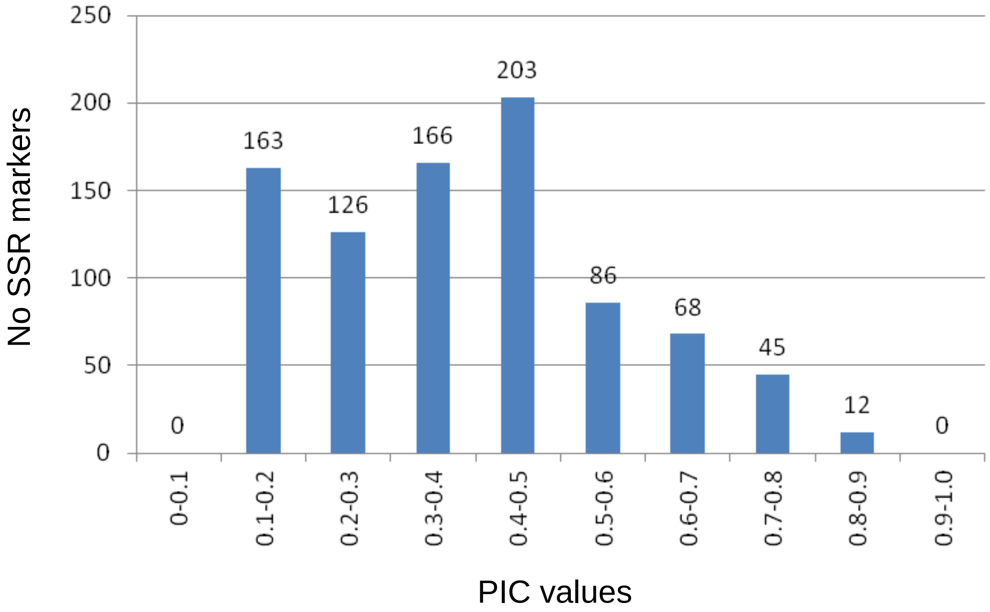

Supplementary Data Figure S1: Frequency distribution of the PIC values of the 869 SSR markers. A total of 211 SSR markers have PIC values greater than 0.5.
