# Supplementary material for: Simple sequence repeat marker development from bacterial artificial chromosome end sequences and expressed sequence tags of flax (Linum usitatissimum L.)
Source: Theor Appl Genet. 2012 Apr 7;125(4):685–94. doi: 10.1007/s00122-012-1860-4 (PMC3405236; doi:10.1007/s00122-012-1860-4)
Supplement: Supplementary file 2 — Supplementary material 2 (PDF 6.90 kb) [file 122_2012_1860_MOESM2_ESM.pdf]

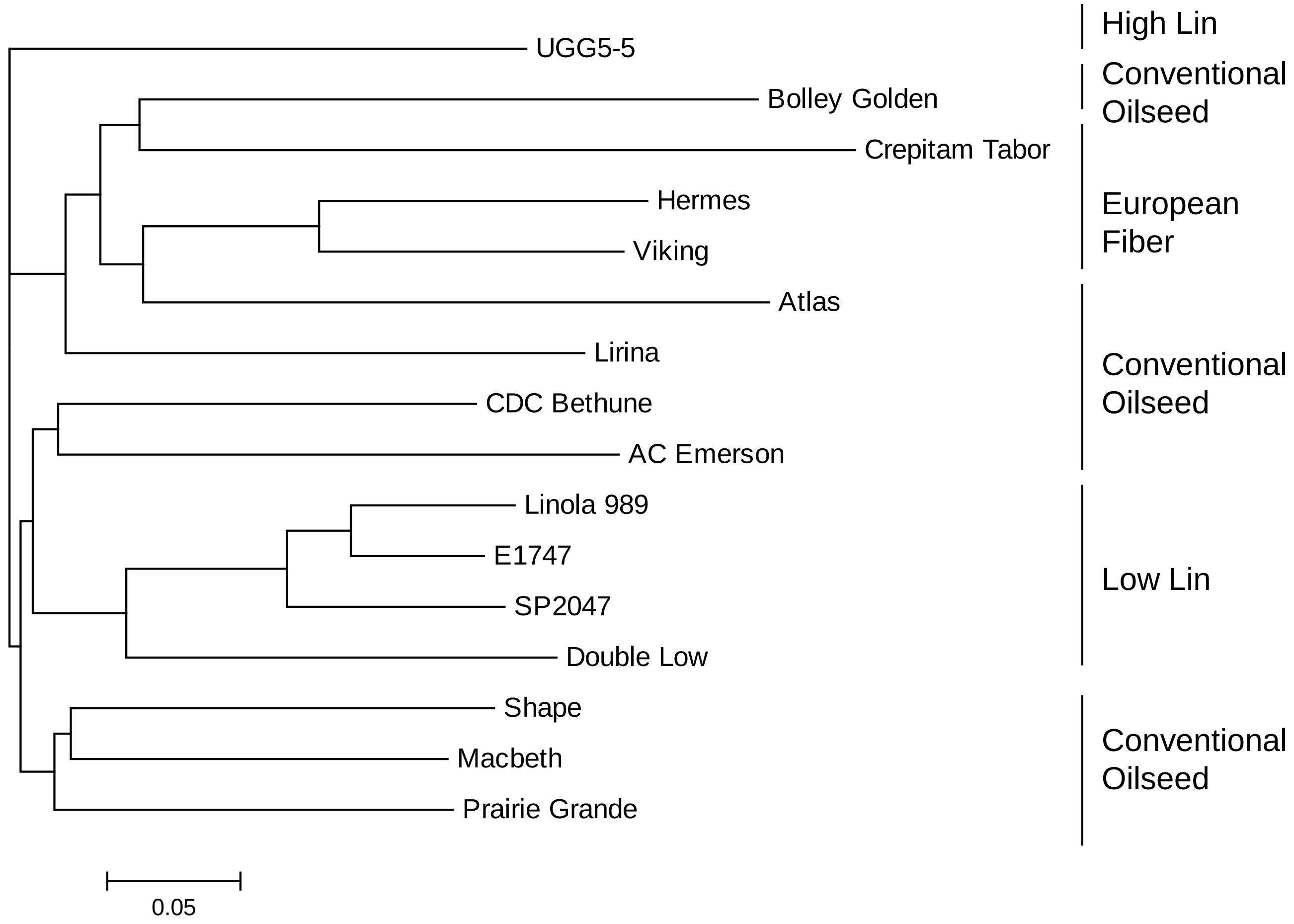

Supplementary data Figure S2: Phylogenetic tree of the 16 accessions generated by the Neighbor-Joining method (Nei 1973) as implemented in PowerMarker (Liu and Muse 2005) using 869 SSR markers derived from the 818 primer pairs described herein.
