# Supplementary material for: Simple sequence repeat marker development from bacterial artificial chromosome end sequences and expressed sequence tags of flax (Linum usitatissimum L.)
Source: Theor Appl Genet. 2012 Apr 7;125(4):685–94. doi: 10.1007/s00122-012-1860-4 (PMC3405236; doi:10.1007/s00122-012-1860-4)
Supplement: Supplementary file 3 — Supplementary material 3 (PDF 194 kb) [file 122_2012_1860_MOESM3_ESM.pdf]

Supplementary Data Table S1: BES-SSR and EST-SSR list including marker name, number of loci, SSR type, SSR sequence, number of alleles, observed allele sizes and primer sequences

BES-SSRs

Marker	No loci	SSR type	SSR sequence	No alleles	observed allele sizes	Primer	Sequence (5' -> 3')	Tm (˚C)	GC content (%)	Length (bp)
Lu2098	1	p3	(AAG)11	2	209 227	Lu2098F	CGGCTGCTGAGAGTATTGG	59.6	57.9	19+20
						Lu2098R	CATCAGAAGCTCGGAGAAGG	60.1	55	20
Lu2100	1	p3	(ATC)9	2	226 229	Lu2100F	AGCGCTGATTGAGCGAGAG	61.9	57.9	19+19
						Lu2100R	GCCATCTGTGGAGAGGAGAG	59.9	60	20
Lu2101	1	p3	(GAA)8	2	289 321	Lu2101F	AAACCACCCCTTCCATATCC	59.9	50	19+20
						Lu2101R	AACCAGCAATCTGACAATCG	58.7	45	20
Lu2102	1	p5	(GATTA)5	2	241 246	Lu2102F	GAGGAATGTCGTGAGTTACAAAC	57.8	43.5	19+23
						Lu2102R	TGCCGAGTGAGTTTGAGTTG	60	50	20
Lu2103	1	p3	(GGA)8	2	233 245	Lu2103F	GCAGTCGTTGCACTTCATGT	59.9	50	19+20
						Lu2103R	AGTGGTGAAGGAGCAGCAAG	60.6	55	20
Lu2105	1	p2	(TA)16	5	212 222 226 232 240	Lu2105F	TGAGTCTATGCAACAATTTATGATTC	58.7	30.8	19+26
						Lu2105R	GACTCGGTGGATATTTGAAGG	58.5	47.6	21
Lu2106	1	p2	(TC)16	2	267 269	Lu2106F	GGAGGAACAGGAAAGGAAGC	60.2	55	19+20
						Lu2106R	CTGCCAAATTTCCTCAGACG	60.8	50	20
Lu2109	1	p2	(AG)11	3	329 337 339	Lu2109F	TGCCCTTCCCATAATACAGC	59.9	50	19+20
						Lu2109R	GGTGGGGAAAATGGGAATAG	60.4	50	20
Lu2110a	2	p3	(CTT)7	2	226 223	Lu2110F	CCTTGCGAACTCTTTTACGC	60	50	19+20
Lu2110b				2	209 212	Lu2110R	AGGAAATGCGAGAATGTTGG	60.1	45	20
Lu2111a	2	p3	(ATC)6	2	306 312	Lu2111F	TCTGCAGTACCCTCGATTCC	60.2	55	19+20
Lu2111b				2	285 291	Lu2111R	AGATTCACCATCAGACGTTGC	60.1	47.6	21
Lu2112	1	p3	(CAT)10	2	231 254	Lu2112F	AGGTTCAGTGCACCACAGG	59.7	57.9	19+19
						Lu2112R	ATGCATGAATGGACAAATGG	59.2	40	20
Lu2113	1	p2	(CT)10	3	342 344 350	Lu2113F	CGGGAAGAAAACATCTCTCG	59.8	50	19+20
						Lu2113R	AAGAGGAACACGAGGACACG	60.3	55	20
Lu2115	1	c	(TAT)8tgttactgctactacttttcagtttttaattaa tcggatttgttcttcttac(TA)15	5	452 465 467 469 471	Lu2115F	TGTTAAGCGTCGTGTGCTC	58.6	52.6	19+19
						Lu2115R	CCCACAAAAAGTGACGAGAG	58.3	50	20
Lu2118	1	p3	(TAA)12	2	327 342	Lu2118F	TACCGGAAGCTACCCATCAC	60	55	19+20
						Lu2118R	CGGGCCCTATTATTTTCTCC	59.8	50	20
Lu2119	1	p2	(GA)12	2	216 218	Lu2119F	TCTGCACAGGGGAAAAGAAG	60.4	50	19+20
						Lu2119R	TAACTTTCCTCCGGGATTTG	59	45	20
Lu2120a	2	p2	(CT)14	2	272 274	Lu2120F	TTCGATAGGCCCCCTTTTAGC	60.5	50	19+20
Lu2120b				2	253 255	Lu2120R	CGCGGTTTTATGATCACTTG	59.2	45	20
Lu2121a	2	p3	(GGA)6	2	null 242	Lu2121F	AAAGCAATGGGCAGAGAATG	60.2	45	19+20
Lu2121b				2	null 238	Lu2121R	TGGGATGTGGAAAGAAGAGC	60.2	50	20
Lu2123	1	p2	(CA)9	2	363 387	Lu2123F	TGCAATAAAAACCCCCAGTC	59.8	45	19+20

											Lu2123R	TGTTTTATGAGCCGACGATG	59.7	45	20
Lu2124a	2	p6	(CAAAAT)5	2	266	272					Lu2124F	CTTTCCTGAATCCCTGCAAC	59.7	50	19+20
Lu2124b				2	249	255					Lu2124R	CCGACAGCCTTATCTCTTGG	59.8	55	20
Lu2125	1	p3	(CAG)6	2	null	210					Lu2125F	AGGGGAGGCAGAGAGAGAAG	60.1	60	19+20
											Lu2125R	GGCTAGAATTCAGGCAGTGG	59.8	55	20
Lu2127a	2	c	(CT)11(AT)9	6	416	418	428	431	435	437	Lu2127F	TCAGGGAGAGATCAGGAACG	60.3	55	19+20
Lu2127b				2	389	391					Lu2127R	AAGGAATCCTAGAGCCAGATGAC	60.1	47.8	23
Lu2128	1	p3	(GAA)11	2	224	233					Lu2128F	TGCCGAGGAAGTCTTTAAGC	59.6	50	19+20
											Lu2128R	TTGCCGTATCTTCACTGCTG	60	50	20
Lu2130	1	p6	(TCATTA)6	3	362	365	368				Lu2130F	AGAGTTGATCCCCTCCTTCG	60.6	55	19+20
											Lu2130R	AGACAAATGGCTGAGCTTGG	60.4	50	20
Lu2131	1	p2	(AT)13	4	329	331	333	335			Lu2131F	TCGTGCCATAACTGAACTGC	59.9	50	19+20
											Lu2131R	TAAGGGGGATTGTCCTTCG	59.9	52.6	19
Lu2132	1	p2	(GA)12	2	284	292					Lu2132F	GAGGCAGAATCCAGTTGAGG	59.8	55	19+20
											Lu2132R	TGCTCTGCGTTCCTCTATCC	60.5	55	20
Lu2135	1	p3	(TAG)6	2	337	388					Lu2135F	GATCATCGTCCAGCAGCAG	60.5	57.9	19+19
											Lu2135R	CACACATGCTTTGCGAGAAC	60.5	50	20
Lu2137	1	p3	(AAG)12	2	363	378					Lu2137F	ATTGATTGGCGGAAGAACTG	60.1	45	19+20
											Lu2137R	TCAGCCATTAGGGTTTCTGG	60.1	50	20
Lu2139	1	p2	(TA)11	3	280	284	286				Lu2139F	ATGTGAACCCGGATTTCAAG	59.8	45	19+20
											Lu2139R	TCACTGCAGTACAGCACACC	58.4	55	20
Lu2141	1	p2	(AG)11	2	369	375					Lu2141F	TCAAAACTTGCAACGTCCAG	59.9	45	19+20
											Lu2141R	ATGTGGTTGAGCTGGGTTG	59.5	52.6	19
Lu2144	1	p3	(ATT)7	2	244	250					Lu2144F	ACTAATCCGCCGCCACTAC	60.1	57.9	19+19
											Lu2144R	TGGTCTAAGAGTTGACCAAACC	58.2	45.5	22
Lu2145	1	p3	(GAA)12	3	283	286	292				Lu2145F	AGAGGGAAAAAGGCTCATCC	59.7	50	19+20
											Lu2145R	GTTCCCACTTCCCATTTTCC	60.5	50	20
Lu2148	1	p2	(TC)14	3	227	229	231				Lu2148F	TGCCAATCGATGATGTTCTC	59.6	45	19+20
											Lu2148R	CTCCAACTTGGTCCAGAAGC	59.8	55	20
Lu2149	1	p2	(AT)11	6	273	275	277	281	287	289	Lu2149F	CTGGAACTGCTTATGCTTGC	58.7	50	19+20
											Lu2149R	AGAGGGGTTTCTTCCCTACG	59.6	55	20
Lu2154a	2	p3	(CAA)8	3	242	248	251				Lu2154F	TTGCGCGTACAGAACAGAAG	60.2	50	19+20
Lu2154b				4	null	226	232	235			Lu2154R	GCGAGATTTCTGGGAGTGAG	60	55	20
Lu2155	1	p2	(CT)13	3	278	280	282				Lu2155F	TTTTCCTCACCCTTGTTTCC	59	45	19+20
											Lu2155R	CCCCCTAGGGAAGATTATGC	59.8	55	20
Lu2156	1	p4	(GGAA)6	2	312	332					Lu2156F	GGAGCAAGCAGAAGAGTTGC	60.3	55	19+20
											Lu2156R	TCAGAGAACATGGTGCAAGC	60	50	20
Lu2157	1	p2	(TA)16	6	211	223	225	227	229	231	Lu2157F	CTACGCGGATTGAGAAGTCC	59.8	55	19+20
											Lu2157R	CCTGCAAGGCTTTGATTAGG	59.8	50	20
Lu2161	1	p2	(AG)25	6	189	195	197	199	201	209	Lu2161F	GGGGAGCTATTAGGACTTCTCC	59.6	54.5	19+22
											Lu2161R	CCCCCTCTTCATCTCTCTCC	60.1	60	20
Lu2162	1	p2	(AT)15	5	242	252	258	262	264		Lu2162F	AATCCCAAATTCCCAACTCC	60	45	19+20

						Lu2162R	GGTCTTTCTTTGTCGCATTCC	61	47.6	21
Lu2163	1	p2	(AT)18	6	378 384 386 388 392 406	Lu2163F	TGGTCTCCTAATTGGCTTGG	60.1	50	19+20
						Lu2163R	CAGGCGGGAGTATGATAGTTG	59.6	52.4	21
Lu2164	1	p5	(ATATA)5	2	372 377	Lu2164F	GCATGATCGTTACTTTAGGATGC	60	43.5	19+23
						Lu2164R	AATGACGCCATCTTTTGTCC	59.9	45	20
Lu2166	1	p3	(GAT)7	2	227 233	Lu2166F	GGTTTGGATGGAAGGAAAGG	60.7	50	19+20
						Lu2166R	TGTTCTCCCCAAACATCGTC	60.9	50	20
Lu2168	1	p2	(TA)9	2	null 295	Lu2168F	ACAAGCAAAAGGGTCACTCG	60.3	50	19+20
						Lu2168R	TGATGGAGCCAGTCATGAAG	59.8	50	20
Lu2169	1	p4	(AAGA)8	2	208 212	Lu2169F	GGATTTGTTTTTGCCTCTCG	59.7	45	19+20
						Lu2169R	AAAGCAAAGCAAAGCAGAGC	59.9	45	20
Lu2170	1	p2	(AT)11	2	321 323	Lu2170F	CTCCTCGATCACCTCTTTCG	59.9	55	19+20
						Lu2170R	GAAGATCAAGGGCTGTGACG	60.8	55	20
Lu2172	1	p2	(AT)10	2	390 392	Lu2172F	AGCTACCCCAACTTGTTTGC	59.2	50	19+20
						Lu2172R	GGCTGGCTTCTTCTTTTGC	60.1	52.6	19
Lu2176	1	p3	(ATA)9	2	271 274	Lu2176F	ATACAGGCCGGTCATTCATC	59.8	50	19+20
						Lu2176R	CCCCTCGTGATTTCCAAAC	60.3	52.6	19
Lu2178	1	p3	(AAT)8	3	292 295 308	Lu2178F	CAGAGGGAAGAAACGAAGAAAG	59.5	45.5	19+22
						Lu2178R	GTGGGAAAACGAAAAGTTGG	59.4	45	20
Lu2182	1	p2	(TA)9	2	336 338	Lu2182F	CTTGCTTCAATCGATCTCTGG	60	47.6	19+21
						Lu2182R	ATTAGGGGTGACACGAGAGC	59.2	55	20
Lu2183a	3	p2	(AT)15	6	386 388 390 392 394 402	Lu2183F	CTTCATGCAGTCCGTTTTTACA	60.2	40.9	19+22
Lu2183b				6	438 440 442 446 448 452	Lu2183R	CAGTTCGTAGTTTACTTGGTGCAG	60.3	45.8	24
Lu2183c				6	456 460 462 466 468 470					
Lu2184a	2	p3	(ATT)10	3	216 222 234	Lu2184F	TGGCCTATATATGACGGTTGC	59.8	47.6	19+21
Lu2184b				3	202 208 220	Lu2184R	AGGAGAGTCCAGGTTCATGC	59.3	55	20
Lu2188	1	p2	(TA)9	2	373 375	Lu2188F	TGGAGCTAAATAAGGAGAGTGTG	57.7	43.5	19+23
						Lu2188R	TTGCAGCAGACGTGGTTAAG	60	50	20
Lu2189	1	p3	(TAC)8	2	366 369	Lu2189F	GGTTTCGATCGATTGTACCTTTC	61	43.5	19+23
						Lu2189R	ACGGAGAGAGGAGGAGGAAG	59.9	60	20
Lu2193	1	p2	(TA)9	2	193 197	Lu2193F	TGCACGGAAGAGTTCATCAT	59.2	45	19+20
						Lu2193R	TTGTTGTTCTTGTTGCTGCTG	60.1	42.9	21
Lu2194	1	p2	(AT)22	6	277 279 281 283 285 301	Lu2194F	AACCTTCAACCCCAGTCTCC	60.3	55	19+20
						Lu2194R	CATCCTACCATGTGGCTTCC	60.3	55	20
Lu2195	1	p2	(TA)9	2	375 417	Lu2195F	CACTATCTCATGGCCTTCCAG	59.7	52.4	19+21
						Lu2195R	CGGCAAGTCATTCATCTGTC	59.2	50	20
Lu2196	1	p3	(TCA)6	2	null 292	Lu2196F	GGAGATTGCTGATGATTTTGC	59.7	42.9	19+21
						Lu2196R	TTACCCGCGTTTCACTTTTC	60.1	45	20
Lu2197	1	p3	(TCT)6	2	277 286	Lu2197F	ACATCAGCATCTCCCCACTC	60.1	55	19+20
						Lu2197R	GGATTCTGAAGGCGATTGAG	59.8	50	20
Lu2203	1	p2	(AT)12	2	381 383	Lu2203F	TTGGAGTTTTCATGCTTCACC	60.1	42.9	19+21
						Lu2203R	GCATGCAAATTCGATCCAC	60	47.4	19

Lu2206	1	p2	(AT)11	4	326 328 330 332	Lu2206F	GGGAGATTTTGGTTGTGGTG	60.2	50	19+20
						Lu2206R	TTTGGACGTTAGTCCACATCTC	59.1	45.5	22
Lu2207	1	p2	(AT)9	3	258 260 262	Lu2207F	GCGTGTGAAAACAGCAAAAG	59.5	45	19+20
						Lu2207R	GGTCCATGCTCGGTTTAGTC	59.6	55	20
Lu2208a	2	p3	(GAA)6	2	206 209	Lu2208F	TCTGAGTGGTCTGGCTTGG	60	57.9	19+19
Lu2208b				2	193 199	Lu2208R	CTCCCAGCAATCAAATCTCC	59.6	50	20
Lu2209	1	p3	(GAA)6	2	336 339	Lu2209F	TTGTTTCCCCAATTCATGTG	59.2	40	19+20
						Lu2209R	CACGTTTGGTGCCCTTTATC	60.4	50	20
Lu2214	1	p2	(AG)9	2	340 344	Lu2214F	TGGATTGGGAAATGAACAGG	60.7	45	19+20
						Lu2214R	AGGCAAAAACACTCGAAACG	60.3	45	20
Lu2216	1	p2	(AT)15	5	362 364 366 368 376	Lu2216F	CACGCTACGTACAAATGATATCTCTC	60.4	42.3	19+26
						Lu2216R	CCATTCCTCCAAGCAACAAG	60.6	50	20
Lu2217	1	p2	(AT)9	4	310 312 314 316	Lu2217F	AGTAAAATGAAAGCGCGAGA	57.8	40	19+20
						Lu2217R	TAAGATTTTTGCCTCCGTGT	57.4	40	20
Lu2219	1	p3	(CAA)12	2	385 387	Lu2219F	TCAGTTGGCTAGAGCGTGTG	60.2	55	19+20
						Lu2219R	TGGTTCAACATGGAATGCAG	60.5	45	20
Lu2220	1	p3	(CTC)8	2	275 284	Lu2220F	CTCCGCCAATACAAAACACC	60.4	50	19+20
						Lu2220R	GCAAGACCTCCGTTATGGAC	59.6	55	20
Lu2223	1	p3	(TGA)6	2	228 237	Lu2223F	AGTGTATGTGCCGGCTGTG	60.8	57.9	19+19
						Lu2223R	TGGACCGGACCTCAGTAAAG	60.1	55	20
Lu2230	1	c	(AT)13agtccacc(AT)10	4	314 316 318 320	Lu2230F	GGAGACTCAGAGCGGATTAGC	60.5	57.1	19+21
						Lu2230R	AAATGCTTGGATCCTTTTGC	59.2	40	20
Lu2233	1	p3	(AAT)12	3	209 212 215	Lu2233F	TTAAGGAAACGTCTCGTCCAG	59.4	47.6	19+21
						Lu2233R	CCACCCCTAGGACTGAATAATG	59.7	50	22
Lu2234	1	p3	(AGG)7	2	299 302	Lu2234F	TGGTATCGAAGGAGCCAAAG	60.2	50	19+20
						Lu2234R	ACGACTTGATTCCCAGATGC	60.1	50	20
Lu2235	1	p2	(AT)11	6	236 238 240 242 244 246	Lu2235F	TTGTCCAAACCAACCAAGTG	59.4	45	19+20
						Lu2235R	GACCACCGATTCTAACTCGAAC	60	50	22
Lu2237	1	p3	(GAA)9	3	255 264 267	Lu2237F	GACGGAGGAAAACAATCAGTG	59.6	47.6	19+21
						Lu2237R	GAAGAGAAGGGAGGGACCTG	60.2	60	20
Lu2239	1	p3	(TCT)9	2	380 392	Lu2239F	AGCCACTCAATTTGCGAAAC	60.3	45	19+20
						Lu2239R	CTGTTCCTCCCTGAAACGAG	59.8	55	20
Lu2241	1	p2	(AT)10	3	351 353 355	Lu2241F	AAGACAGACCATCCAAACTCG	59.2	47.6	19+21
						Lu2241R	ACTGATCAAGCAGGGAATCG	60.2	50	20
Lu2242	1	p2	(AG)9	2	296 298	Lu2242F	CACAGGGGTCCAGTTCTCAC	60.6	60	19+20
						Lu2242R	AGCAGCTGTTCCTGGAGATG	60.6	55	20
Lu2246	1	p2	(TA)9	2	381 383	Lu2246F	TTTGGTAGAGGCACCGTAGC	60.3	55	19+20
						Lu2246R	TCCCCTGTTCCTGAGCATAG	60.2	55	20
Lu2247	1	p2	(TA)16	5	244 278 280 282 284	Lu2247F	GAACAACGACCCATTTGGAC	60.2	50	19+20
						Lu2247R	AGAATGGAATTGGGGGAAAG	60.1	45	20
Lu2248a	2	p4	(AAAG)5	2	301 305	Lu2248F	GGGTAAACCAACCCCAAAAC	60.3	50	19+20
Lu2248b				2	276 284	Lu2248R	CACATTTCTTGGCCTTCCTC	59.7	50	20

Lu2250	1	p2	(AT)11	2	279 283	Lu2250F	AGAAGGAGGGTTCGGGTATG	60.3	55	19+20
						Lu2250R	TGTAGCTGCCCCAGAAAATC	60.2	50	20
Lu2251	1	p3	(ATA)10	3	null 201 207	Lu2251F	AGCGGCCTGTACATAATTGG	60	50	19+20
						Lu2251R	GGAAGAAGTCGAAGCTGTGC	60.1	55	20
Lu2252	1	p3	(GGA)7	2	192 198	Lu2252F	GGGTATGGCAGTGGATCG	59.9	61.1	19+18
						Lu2252R	GCCTGCTGCATCTTTATTCC	59.8	50	20
Lu2255	1	p3	(TCT)9	2	196 202	Lu2255F	GAGCTTCGGTTAGGCATTTG	59.8	50	19+20
						Lu2255R	CAAGGAATGCTTCATCGTTTG	60.6	42.9	21
Lu2256	1	p3	(AAG)16	2	266 284	Lu2256F	ATCATTGTTGCGTTGATTGG	59.4	40	19+20
						Lu2256R	CTGTGCAGGTTGTCATTTGG	60.2	50	20
Lu2257a	2	p2	(AT)10	2	359 361	Lu2257F	CCATATCAATTGGGCATCG	59.7	47.4	19+19
Lu2257b				3	335 337 339	Lu2257R	TCCAATCCACGAAAAGAAGG	60	45	20
Lu2258	1	p2	(AT)9	2	374 376	Lu2258F	TTCACATTGCACATGATTCG	59.1	40	19+20
						Lu2258R	AGCTTTTCCATTCCATTTCG	59.2	40	20
Lu2259	1	p3	(ATT)6	2	263 266	Lu2259F	ACTGCCTTTCTCCTCAAACG	59.5	50	19+20
						Lu2259R	AGATTGGTGAAATCGGATGC	59.9	45	20
Lu2260	1	p3	(GAA)7	2	284 287	Lu2260F	CAGGTGATGTGTGGGTTTTG	59.8	50	19+20
						Lu2260R	AGAAGTCGATCGGCTAATGG	59.3	50	20
Lu2262	1	p3	(TAA)8	3	186 189 192	Lu2262F	GGCAGTTTTTGTCTCTTTGACC	60.2	45.5	19+22
						Lu2262R	AAAAAGGAAGGTGGGTTTGC	60.3	45	20
Lu2264a	2	p2	(TC)11	2	313 315	Lu2264F	AGAGGAGCGCTGAGATTTTG	59.7	50	19+20
Lu2264b				2	290 292	Lu2264R	CGGCACAACAAAGGATTAGG	60.5	50	20
Lu2265	1	p2	(AG)14	2	null 215	Lu2265F	CATCGTCAGAAGCAGAATGG	59.4	50	19+20
						Lu2265R	TCAGTATGTGGGGATGAAAGC	59.9	47.6	21
Lu2266a	2	p3	(GAT)9	2	364 373	Lu2266F	TGAGCATTCAGCAAGGTTTG	60	45	19+20
Lu2266b				3	340 343 346	Lu2266R	TAAAAGGCCGCTGATCACTC	60.4	50	20
Lu2268a	2	p2	(AT)13	2	322 330	Lu2268F	GAACATATGTAACGGGTGAAACAC	59.6	41.7	19+24
Lu2268b				2	370 372	Lu2268R	AACGATCTCAAGGCTCAGTTC	58.5	47.6	21
Lu2270	1	p3	(CTT)8	2	348 354	Lu2270F	TGCAGAAAAACCCCATAACC	59.8	45	19+20
						Lu2270R	GAAGAGGACAACGGATGAGG	59.7	55	20
Lu2272	1	p3	(GAA)8	2	371 377	Lu2272F	ATTCTCCGACGAACTCAACG	60.3	50	19+20
						Lu2272R	ATGAACCTTGCTGGAGAACG	60.3	50	20
Lu2274	1	p3	(TTG)6	2	249 255	Lu2274F	GGTCTCCTTGAGCCTAGAACC	59.3	57.1	19+21
						Lu2274R	CAAAACTCCACGAGACATGG	59.1	50	20
Lu2275	1	p3	(CAG)6	2	291 297	Lu2275F	GCAGCAGCATAGTGAAATGC	59.6	50	19+20
						Lu2275R	ATTCGACTGTGGGAGTGAGG	60.1	55	20
Lu2278	1	p2	(TA)9	2	361 365	Lu2278F	GCTCCTTGTATGCCCTTGTC	59.7	55	19+20
						Lu2278R	TTACACGGCAAAGCTGTCTG	60	50	20
Lu2279	1	p3	(TAA)10	3	204 210 216	Lu2279F	TTCAAGGAAGGGAGTATTCTGG	59.6	45.5	19+22
						Lu2279R	CCAACACTATAAACAAAGTCGTGTC	59.1	40	25
Lu2284	1	p2	(TA)11	2	248 252	Lu2284F	AAGAGAGGGTTGGGGTATGG	60.2	55	19+20
						Lu2284R	ATGGTTGCCAAAATGAGAGG	59.9	45	20

Lu2286	1	p2	(AT)11	3	364 366 368	Lu2286F	GACAAATGTGGGGATCTTCG	60.3	50	19+20
						Lu2286R	ATTGTTTCCTCTTGCCATCG	60.1	45	20
Lu2287	1	p3	(CAA)6	2	197 200	Lu2287F	AGCATCTCTCCATCTTCCTCAC	59.8	50	19+22
						Lu2287R	GAGTGTCGGACCAAAGGTTC	59.6	55	20
Lu2288	1	p2	(AT)10	5	247 249 251 253 255	Lu2288F	CACGATTGGATAACATCATGG	58.7	42.9	19+21
						Lu2288R	CCACCGGAATTATTGTTGC	58.8	47.4	19
Lu2291	1	p2	(AT)10	2	363 365	Lu2291F	CATGTTCCGAAAGGAGTACACA	60	45.5	19+22
						Lu2291R	GAGCCAAGAGGATCAAGTGG	59.8	55	20
Lu2292	1	p2	(AT)20	8	342 350 354 358 362 366 368 370	Lu2292F	TTGGTCGGTTTTTATGAGACG	60	42.9	19+21
						Lu2292R	ATGGTTAGCTGGGGATCAAG	59	50	20
Lu2293	1	p5	(GTCTC)6	3	284 304 314	Lu2293F	AGGTCACTCCATTCGGACAC	60	55	19+20
						Lu2293R	CCTCGTCGTGGAGAATCATC	60.6	55	20
Lu2295	1	p2	(TA)13	5	353 357 359 361 367	Lu2295F	CATTATGTTTTTACTAACGCGGTTC	60.2	36	19+25
						Lu2295R	CAGTCCGTTTTTACTTCATGC	57.4	42.9	21
Lu2296	1	p2	(TA)9	3	355 357 359	Lu2296F	CCAGCAGATCGTTTTGTGAC	59.3	50	19+20
						Lu2296R	ATTCCGCTCCATAACCAATC	58.9	45	20
Lu2297	1	p3	(TGA)7	2	271 274	Lu2297F	AAGCACGTATGGGGTGTAGC	60	55	19+20
						Lu2297R	CCAAACACAGACAAGATTGAGG	59.6	45.5	22
Lu2331	1	c	(ATC)6(AGC)6	2	204 221	Lu2331F	GAGTTCGGAGCAAACAGACC	59.9	55	19+20
						Lu2331R	GAAATCGGTCCTTCATCTCG	59.6	50	20
Lu2332	1	p6	(CCTAAT)9	6	249 255 261 267 279 345	Lu2332F	GAGGAAACCTAGCGGAGTAGC	59.5	57.1	19+21
						Lu2332R	GAGCGCCTAAGGCTGAATC	60.1	57.9	19
Lu2333	1	p3	(GAA)8	2	262 265	Lu2333F	TTCTGAGATTCGAGCTGTGG	59.1	50	19+20
						Lu2333R	GGTTTCCACCCCTATACACG	59.2	55	20
Lu2340	1	p2	(AT)11	3	272 276 278	Lu2340F	AACATAGGGTTTAAAAGTTGTTGTG	57.8	32	19+25
						Lu2340R	TTTATGTTAAGAGTTACGGTTTGTTG	58	30.8	26
Lu2341	1	p3	(CTT)7	2	351 354	Lu2341F	CTGGTGGTTGATGCTAGTGC	59.3	55	19+20
						Lu2341R	AAATGGGGGACTTGATTTAGC	59.3	42.9	21
Lu2342	1	p2	(AT)9	2	346 354	Lu2342F	GGGGAGCTGAGTACAGTTGC	59.9	60	19+20
						Lu2342R	GGACCTAATGAAGAAGATTTCTGG	59.5	41.7	24
Lu2344	1	p3	(AGA)13	2	296 307	Lu2344F	GCTTGGATCTGAGCGAAGAG	60.2	55	19+20
						Lu2344R	AAACCAACGCAACAAAGGAG	60.1	45	20
Lu2345	1	p3	(AGT)6	2	191 197	Lu2345F	GGAAACGGACAAGAGAATTGG	60.8	47.6	19+21
						Lu2345R	CTCATAGCCAGGTGGACTGC	60.8	60	20
Lu2346	1	p2	(AT)11	6	245 247 251 255 265 271	Lu2346F	GAAAAGCAAAGAAGCTGAAAGG	59.7	40.9	19+22
						Lu2346R	TTGGCCAAAATCACTCACC	59.5	47.4	19
Lu2347	1	p3	(ATA)7	3	360 363 366	Lu2347F	ACGTCATGTCCTCCACGTC	59.5	57.9	19+19
						Lu2347R	GACGAGGGAAAGTTGTGCTC	59.9	55	20
Lu2349	1	p3	(CTG)6	2	306 312	Lu2349F	GTGGGTATTTGGAGCAGAGC	59.7	55	19+20
						Lu2349R	CAACTCCATAACCCCTCAGC	59.5	55	20
Lu2351	1	p2	(TA)16	6	282 292 298 304 310 314	Lu2351F	GGAAGCGAGTCATTCAATACG	59.7	47.6	19+21
						Lu2351R	GCTGCGTAGCTACAATTTGATTAC	59.4	41.7	24

Lu2352	1	p3	(TCA)7	2	183 198	Lu2352F	CCCTCATTTCCAATGTCCTC	59.3	50	19+20
						Lu2352R	GCAAAGGAAATCTGAGCTTGA	59.6	42.9	21
Lu2354	1	p2	(TA)13	5	315 317 319 321 325	Lu2354F	AGTCAGCCCACTCTGAATCC	59.3	55	19+20
						Lu2354R	GGAGCCCTTTTCCCATTAAC	59.8	50	20
Lu2355	1	p3	(TGA)7	3	260 266 269	Lu2355F	TTACTCGGAGAGCGAGAAGC	59.9	55	19+20
						Lu2355R	TAATCTCTTCACCCGGTTCG	60.1	50	20
Lu2357	1	p2	(TA)12	3	271 273 275	Lu2357F	ATCGACGAGTGGGTACAAGG	60	55	19+20
						Lu2357R	AAAAGGAAGCAGATTAATGATTG	56.7	30.4	23
Lu2358	1	p3	(CAT)9	3	301 304 316	Lu2358F	ATGTCGTCCATCCAGTCTCC	59.9	55	19+20
						Lu2358R	GACACCCATAAGCATGATTGC	60.4	47.6	21
Lu2360	1	p3	(CTA)6	2	294 300	Lu2360F	ACGAATCCAGGCAAATCAAG	60.1	45	19+20
						Lu2360R	ATGATGGTAAGGGTGGCAAG	59.8	50	20
Lu2361	1	p2	(AT)11	2	null 193	Lu2361F	ACCAGGGGTAAAAGAGAACG	58.2	50	19+20
						Lu2361R	ACAAAGGCAAGTTGTGCAAG	59	45	20
Lu2362	1	p3	(TCT)9	2	300 306	Lu2362F	TGATCCTCTCCCTCACTCTCC	60.7	57.1	19+21
						Lu2362R	GAAGAGGGGCCAGAAATAGG	60	55	20
Lu2364	1	p2	(AG)18	4	312 316 318 320	Lu2364F	TGTTGCCTCATCTCCCTAGC	60.4	55	19+20
						Lu2364R	TTGCCGAATCTGCTTTTACC	60.2	45	20
Lu2365	1	p2	(TC)20	5	298 300 304 306 308	Lu2365F	AACTGAAGAACTCGGCGAAC	59.5	50	19+20
						Lu2365R	CGTAGCACCCATTCCATATTTAG	59.8	43.5	23
Lu2366	1	p4	(TAAT)6	2	214 218	Lu2366F	TGCGTAGTTGACACCGTGAG	60.9	55	19+20
						Lu2366R	ACACCATGGTCGACGAAATC	60.8	50	20
Lu2370	1	p2	(TA)9	2	257 259	Lu2370F	GGAAAACATGTGCGAGATTG	59.1	45	19+20
						Lu2370R	TGGCACTGAAGCATTTTACATC	60.1	40.9	22
Lu2371a	2	p3	(CTT)7	2	224 227	Lu2371F	ATCTCAGCCCCTTGAAATCC	60.4	50	19+20
Lu2371b				2	null 217	Lu2371R	CACCACCTCCTCATCGACTC	60.7	60	20
Lu2373	1	p2	(TA)9	3	274 276 278	Lu2373F	TTTTGGACCAACTCCACCTC	59.9	50	19+20
						Lu2373R	GATACTTAGCCCGCATCAGC	59.8	55	20
Lu2374a	2	p3	(TCA)11	2	210 213	Lu2374F	GGAATGCCTTGAGTGTTTGG	60.5	50	19+20
Lu2374b				2	204 207	Lu2374R	AAGACTATTTTCGGGCTTCTCC	60.1	45.5	22
Lu2376	1	p5	(TGATG)5	2	null 223	Lu2376F	TTGAAATCGTACCTCGTGTCC	60	47.6	19+21
						Lu2376R	CGTCGACGGCTGAGTTATTC	60.8	55	20
Lu2377	1	p4	(TGTT)5	2	283 285	Lu2377F	GCGGAGCTAGGAATGTGAAG	60	55	19+20
						Lu2377R	AATTGTCAGGAGGCATCCAC	59.9	50	20
Lu2382	1	p2	(TA)9	3	330 336 339	Lu2382F	TCAAATTCTAATGTTGACCCTTACC	59.7	36	19+25
						Lu2382R	TCTTGACGGAACCAGGAAAC	60.1	50	20
Lu2383	1	p2	(TC)9	2	null 306	Lu2383F	AAACTGCTTTTGTTGGTGGTG	60.1	42.9	19+21
						Lu2383R	TGAGGTAGTGAGGGCCAAAC	60.1	55	20
Lu2387	1	p2	(AT)16	5	344 346 348 350 354	Lu2387F	AGGCCTTTGTTTTTGGTAGTG	58.3	42.9	19+21
						Lu2387R	GAACCAATGGGAATTTGTTTTC	59.6	36.4	22
Lu2388	1	p3	(ATT)7	2	277 280	Lu2388F	AACTGGTGAAAATTATACCATAGAACC	59.1	33.3	19+27
						Lu2388R	ACTCCAAACCGAACACCAAG	60	50	20

Lu2390	1	p2	(GA)9	2	255 267	Lu2390F	CGGCGCTCTGTATTTCTCTC	60.1	55	19+20
						Lu2390R	ATCCTCAGCCACCACAACTC	60.1	55	20
Lu2392a	2	p2	(TA)11	3	244 246 248	Lu2392F	TTCCCTGTCTCTCGAGTCTGA	60.1	52.4	19+21
Lu2392b				4	225 229 231 233	Lu2392R	CCGAGTTGCAGAAAGTCTCC	60	55	20
Lu2393	1	p2	(TA)9	3	352 354 358	Lu2393F	CTTTGTCCTTGCAGTTGTGG	59.3	50	19+20
						Lu2393R	AAGTGGCGGTCTATATGAAATG	58.2	40.9	22
Lu2394a	2	p2	(TC)17	3	360 362 364	Lu2394F	GGTTCCAATGAACCGAAGAC	59.4	50	19+20
Lu2394b				3	331 339 341	Lu2394R	CGGAATCTAGAAGCACAAGACC	60.3	50	22
Lu2396	1	p3	(ATA)16	5	354 357 360 366 372	Lu2396F	CTACGGGACAAAGCAGTAACG	59.8	52.4	19+21
						Lu2396R	TGCATCTCATCGACTCATCC	59.8	50	20
Lu2397	1	p4	(GAGT)5	2	325 329	Lu2397F	TTTTCTTTGAGCTGCTTGTCC	59.6	42.9	19+21
						Lu2397R	GGCCTCAATCGTCAGAAAAG	59.8	50	20
Lu2399	1	p2	(TA)9	3	313 321 323	Lu2399F	AACGGAGACGGAGAAGTACG	59.4	55	19+20
						Lu2399R	GCCCACACACAACACTCG	59.7	61.1	18
Lu2400	1	p3	(TCT)6	2	245 248	Lu2400F	CCAATTCCCCTTTAAACATAGG	58.8	40.9	19+22
						Lu2400R	TTATCCAGTTCGGTCTGCTG	58.9	50	20
Lu2401	1	p3	(TTA)8	2	221 227	Lu2401F	TCGCAAATCTCAAATCATGC	59.8	40	19+20
						Lu2401R	TGAAATGGCGTACACAAAGC	59.7	45	20
Lu2403	1	p2	(AT)12	4	321 323 325 327	Lu2403F	TCAACCTCTCTCCGATCCAC	60.2	55	19+20
						Lu2403R	ATTGAAAATGGACGGTGGTG	60.6	45	20
Lu2404	1	p2	(AT)18	2	291 301	Lu2404F	GGTGTCCCACACCCTTCTC	60.4	63.2	19+19
						Lu2404R	GCGCCTACTTGTTTGAATGG	60.6	50	20
Lu2405	1	p2	(AT)20	2	309 311	Lu2405F	CAAGCAAAGCAACTTGAAACC	59.9	42.9	19+21
						Lu2405R	TTACCACCGACAATGATTCG	59.4	45	20
Lu2408	1	p3	(CAT)6	3	292 298 301	Lu2408F	CACTCTTTTTGGTGGGTTCG	60.5	50	19+20
						Lu2408R	AGAGGGAGCAAGAGGAGGAG	60.1	60	20
Lu2409	1	p2	(CT)15	3	277 279 283	Lu2409F	ACGGCCAATTTAAGAAGCTG	59.4	45	19+20
						Lu2409R	ATATCATAACCGCCGACCAG	59.8	50	20
Lu2410	1	p3	(CTT)6	2	287 290	Lu2410F	AAGGACTGCCATCAACCAAC	60	50	19+20
						Lu2410R	TCTCTCCCGTGTCATTGAAAG	60.2	47.6	21
Lu2411	1	p3	(GAA)11	2	339 336	Lu2411F	TGGTGATGATGATGGGTAGC	59.3	50	19+20
						Lu2411R	CTTCACCAAAGCAAACTCTCC	59	47.6	21
Lu2414	1	p3	(GGA)7	2	254 263	Lu2414F	CACAGCTTCAGCAAGATAGGC	60.2	52.4	19+21
						Lu2414R	TCCTCAACTCCCCACTATGC	60.1	55	20
Lu2418	1	p4	(AATT)5	2	244 248	Lu2418F	GTGAACGTGGTGGAGAAAGG	60.5	55	19+20
						Lu2418R	AAAATATATCACCGCCGTTCC	60.1	42.9	21
Lu2420	1	p3	(CAG)10	3	296 307 316	Lu2420F	TCCCACCTGGATAAAACTCG	59.9	50	19+20
						Lu2420R	CATCGCCATGGATGAGAAG	60.2	52.6	19
Lu2421	1	p3	(TAA)9	3	270 276 282	Lu2421F	TAACACCCACTCCCCTTCAC	59.8	55	19+20
						Lu2421R	TCAAAGGAGCCATCTCAACTC	59.4	47.6	21
Lu2422	1	p3	(TAA)13	3	264 267 270	Lu2422F	CTTGACAACTGCTTCCCACTTAC	60.2	47.8	19+23
						Lu2422R	TGTCACGTGAGTTTGGCTTC	59.9	50	20

Lu2424	1	p5	(CTCTT)7	5	295 300 305 315 350	Lu2424F	TGGCCAAGTTCGTTGTAGTG	59.8	50	19+20
						Lu2424R	TTGTACGCCTCAGCAGTGTC	60.1	55	20
Lu2425	1	p4	(ATAA)6	2	248 256	Lu2425F	GTTTGGGAGGCAATATCAGG	59.4	50	19+20
						Lu2425R	ACAATTCATGCAAACGCATC	59.6	40	20
Lu2426	1	p5	(AACCC)5	2	193 198	Lu2426F	AATCCGGTCCGACCTTTATC	60.2	50	19+20
						Lu2426R	TCGGTTTAGGGAAAGTAGAGAGC	60.3	47.8	23
Lu2428	1	p3	(AAT)12	3	345 359 362	Lu2428F	ATGCAGACTTCATCGCAGTG	60	50	19+20
						Lu2428R	AATGATGGCACTGGGAACTC	59.9	50	20
Lu2429	1	p3	(ATA)6	2	272 275	Lu2429F	ATTCCAATGCCATCACAGC	59.5	47.4	19+19
						Lu2429R	AGTGAGTGGTGGGTCTCTGG	60.2	60	20
Lu2430	1	p3	(CCG)6	2	240 243	Lu2430F	GGAATCTCCTGAAATGCTTCC	60	47.6	19+21
						Lu2430R	GGATGAGTCGGATTCAGAGG	59.6	55	20
Lu2431a	3	p4	(GGAA)5	2	null 347	Lu2431F	GGAGCAAGCAGAAGAGTTGC	60.3	55	19+20
Lu2431b				2	322 329	Lu2431R	CCTCAACTCCTCGTTTCAGTG	59.9	52.4	21
Lu2431c				2	null 305					
Lu2432	1	p4	(GGTT)5	2	296 300	Lu2432F	GTCTTTGGGGGAACATTGAG	59.4	50	19+20
						Lu2432R	TCCTCGTTCAGCATGTCATC	59.8	50	20
Lu2433	1	p2	(TA)10	2	344 346	Lu2433F	GAAGAGTGAAGGCGAGTTGC	60.1	55	19+20
						Lu2433R	TTGACAGAGCCCTTGAAAGC	60.5	50	20
Lu2437	1	p3	(AGA)8	3	218 221 224	Lu2437F	ACAAGTTCGACGACCTGACC	60.2	55	19+20
						Lu2437R	AGAGCCACAAACAGCAACG	60	52.6	19
Lu2438	1	p3	(CAG)6	2	333 336	Lu2438F	TGCAGAGAGTGGTGCAAGAG	60.3	55	19+20
						Lu2438R	TGAGCTTGAGCTTGTGCTTG	60.5	50	20
Lu2439	1	p3	(AGA)6	2	213 222	Lu2439F	CTTTCCGATGGAAAATCTCG	59.6	45	19+20
						Lu2439R	AGGACACCTCGTTACCTTGC	59.2	55	20
Lu2440	1	p3	(AGT)7	7	215 218 221 227 230 233 236	Lu2440F	GACTGTAGAGGCAGGCAAGG	60	60	19+20
						Lu2440R	TGAAGCAACGTACGGTATGG	59.6	50	20
Lu2442	1	p2	(AT)12	4	279 281 283 285	Lu2442F	CACGCTTCGTTTTAGAAGTGG	59.9	47.6	19+21
						Lu2442R	GTGTGTCAAACATACAAGAGTGAGC	60.1	44	25
Lu2443	1	p2	(AT)9	2	350 352	Lu2443F	CCAAGTAACCCGAAAGTCTCC	60	52.4	19+21
						Lu2443R	GTCGTATCCATCAACCACTGC	60.4	52.4	21
Lu2445	1	p3	(GAA)7	2	293 298	Lu2445F	ACATTTTCCGCCCTTCTCTC	60.6	50	19+20
						Lu2445R	ACCCAGCAAGTAACCCAATG	59.9	50	20
Lu2446	1	p3	(GAC)7	4	256 259 262 265	Lu2446F	ACATCGAATCCTGTGCAGTG	59.7	50	19+20
						Lu2446R	GAGCTTCAATTGCCGAAAAG	60	45	20
Lu2447	1	p3	(GAT)7	3	287 302 305	Lu2447F	TGCTCTGGCAACCGATAAG	60	52.6	19+19
						Lu2447R	TGGTTAGCTTGGTCGGAATC	60.1	50	20
Lu2448	1	p3	(GCT)10	2	356 368	Lu2448F	ATTGATATTGGCTCGGATGG	59.7	45	19+20
						Lu2448R	GACTTGGCCTCCGATCTATG	59.7	55	20
Lu2449	1	p2	(TA)11	3	191 193 195	Lu2449F	CATGTCCTTTCATCAGTGTGG	59	47.6	19+21
						Lu2449R	CTTGGCGATAACCTAAATTGG	58.6	42.9	21
Lu2450	1	p2	(TC)10	3	285 287 291	Lu2450F	TGACAAGAAAGCACCAAACG	59.9	45	19+20

						Lu2450R	AGGGAGGCTTTTTCTCTTGG	59.8	50	20
Lu2451	1	p2	(TC)9	2	241 247	Lu2451F	TCGGACCTCTTTTTCTTAGGG	59.7	47.6	19+21
						Lu2451R	CCAACAACACCGAAGAACG	60.1	52.6	19
Lu2453	1	p3	(TTC)7	3	261 263 265	Lu2453F	GATGAGGGGATGGTTTGTTG	60.2	50	19+20
						Lu2453R	CTCTCCTCCCTCCCTTCACT	59.8	60	20
Lu2456	1	p2	(TA)13	5	300 310 312 314 316	Lu2456F	TGATGATAGACGAAATGTGTGG	58.5	40.9	19+22
						Lu2456R	CGAAAATAGCTCCACAATGG	58.2	45	20
Lu2457a	2	c	(AG)15atagatagagagagagag(AT)14	8	267 270 272 274 276 278 284 288	Lu2457F	CTCCTTCTTAGCCGATTTTGC	60.3	47.6	19+21
Lu2457b				3	245 249 251	Lu2457R	GTCGATCGATTGGAGATTGC	60.6	50	20
Lu2459	1	p3	(ACA)6	3	258 270 273	Lu2459F	GAGTCTCCATATAGTCAGGATGAGC	59.7	48	19+25
						Lu2459R	TTTTGATTAGGAGCCGTTGG	60.1	45	20
Lu2463	1	p2	(AT)20	6	260 262 270 272 274 276	Lu2463F	GGATTTGTCTGGTGGGTTTG	60.2	50	19+20
						Lu2463R	TCATTTCAAGGGGAGCAAAG	60.2	45	20
Lu2464	1	p3	(AGA)6	2	272 275	Lu2464F	TGATTGGGGGATTGGTAGAG	59.7	50	19+20
						Lu2464R	CCACCTCACCGCTTTCTTC	60.8	57.9	19
Lu2465	1	p3	(CAT)7	2	334 349	Lu2465F	GACCGAGCGAATTTCTTGAG	60	50	19+20
						Lu2465R	GGCCTTATCCCTGACTCTCC	60	60	20
Lu2466	1	p2	(CT)9	2	247 249	Lu2466F	ATCGCTTCAATCGGTCTCTG	60.4	50	19+20
						Lu2466R	GAACATGGCGTTTTCCACTC	60.5	50	20
Lu2467	1	p2	(CT)9	2	309 319	Lu2467F	TAGGGAATGCAGTTGCTGTG	59.9	50	19+20
						Lu2467R	AGCAGCCAGCTGGTAGAGAC	59.8	60	20
Lu2468	1	p3	(CTT)16	2	437 443	Lu2468F	CAGCGATTCTCTTTGTTCAGC	60.1	47.6	19+21
						Lu2468R	CAAGTGATCTTGCATTTTCTGC	59.9	40.9	22
Lu2469	1	p3	(GAT)6	3	360 368 371	Lu2469F	GATTTGATGTTGCCAGTTGC	59.1	45	19+20
						Lu2469R	AGGCCTTTTTCATTGACAGC	59.3	45	20
Lu2472	1	p3	(CTT)11	2	352 355	Lu2472F	TAAATGTTTCCCGCCAAAAC	59.8	40	19+20
						Lu2472R	TTTGGAAATGGGAAGTGAGG	59.9	45	20
Lu2473	1	p2	(AT)14	6	362 368 372 376 378 380	Lu2473F	GCAGTGTTTGAGGGTTATGG	58.1	50	19+20
						Lu2473R	AATAACAAACAGCCCAACTCC	58.1	42.9	21
Lu2474	1	p2	(TA)9	2	290 300	Lu2474F	CCTCATCCCAGACTTTGACC	59.5	55	19+20
						Lu2474R	TCCTTAATGCTATGTCCTGAACC	59.5	43.5	23
Lu2478	1	p2	(TA)9	3	338 352 354	Lu2478F	ATCCATGGCTTCTCCAACC	59.9	52.6	19+19
						Lu2478R	CCCAAACAATCTCATCTTTGC	59.6	42.9	21
Lu2479	1	p2	(AT)10	2	null 338	Lu2479F	CAAGGTGCCATGGTTAAGG	59	52.6	19+19
						Lu2479R	GCTACGACACAGAGCAGTGG	59.6	60	20
Lu2482	1	p2	(CT)18	5	280 280 282 284 290	Lu2482F	AGGCAGTCCTGCTATTCGTC	59.5	55	19+20
						Lu2482R	GCCAATAGCCATGTGAGTAGG	59.6	52.4	21
Lu2483	1	p3	(GAA)15	3	214 217 233	Lu2483F	TGTTGTAATATATGAAAAGAGAAATGGAAG	60.4	26.7	19+30
						Lu2483R	TTACAAGGTGGGGGAGTTTG	59.8	50	20
Lu2485	1	p2	(TA)15	4	190 192 194 198	Lu2485F	TTCCTTCCCACTTGGTTTTG	59.9	45	19+20
						Lu2485R	GACTCACTTCCATTGCGTCTC	59.9	52.4	21
Lu2486	1	p2	(TC)18	5	211 213 215 217 227	Lu2486F	TTCACCATCACACCTTCACC	59.4	50	19+20

						Lu2486R	TTTGTTTGATGTTCTGGACCTG	60	40.9	22
Lu2489	1	p3	(ACA)8	2	320 332	Lu2489F	CATGCATGCTAACACACAAGG	60.2	47.6	19+21
						Lu2489R	GATCTCGTCAGAAGGAAAAGTAGC	59.9	45.8	24
Lu2490	1	p2	(AT)11	2	192 206	Lu2490F	GCCATGTGTGAAAGGTGATG	60	50	19+20
						Lu2490R	ATATGGTCAAGGGGCAAGTG	59.8	50	20
Lu2492	1	p3	(GCT)6	2	322 328	Lu2492F	CTCGAGTAGCAGTGGTGGTG	59.5	60	19+20
						Lu2492R	ATTCGAGGGCATCAGTGAAC	60.1	50	20
Lu2493	1	p3	(AAG)6	2	340 343	Lu2493F	TGCAACCTTATGAAGGTCAGG	60.1	47.6	19+21
						Lu2493R	AGGTGAATCAACTGGGTTGC	60	50	20
Lu2495	1	p2	(AT)12	3	206 210 214	Lu2495F	CCAACGCTGAATACAAGGTG	59.2	50	19+20
						Lu2495R	AGGCTGAAGCTTTTGATTCG	59.6	45	20
Lu2496	1	p3	(ATA)6	2	322 334	Lu2496F	ACGGTGAAATACCGGTTACG	59.7	50	19+20
						Lu2496R	AAATTCGCATCCTCGACAAG	60.2	45	20
Lu2497	1	p3	(CCT)7	2	232 250	Lu2497F	GGAGATAGCCGATCAGATGC	59.8	55	19+20
						Lu2497R	AAGCCAAAGGAAGAAGAGGTG	59.9	47.6	21
Lu2500	1	p3	(CTT)10	2	206 209	Lu2500F	ACCTGGGTCCAGAAATTGG	59.8	52.6	19+19
						Lu2500R	GAAGAGATGCGTCGTCAGC	59.7	57.9	19
Lu2501	1	p2	(GA)12	3	254 258 260	Lu2501F	CCAGCTGATCCAGTTCTTCC	59.8	55	19+20
						Lu2501R	GAGCGCACCAATTCAATTTC	60.6	45	20
Lu2502	1	p2	(GA)16	2	224 228	Lu2502F	TGCTCACCTTTTCCAAGTCAG	60.4	47.6	19+21
						Lu2502R	TGTTTGGGTGGGAGCTATTC	59.9	50	20
Lu2503	1	p3	(GAA)10	3	327 330 333	Lu2503F	CCTCGCAATGAATGTGTGAC	60.1	50	19+20
						Lu2503R	ACTTTGGTTGTTTGGCCTTG	60	45	20
Lu2508	1	p3	(ATG)6	2	237 240	Lu2508F	GGAGGATGGAAGCTATGCTG	59.8	55	19+20
						Lu2508R	CAACAGCAGCAGGAGTTGAC	59.6	55	20
Lu2509	1	p2	(AT)12	5	282 284 290 292 294	Lu2509F	AGAGGAAGGTGTGTCACTTGC	59.4	52.4	19+21
						Lu2509R	GTTCGATGTTTGGGTTATTGC	59.3	42.9	21
Lu2511	1	p2	(AG)11	3	204 208 212	Lu2511F	GCCGAATTCTGCGGTATATG	60.4	50	19+20
						Lu2511R	ACAAAGGCGTTGGACAAGAG	60.3	50	20
Lu2512	1	p2	(AG)17	4	190 192 194 196	Lu2512F	AAGCCAACAAGACAGAAGAACC	59.8	45.5	19+22
						Lu2512R	ATAGGGCGAGAAAGGCTAGG	59.8	55	20
Lu2513	1	p2	(AT)14	6	null 321 323 327 331 339	Lu2513F	GGGACAATATTTCAAATCATAAAAGG	60.2	30.8	19+26
						Lu2513R	GAAATTGATTGGATGTGGTTCC	60.4	40.9	22
Lu2515	1	p4	(CTTT)5	2	316 321	Lu2515F	GTGAATGGTTGGGAATCCTG	60.2	50	19+20
						Lu2515R	ACCATCGGCATACTCACCTC	60	55	20
Lu2516a	2	p2	(GA)11	4	326 330 332 340	Lu2516F	ACACCACAAACCACCCTACAG	59.8	52.4	19+21
Lu2516b				3	312 326 332	Lu2516R	TGGTTTTCCCAATTCAAAGC	59.9	40	20
Lu2517	1	p3	(GAA)7	2	197 203	Lu2517F	AGCTGCTGGCTAAGATCGAG	59.9	55	19+20
						Lu2517R	AGCAGGGGAACTCAACAATG	60.1	50	20
Lu2519	1	p3	(TAT)10	2	325 328	Lu2519F	CCACGTTACCTTGTTGTTGG	58.9	50	19+20
						Lu2519R	TCATTTTTAGGCTGCTGTCG	59.1	45	20
Lu2520	1	p3	(TTA)6	2	206 218	Lu2520F	TGGTGGCTGCTTTTGATTC	59.8	47.4	19+19

						Lu2520R	TCGCTTGGGTTGGTTAATTC	59.9	45	20
Lu2522	1	p3	(AGA)6	2	334 340	Lu2522F	AAAACGAACCGGTCTACTCG	59.2	50	19+20
						Lu2522R	TCGAATACGTCACGATCACC	59.5	50	20
Lu2523	1	p4	(ATCT)5	3	278 286 294	Lu2523F	CAATTTCAGTGCGTGGTACG	60.2	50	19+20
						Lu2523R	TCCCCACTCTTTTTGTCCTG	60.1	50	20
Lu2524	1	p2	(TC)10	2	350 356	Lu2524F	CTGGCCAAATTGAATAGTTGC	59.6	42.9	19+21
						Lu2524R	GGTCATAAATGGGCTTGACC	59.2	50	20
Lu2527	1	p3	(CAG)8	2	null 273	Lu2527F	CCACCATACAGCATCACTGC	60.1	55	19+20
						Lu2527R	TTTACCTTCATGAGGCAATGG	59.9	42.9	21
Lu2529	1	p2	(GA)10	3	271 276 279	Lu2529F	GACAGCCTGGAGCATAAACC	59.7	55	19+20
						Lu2529R	TCGGACACAGTACACACACG	59.2	55	20
Lu2530	1	p3	(GCG)8	2	206 210	Lu2530F	AATGCTCCGCGTAAAACAAC	60.1	45	19+20
						Lu2530R	CGCTCACAAGAGGGAAAGAC	60	55	20
Lu2532	1	p3	(AGA)11	2	280 283	Lu2532F	GGATAGAAGCTCACCGATGC	59.8	55	19+20
						Lu2532R	TTCAGAGCACCAGCAGAAAA	59.7	45	20
Lu2533	1	p2	(AT)10	3	227 231 233	Lu2533F	ATGAGAAAGGGGGCATTTG	59.9	47.4	19+19
						Lu2533R	CGAACCAAAGCGAAGAAAAC	59.9	45	20
Lu2534	1	p3	(ATA)8	3	279 312 318	Lu2534F	TCACATGGTACCCATTTTGG	59.1	45	19+20
						Lu2534R	GTTGCCGATTGTTAGTTCAGG	59.6	47.6	21
Lu2535	1	p3	(ATT)7	2	238 247	Lu2535F	ACGACATCCCTAAAACGAACC	60.2	47.6	19+21
						Lu2535R	CATGCCATTGTTTTGATTGC	59.9	40	20
Lu2536	1	p3	(CCG)7	3	190 193 199	Lu2536F	TCGATGTAACTCCCAACTTCG	60.1	47.6	19+21
						Lu2536R	ACTACGTCGTTGACGCTGTG	60	55	20
Lu2538	1	p4	(GTCA)5	2	352 364	Lu2538F	AGGACGATGAGGAGTTCAGTG	59.3	52.4	19+21
						Lu2538R	CTCCTCCTGTGTCTGTCACG	59.4	60	20
Lu2540	1	p3	(TTC)7	2	null 287	Lu2540F	CAGTTTCCCTCTTCGTACCG	59.7	55	19+20
						Lu2540R	GACATCCGCAGATGACTACG	59.3	55	20
Lu2541	1	p2	(AT)10	2	356 366	Lu2541F	GTAAGCTCCTGGCTTGATGC	60	55	19+20
						Lu2541R	CCTCCTTCAGCTACTCCTTCC	59.5	57.1	21
Lu2542	1	p2	(AG)12	2	337 339	Lu2542F	TTGGGAGGAGAACAAGAAGG	59.3	50	19+20
						Lu2542R	ATTCAAAGGGCGATTACGTC	59	45	20
Lu2543	1	p3	(AGA)6	2	240 243	Lu2543F	ATTGGGCTCTGTGGTAGCC	60.1	57.9	19+19
						Lu2543R	GCCCAAATCTTCTTAATTGTGC	60	40.9	22
Lu2544	1	p2	(TA)10	3	272 274 276	Lu2544F	GGTGTTCTGATAACGTGTTTCG	59.5	45.5	19+22
						Lu2544R	AGAAAGGCCCTGATTCTGC	59.4	52.6	19
Lu2545	1	p3	(ATT)6	3	330 336 339	Lu2545F	TGCTTTGCTAATTTATTATGGTGAG	58.9	32	19+25
						Lu2545R	ATGGTAGCTGGTGGGTGAAC	59.9	55	20
Lu2546	1	p2	(AT)9	2	334 339	Lu2546F	AGAAGGAGAACGGGGCTTAG	59.8	55	19+20
						Lu2546R	TCCTGGGATTAAACCAAAACC	60	42.9	21
Lu2547	1	p2	(CT)9	4	256 260 266 274	Lu2547F	TGAGAACAACCCGGAAAGAC	60.1	50	19+20
						Lu2547R	ACCCCTGAGACCTCCTATGG	60.3	60	20
Lu2548	1	p4	(CTCC)6	2	341 345	Lu2548F	CACCTACATAATCAGCGTCGTC	59.7	50	19+22

						Lu2548R	ATCGCAAGGAAAAACAGAGG	59.3	45	20
Lu2549	1	p3	(GAA)8	2	220 223	Lu2549F	CTAATTGCTCCTCCGATTGC	59.8	50	19+20
						Lu2549R	CTAACAGTTGCGGGAAGTTTG	59.8	47.6	21
Lu2550	1	p3	(TTC)6	2	366 369	Lu2550F	CCTTTCCTTCCTCCATTTCC	59.9	50	19+20
						Lu2550R	GCATATTCCCTCACCTCAGC	59.7	55	20
Lu2551	1	p3	(AAT)8	2	275 278	Lu2551F	TGCAGAAAGGAGGAGTACGG	60.4	55	19+20
						Lu2551R	CATCAAAGGAAGCGAAGAGG	59.9	50	20
Lu2552	1	p3	(ATG)7	2	228 231	Lu2552F	GGAAAACCAATCTGCTCGAC	59.7	50	19+20
						Lu2552R	AGCAGAACCAACCCAGTAGG	59.2	55	20
Lu2553	1	p2	(CT)9	3	233 237 243	Lu2553F	CAACCCTCCAAAACACATCC	60.2	50	19+20
						Lu2553R	TGGGAGAAATTATCCACTACGC	60.3	45.5	22
Lu2554	1	p3	(AAG)7	2	198 200	Lu2554F	TCGTCGGCTCAGTAATGTTG	59.9	50	19+20
						Lu2554R	AGAAGCAGCAGCTGGAGAAG	60	55	20
Lu2555	1	p3	(AAG)9	3	201 213 216	Lu2555F	TCCCGCTTTTAATGGTGTTC	59.9	45	19+20
						Lu2555R	AATTGGAAGCTCGATTCACG	60.2	45	20
Lu2556	1	p3	(ATA)12	3	307 316 319	Lu2556F	TTTCAAAAAGGCTCCGACTC	59.4	45	19+20
						Lu2556R	TTTTGCCCAAATTGTAAGTACG	59.1	36.4	22
Lu2557	1	p3	(ATT)10	6	278 302 308 311 338 344	Lu2557F	AGGTTGTCGTGGAAAGTTGG	60	50	19+20
						Lu2557R	AAAGCTAGCTACCGCACCAC	59.5	55	20
Lu2560	1	p2	(GA)11	2	null 238	Lu2560F	CGTGGCTACTAGCAATGTGG	59.4	55	19+20
						Lu2560R	TCCTCATGTTCATTGCTTGC	59.8	45	20
Lu2561	1	p3	(GAA)6	3	331 334 337	Lu2561F	GCTGAGGAGTTGGGTGTAGC	59.9	60	19+20
						Lu2561R	TCTTCCTTGGCTCTTCTTCG	59.7	50	20
Lu2563	1	p3	(GGA)8	2	null 395	Lu2563F	GTTCCGGTGAAGTGAGAAGG	59.7	55	19+20
						Lu2563R	AGAAGAAAAACCAAAGCACAGC	59.9	40.9	22
Lu2564	1	p2	(TA)10	2	255 257	Lu2564F	TTTCAGCTTCGATTGAGACG	59.1	45	19+20
						Lu2564R	ATCCGTCGAGGTAACAGTCC	59	55	20
Lu2565	1	p2	(TA)14	4	210 222 226 228	Lu2565F	TTTGGGCTTCTACTTTCTCCTG	59.9	45.5	19+22
						Lu2565R	AACCAAGAGGCTTCATACGG	59.2	50	20
Lu2567	1	p3	(TAT)11	4	313 325 331 334	Lu2567F	AACAAGACCTTGGCTTGGAG	59.3	50	19+20
						Lu2567R	TCATCACCATCGAATGAACC	59.3	45	20
Lu2571	1	p4	(TCCT)6	2	190 194	Lu2571F	GGTTGATTCAGACGGTCCTC	59.5	55	19+20
						Lu2571R	TGTGATGAATTCTGCCAAGG	59.6	45	20
Lu2572	1	p3	(TCT)9	2	250 253	Lu2572F	TTGAGGAATCGGAGATTTGG	60	45	19+20
						Lu2572R	AGGAATGTTCATCGGAGCAG	60.2	50	20
Lu2574	1	p3	(TTC)7	2	237 243	Lu2574F	AAGAACGCCTCATGTCTTCC	59.3	50	19+20
						Lu2574R	CCAGCCTATTTCAGGATTCG	59.7	50	20
Lu2575	1	p3	(CTT)6	2	337 340	Lu2575F	GTTCGGCCATAATGTTCCAC	60.2	50	19+20
						Lu2575R	CACAGTCATGCGAACTCCAC	60.3	55	20
Lu2576	1	p3	(GAA)9	2	362 365	Lu2576F	TCAGGCGAAAGGTTGAATG	59.8	47.4	19+19
						Lu2576R	CAATTTCTGGGTTTCCATCC	59.2	45	20
Lu2578	1	p2	(AT)14	6	177 187 191 193 197 205	Lu2578F	TGAGGGTGATCTCCACCAC	59.4	57.9	19+19

						Lu2578R	ATCATGAGTTGGCGTTTGG	59.5	47.4	19
Lu2580	1	p2	(AT)9	4	240 242 256 258	Lu2580F	AAGGTCGCTACTATTCCAAAAGC	60.2	43.5	19+23
						Lu2580R	TCTTGGTTTGTCGGACTATCG	60.1	47.6	21
Lu2582	1	p2	(AT)9	3	331 333 335	Lu2582F	TTCCCCTCCATTTCTTCTCTC	59.6	47.6	19+21
						Lu2582R	GCTCCACCATCCTTTTGAAC	59.5	50	20
Lu2583	1	p3	(ACC)7	2	313 315	Lu2583F	ATCATCTTCCTGCACCAACC	59.9	50	19+20
						Lu2583R	GAGGAGGGAGCGTCTAAAGG	60.3	60	20
Lu2587	1	p4	(CTAG)5	2	301 306	Lu2587F	TCGTACTGAACTGTTCGAAAACC	60.6	43.5	19+23
						Lu2587R	GGCCAGTAGTGTGGAGATGG	60.5	60	20
Lu2588	1	p2	(TA)11	2	328 338	Lu2588F	CGGGTCCGAGACACTAAATC	59.5	55	19+20
						Lu2588R	GGGGAGAAATGAATTGAGTTGA	60.3	40.9	22
Lu2589	1	p2	(AT)21	9	210 224 228 230 232 234 236 238 244	Lu2589F	CTGTGCAGTGTTTGTGAAAATG	59.3	40.9	19+22
						Lu2589R	CAGCCAATGGTTGAATGATG	59.9	45	20
Lu2592	1	p3	(AAT)6	2	292 295	Lu2592F	CAATTTGGCCACTCGTATTTG	60.4	42.9	19+21
						Lu2592R	TGCACACTGAAATATGAGCCTAAC	60.5	41.7	24
Lu2593	1	p2	(AG)14	2	362 364	Lu2593F	CTCTTCCGCTTCTATCAAATCC	59.4	45.5	19+22
						Lu2593R	CATGATGCCTTTCATGTTGG	59.9	45	20
Lu2597	1	p2	(TA)13	3	283 285 287	Lu2597F	CTTTCCCACCATAATAATAATACCC	58.2	36	19+25
						Lu2597R	GATTTGGAAAAATAAGCATGTCC	58.9	34.8	23
Lu2599	1	p3	(ACC)7	2	224 233	Lu2599F	AAGTAGCAATACATTGTCAAAGTCTCA	59.7	33.3	19+27
						Lu2599R	CCCTTCTCGATCACTTCCAG	59.8	55	20
Lu2600	1	p3	(ATC)8	2	357 363	Lu2600F	AGGTAAAAGTGGGCATGTCG	60	50	19+20
						Lu2600R	CCAAATTCCTCTTGCTTTGC	59.8	45	20
Lu2601	1	p2	(TA)10	2	348 350	Lu2601F	GCTTCTGCTTCGATTCATCC	59.9	50	19+20
						Lu2601R	CTTCATCAATGGTTTCCAAGC	59.6	42.9	21
Lu2602	1	p3	(AAG)7	2	288 291	Lu2602F	ATAGCCCTCGGAGAAAAAGG	59.7	50	19+20
						Lu2602R	TTGTAGCTGAACCAACCAACC	60	47.6	21
Lu2603	1	p3	(AGA)6	2	190 193	Lu2603F	GAGCTTGCTAAGGTTGTTAAAAGG	59.9	41.7	19+24
						Lu2603R	ACACTTTAACGTACGGGTGAGG	60.3	50	22
Lu2604	1	p2	(AT)10	2	273 275	Lu2604F	CCAGCTTTCATCCCTACTGG	59.7	55	19+20
						Lu2604R	GGGCAATTGAACGGATAGAC	59.4	50	20
Lu2605	1	p2	(AT)9	3	259 265 267	Lu2605F	GTGAAGCTCGACACTTCTGC	58.8	55	19+20
						Lu2605R	GCGACTCATACCGTACATGG	59	55	20
Lu2607	1	p2	(CT)10	3	232 236 238	Lu2607F	AAAAGCATCAACCACACACG	59.6	45	19+20
						Lu2607R	TTCTTTTCCCGCTGATGG	59.7	50	18
Lu2608	1	p3	(CTC)8	2	233 257	Lu2608F	GTGGAGCGATTTTCTGATCC	59.6	50	19+20
						Lu2608R	CGAGAATGGAGGAGGTTCC	59.6	57.9	19
Lu2609	1	p3	(CTC)8	2	301 307	Lu2609F	TGCTTTCATTATGCCCTTCC	60	45	19+20
						Lu2609R	GCCATGAAAAAGTTTGCTCTG	59.9	42.9	21
Lu2612	1	p2	(TA)14	7	283 285 289 295 297 299 301	Lu2612F	ATCATCGAATCAGGCCAAAC	59.9	45	19+20
						Lu2612R	TGGTGAACGTACATACATTACACATC	60	38.5	26
Lu2613	1	p2	(TC)12	2	400 402	Lu2613F	TGAGGGGAAGAACAGAATGG	60	50	19+20

						Lu2613R	GCACAGACGACTAGTACCGAGA	59.6	54.5	22
Lu2614	1	p3	(TTC)7	4	368 371 374 377	Lu2614F	CTCCGCCTATTGTTCTTTTCC	60.1	47.6	19+21
						Lu2614R	TCCCCCTACATTTCCTCCTC	60.3	55	20
Lu2617	1	p3	(GGT)7	2	198 201	Lu2617F	AACTGCACTTCTCCTTCTAGGG	59.1	50	19+22
						Lu2617R	GAAACGATTATCCGGACCAG	59.4	50	20
Lu2618	1	p2	(TA)13	3	198 200 202	Lu2618F	GTTTTGCCATGCTGACAGTG	60.3	50	19+20
						Lu2618R	AATATCTCCCCATCAGCCATC	60.1	47.6	21
Lu2621	1	p2	(CT)9	2	291 293	Lu2621F	GCTGGCAATAGCGAACTAGC	60.1	55	19+20
						Lu2621R	CATGGGTGGTGTGGTTGTT	60.1	52.6	19
Lu2622	1	p2	(GA)15	2	320 324	Lu2622F	TGCACCTATTTCCCTTCCAC	59.9	50	19+20
						Lu2622R	GAGAATTCCAGTGAATCGCTAGT	58.9	43.5	23
Lu2624	1	p3	(CTT)6	3	332 341 344	Lu2624F	CGTAGGGACATTGGAAGGAG	59.5	55	19+20
						Lu2624R	GAGCGGGATAAGGAAAAAGG	60	50	20
Lu2625	1	p2	(GA)10	3	350 358 366	Lu2625F	AGCAGAGAAACCGATGAACC	59.3	50	19+20
						Lu2625R	TCCGGAGAGGGAACTAACAC	59.1	55	20
Lu2626	1	p2	(AG)10	2	245 259	Lu2626F	CCACCGTCCATGTTCTTTG	59.9	52.6	19+19
						Lu2626R	TGACCTTTACGTTGGGAAGG	60	50	20
Lu2628	1	p3	(ATA)11	2	307 322	Lu2628F	TGGTCATGGTGTGGAATCAC	60.2	50	19+20
						Lu2628R	TGGACGCTAATAACCTCAAAGC	60.6	45.5	22
Lu2631	1	p5	(TCATG)5	2	284 289	Lu2631F	CGGGACAACATTCATGACC	59.8	52.6	19+19
						Lu2631R	TTTCGTTGCTGATTGTGAGC	60	45	20
Lu2633	1	p2	(AT)18	8	233 237 239 241 243 245 247 249	Lu2633F	CATGAATTAGCTCGGGTTCG	60.6	50	19+20
						Lu2633R	ACCCCATGATGATTGGTGAG	60.6	50	20
Lu2635	1	p3	(TCT)12	3	326 332 335	Lu2635F	TCCCTTCTTTTAATCTCCTCCTC	59.2	43.5	19+23
						Lu2635R	CCAGATGGGAAAATTGAAGC	59.5	45	20
Lu2637	1	p4	(TTTA)5	2	338 358	Lu2637F	CCGACTCTTCTTCCTTCTCG	59.2	55	19+20
						Lu2637R	CTGTTTCATTCCACGTTGTTG	59.1	42.9	21
Lu2638	1	p3	(CAT)6	2	277 292	Lu2638F	GACGGTGTTGTGCTTGAGG	60.3	57.9	19+19
						Lu2638R	CAGGCGCTGGTTATAGATGG	60.6	55	20
Lu2639	1	p3	(CCT)6	2	238 244	Lu2639F	GTGAGCGGAGCCCTAAAG	58.5	61.1	19+18
						Lu2639R	AGTCTGCCGTCCACCTTATC	59.2	55	20
Lu2644	1	p6	(GATGAG)5	2	336 342	Lu2644F	AGCTGAGATCCTTCCAGTCG	59.6	55	19+20
						Lu2644R	ACACTCAGCGGAGAAAGAGC	59.8	55	20
Lu2645	1	p2	(AT)13	3	209 217 223	Lu2645F	AAGGGTGGGAAATGTGAGG	59.8	52.6	19+19
						Lu2645R	CGTCTCGATTCCTGTTACTCG	59.9	52.4	21
Lu2648	1	p3	(CTT)10	2	344 356	Lu2648F	CCCTAATCAGACTGGGAAACC	59.8	52.4	19+21
						Lu2648R	TGGGTGTTTTAGGAGCTTGC	60.2	50	20
Lu2649	1	p3	(TCT)8	2	225 228	Lu2649F	TCGCTCCTAGGATGAAGCTC	59.7	55	19+20
						Lu2649R	GCTCGATTGACACAGTCTCG	59.6	55	20
Lu2651	1	p2	(AT)10	3	311 313 315	Lu2651F	TTAGCCACTTACATTATCAACCTACC	59	38.5	19+26
						Lu2651R	GAAAATAAGATCTTCCAAATTCATCC	59.6	30.8	26
Lu2652	1	p2	(AT)10	4	289 297 299 315	Lu2652F	GAACACATGTAGATGAAAAAGATGG	59	36	19+25

						Lu2652R	AAGCAAGCTCAGGATCAAGC	59.7	50	20
Lu2654	1	p2	(AT)18	5	239 245 247 249 253	Lu2654F	CTCAAACAGATTCATGCCAAAA	60.1	36.4	19+22
						Lu2654R	GAAACACCATCCATCCATCC	60	50	20
Lu2658	1	p2	(TA)10	4	342 350 354 356	Lu2658F	GCGAGGCTTCAGATACAAGG	60	55	19+20
						Lu2658R	CGTCTCCCATCGTAGAATGC	60.6	55	20
Lu2659	1	p3	(TCT)7	2	350 353	Lu2659F	GGCAGTCTTGCTTAAACTTGG	59	47.6	19+21
						Lu2659R	AAACGTCAAGTCGGAGAAGC	59.5	50	20
Lu2663	1	p3	(GAT)7	2	303 306	Lu2663F	TCGTCGTTAGGCTTCTCTCC	59.6	55	19+20
						Lu2663R	ACTGGATTATGCTTGAAGTCTGG	59.7	43.5	23
Lu2664	1	p3	(AAT)8	2	364 367	Lu2664F	TGGATCTTCCCACCAGTCTC	60	55	19+20
						Lu2664R	AGTGGCTGTATTGGGTTTGAG	59.1	47.6	21
Lu2668	1	p3	(CAA)8	3	329 332 338	Lu2668F	AGCCGGAGATCATCAACATC	60	50	19+20
						Lu2668R	TACACGTCGTCGTCACCTTC	59.8	55	20
Lu2669	1	p2	(CT)10	2	245 247	Lu2669F	TCTCATCCCAAGACCTACGG	60.1	55	19+20
						Lu2669R	GAATTGAAGCCTACAACCTTGC	60.1	45.5	22
Lu2672	1	p3	(GGA)6	2	346 349	Lu2672F	GATCCAAAGGCGAGAGAGG	59.9	57.9	19+19
						Lu2672R	CATGGGTATCCACCAGTTCC	60	55	20
Lu2673	1	p3	(GGA)6	2	354 357	Lu2673F	CGGGAATCTAGGGTTCTGG	59.5	57.9	19+19
						Lu2673R	TCCTGCATTCAACACGTAGG	59.7	50	20
Lu2676	1	p2	(TA)13	3	352 354 356	Lu2676F	TCTTCTCCCTCACTCATTCTCC	59.8	50	19+22
						Lu2676R	GTATATCGGTTCGGTTTTGTCC	59.6	45.5	22
Lu2677	1	p3	(TAT)11	3	320 323 326	Lu2677F	AATGCCTCCTCGTCATCATC	60	50	19+20
						Lu2677R	GTGTCAAACCAGCCTGGAG	59.2	57.9	19
Lu2679a	2	p3	(TTC)8	2	248 251	Lu2679F	GGTGGTTGGGTTTGAGTTTG	60.2	50	19+20
Lu2679b				3	null 231 240	Lu2679R	TCCACCACATCTCTCCAATG	59.5	50	20
Lu2681	1	p3	(CTT)6	2	197 209	Lu2681F	AGAATCCATACGTGGCAACC	59.8	50	19+20
						Lu2681R	TGGGAAATTTGGAAGAGAGG	59.1	45	20
Lu2684	1	p3	(AAG)11	3	307 310 319	Lu2684F	CGACATCTTCAAATCCTCGAC	59.7	47.6	19+21
						Lu2684R	CCATTTGCGGATCTGATTG	60	47.4	19
Lu2687	1	p3	(TTC)7	2	240 243	Lu2687F	TACAGAGAACAGGGGGTTGG	60	55	19+20
						Lu2687R	TCCATTAACGCGACTCTGC	60	52.6	19
Lu2689	1	p2	(AT)19	6	339 351 353 355 359 363	Lu2689F	TGATCATCCTTGGAACAAACC	59.8	42.9	19+21
						Lu2689R	AACAAAAAGTGGGGTTGCTG	60	45	20
Lu2693	1	p3	(TAT)6	2	312 339	Lu2693F	TTATAATATGCCGGTGGTTGG	59.6	42.9	19+21
						Lu2693R	GTGTTCAAAGAGACCGTCACC	59.6	52.4	21
Lu2694	1	p2	(TC)12	3	364 366 368	Lu2694F	ACCAAACATGGGCACTTCTC	60	50	19+20
						Lu2694R	TGCATGTGTGCAATTGTGAG	60.3	45	20
Lu2695	1	p2	(TA)9	3	303 337 339	Lu2695F	ATCACGTGGACACTTTGGAAC	59.9	47.6	19+21
						Lu2695R	CGCTTTTTATCACGAGAATCG	59.9	42.9	21
Lu2696	1	p2	(AG)13	3	318 322 330	Lu2696F	TGGAGCCTATGGATTGAAGTG	60.1	47.6	19+21
						Lu2696R	AACCGCCCCTCACCAC	60.5	68.8	16
Lu2697a	2	p2	(AT)10	2	308 310	Lu2697F	AAACGTGTAACCGGAAATGG	59.7	45	19+20

Lu2697b				3	330 334 338	Lu2697R	CCAAGAAAATGGTGGAAAGC	59.5	45	20
Lu2698	1	p3	(TAA)6	2	343 346	Lu2698F	TAAGGCAGGTCCCACATAGG	59.9	55	19+20
						Lu2698R	AAACAAAGCCGAAATCAACG	60.1	40	20
Lu2700	1	p2	(AT)19	5	336 338 342 360 362	Lu2700F	TTGGGACTCACCTATGTTAAACG	60.3	43.5	19+23
						Lu2700R	AACCAAACATCTGGCAAAGG	60	45	20
Lu2704	1	p2	(GA)9	3	280 286 288	Lu2704F	TCCCTGGCATGAAAGAAAAG	60.2	45	19+20
						Lu2704R	AAAGAGCAACCCCAACCTTC	60.5	50	20
Lu2706	1	p4	(GGTT)6	2	234 238	Lu2706F	AGAAGTCGCCATTGTTACGG	60.1	50	19+20
						Lu2706R	TCCAACCTTAACCGAAAACG	60	45	20
Lu2707	1	p2	(AC)9	2	325 327	Lu2707F	TAACAAAGCCAGCACACGAC	59.9	50	19+20
						Lu2707R	AATGTTAGGGGATCCGGAAG	60.1	50	20
Lu2712	1	p3	(AAT)6	4	326 344 347 350	Lu2712F	AGAGTGAACGAGCCAACAGG	60.4	55	19+20
						Lu2712R	TCCTCAAGGCAAAAGAATCG	60.3	45	20
Lu2714	1	p4	(AGCT)5	2	201 205	Lu2714F	AATGAAGCAAAGGCAAAAGC	59.5	40	19+20
						Lu2714R	CTCACCCATCCATATCTTACCC	59.6	50	22
Lu2718	1	p3	(ACT)8	2	355 364	Lu2718F	TTGCATGTTTTTGGAGAACG	59.7	40	19+20
						Lu2718R	ACAGGCCCTAGAGTTTAATTTCC	59.1	43.5	23
Lu2719	1	p3	(CGC)6	2	255 261	Lu2719F	TGAGGTGAAAACAATCCCATC	59.8	42.9	19+21
						Lu2719R	GGTTTCCAGGTTCTTCTCTGC	60.2	52.4	21
Lu2720	1	p3	(CTT)8	2	313 316	Lu2720F	GAAAGGCTGAAAGGCTGAAAC	60.4	47.6	19+21
						Lu2720R	TGAGGCGAAATTGGATTACC	59.9	45	20
Lu2724	1	p3	(GAA)6	2	302 308	Lu2724F	TTCTTGCTGATGTGCTCTGC	60.3	50	19+20
						Lu2724R	CCGCCGTAAATAACACATCC	60.2	50	20
Lu2725	1	p2	(AT)15	6	341 343 347 357 359 361	Lu2725F	ACCGGCTCACAGAAGTCAAC	60.3	55	19+20
						Lu2725R	AAAAATTGCCACGTTGAACC	59.8	40	20
Lu2727	1	p3	(TAC)7	3	359 362 365	Lu2727F	GGGGTTGGGTGTACATTGAC	60	55	19+20
						Lu2727R	AGCCATAGGTCCCCATTACC	60	55	20
Lu2728	1	p3	(TCT)8	2	336 342	Lu2728F	CGAACGAGTCGACAAAACAC	59.3	50	19+20
						Lu2728R	CCTGCTCCTGGAGTGAAGAC	60	60	20
Lu2729	1	p3	(AAT)13	2	264 270	Lu2729F	CCCCCTCTTCATTTTGCTTAG	60.1	47.6	19+21
						Lu2729R	TGAGCAGAGCTCCTATTCGTC	59.7	52.4	21
Lu2730	1	p2	(AG)11	3	257 271 275	Lu2730F	TCATGGTATGGGTTCAGACG	59.4	50	19+20
						Lu2730R	GATTGGTGGTGTGGTCAGTG	59.8	55	20
Lu2731	1	p2	(AG)11	5	367 369 377 379 381	Lu2731F	AGTTACAGGGGCGGAACAC	60	57.9	19+19
						Lu2731R	TGAGAACAACCCGGAAAGAC	60.1	50	20
Lu2732	1	p2	(AT)20	5	341 343 347 349 351	Lu2732F	TTTCTCAATTATTAACACACACACAC	57.4	30.8	19+26
						Lu2732R	TTTGCTAGGTTGGCAAAAAC	58	40	20
Lu2739	1	p2	(AT)15	5	290 292 294 296 298	Lu2739F	AAATTGCATAGATGGGATGACC	60	40.9	19+22
						Lu2739R	TGTCCTAGCGTGGAAAAAGG	60.2	50	20
Lu2740	1	p3	(TGC)7	2	222 225	Lu2740F	CAGTGTGCATGGCTTTTCC	60.3	52.6	19+19
						Lu2740R	GGCGGAATTCCTTTTCACTAC	60	47.6	21
Lu2741	1	p3	(CTT)7	2	188 191	Lu2741F	AGCAGCAGGAAATTGACCAC	60.3	50	19+20

						Lu2741R	CTTTCCCCCACCAACTTTTC	60.7	50	20
Lu2745	1	p3	(GCG)6	2	282 288	Lu2745F	AGCGTAGGAAGCCATGAGG	60.4	57.9	19+19
						Lu2745R	CCCCTTCACAACTCCATCC	60.3	57.9	19
Lu2746	1	p2	(TA)9	2	406 408	Lu2746F	AGGTCACGACCACTCATCATC	60	52.4	19+21
						Lu2746R	CGTGCGAAACAAAATGAAAC	59.2	40	20
Lu2751	1	p3	(GAA)11	4	282 291 294 297	Lu2751F	AGGGGAAAGCTGAGTTGAGAG	60	52.4	19+21
						Lu2751R	GACATGGACATCCCAAGGAC	60.2	55	20
Lu2752	1	p2	(CT)10	2	212 214	Lu2752F	ATTCGTGATCGGCAATAACC	59.8	45	19+20
						Lu2752R	GGAGCTGGGAAATGATCAAG	59.6	50	20
Lu2756	1	p3	(GAA)7	2	null 301	Lu2756F	CGTCGTCATTGTCAATCTGC	60.3	50	19+20
						Lu2756R	TCAAGCACGACATTCTTTCG	60	45	20
Lu2757	1	p4	(CTTT)5	2	303 307	Lu2757F	AAACACATACTGCCGCAGAG	59	50	19+20
						Lu2757R	CCGTTAGGCTAGGAACATGC	59.7	55	20
Lu2758	1	p3	(GAT)8	3	225 228 231	Lu2758F	GTGGCTCAAAACAACTCTACCC	60	50	19+22
						Lu2758R	TTTCTCTTCTCCTGCTTGTCG	59.7	47.6	21
Lu2764	1	p2	(AT)13	3	203 205 209	Lu2764F	CATTACGTTGGTGGATTCTCG	60.4	47.6	19+21
						Lu2764R	CCCAGCTAGCATGAATACCC	59.6	55	20
Lu2767	1	p4	(GAAA)9	2	242 254	Lu2767F	CAGCTGGGGGTGAATGAAT	60.9	52.6	19+19
						Lu2767R	AGTTGCTAGGCGATGGAGAA	60	50	20
Lu2770	1	p3	(ATC)8	2	282 288	Lu2770F	CGACGACAATGAACAAATGC	60.1	45	19+20
						Lu2770R	GTAGCCGTTGGGTTCTTGG	60.5	57.9	19
Lu2771	1	p3	(GAA)8	3	347 350 353	Lu2771F	AGATGAAGGGGGAAATGAGG	60.3	50	19+20
						Lu2771R	GACCAACCAAACCAAAATCG	60.2	45	20
Lu2773	1	p2	(TA)10	4	347 351 359 363	Lu2773F	GATACGTGGCAAAGCAAAGAG	59.9	47.6	19+21
						Lu2773R	CCATGCACGCACTATGTTG	59.7	52.6	19
Lu2774	1	p3	(AGA)8	2	357 360	Lu2774F	AGGCTGAGGTTTCAAGTTGC	59.5	50	19+20
						Lu2774R	TGGGTTCACCTTTTCTTTCG	60.1	45	20
Lu2775a	2	p3	(TCT)6	2	273 276	Lu2775F	TCCTCTCAGCTGGCTTCTTC	59.8	55	19+20
Lu2775b				2	164 167	Lu2775R	GAACAGTGGCATTCCCGTAG	60.5	55	20
Lu2776	1	p3	(AGT)7	3	296 302 305	Lu2776F	CACCAAAAACTGGGCTTACC	59.5	50	19+20
						Lu2776R	TGCAACTGCAAGATAGAAAAGG	59.5	40.9	22
Lu2777a	2	c	(AG)19aaaactcacttgatctgtgaagaatccaacaggggaaacagagcaataattgatctgaacacagaaaaggaaaagggagaagatggag(AAC)7	3	401 403 405	Lu2777F	CAAGGCGGATTTACAGAAGG	59.7	50	19+20
Lu2777b				3	349 351 353	Lu2777R	GTGGTTAGGGATATTTGTGATTCC	59.9	41.7	24
Lu2778	1	p2	(AT)21	6	298 300 302 304 316 321	Lu2778F	TCCCCAATGCTCTGGTTATC	59.9	50	19+20
						Lu2778R	AGATGTCAATGAGGCATCACC	59.9	47.6	21
Lu2779	1	p3	(ATA)13	4	263 266 269 275	Lu2779F	CAGAGGCCAGTTCATTCCAT	60.1	50	19+20
						Lu2779R	TACTCTCCTGCCGCCTCTAA	60.1	55	20
Lu2780	1	p5	(CGTAG)6	2	187 193	Lu2780F	AATGGTTCTTCGTCGGTTTG	60	45	19+20
						Lu2780R	AAGGAAATTGCTGCATACGG	60.1	45	20

Lu2782	1	p3	(CTT)16	3	257 281 284	Lu2782F	CTCCCTCGTTTTCTTCGTTG	59.8	50	19+20
						Lu2782R	CAGGAGAAGTGGGAATTTGC	59.7	50	20
Lu2783	1	p2	(GA)9	2	359 361	Lu2783F	CGCTTCCTCTCTCTCTCCAC	59.3	60	19+20
						Lu2783R	CCACCCACCCTACATACGAC	60.1	60	20
Lu2784	1	p5	(GAACA)5	2	323 333	Lu2784F	TTGTAGAGTCGTTAGCAGATTGATG	59.9	40	19+25
						Lu2784R	GAGTGGATGTGGGTTGCAC	60	57.9	19
Lu2787	1	p3	(TTA)18	3	292 295 298	Lu2787F	GTTGGGTGGAAACAATTTGG	60.1	45	19+20
						Lu2787R	GCAGCAGTAAGCAGCAAGC	60.1	57.9	19
Lu2793a	2	p2	(TA)9	2	null 353	Lu2793F	CGACAGCATTGAAAGACAGC	59.6	50	19+20
Lu2793b				2	310 345	Lu2793R	TTTCTGTGCTTTCCCTACCC	59.2	50	20
Lu2794	1	p3	(AAG)7	3	212 215 218	Lu2794F	TTGGTGTTGTTGCTGTTTCC	59.6	45	19+20
						Lu2794R	GCAGATCCAATCCAACACG	60.1	52.6	19
Lu2795	1	p2	(AT)15	5	341 343 347 351 353	Lu2795F	TCCCTTTCTCTCCGTGGAC	60.2	57.9	19+19
						Lu2795R	CATGGATAGGGACCAACGAC	60.2	55	20
Lu2796	1	p3	(ATA)15	5	187 190 199 202 208	Lu2796F	GGCAACAAGAAGGCAAGAAC	59.9	50	19+20
						Lu2796R	GGGTGGCCAACACATCTTAC	60.2	55	20
Lu2799	1	p4	(GTAC)5	2	194 198	Lu2799F	CACTTTCGATCATCTTCTCTTGG	60.2	43.5	19+23
						Lu2799R	GACGTCAACTGGTTGTGTCG	60.2	55	20
Lu2800	1	p3	(TCG)6	3	212 215 218	Lu2800F	AGCAGCAATCATCGGTAAGG	60.2	50	19+20
						Lu2800R	GCTCACGGGCATCTAATCC	60.6	57.9	19
Lu2801	1	p3	(GAA)10	2	252 255	Lu2801F	CGTAATCATCATCTCTCAGTAGGG	59.2	45.8	19+24
						Lu2801R	CCTCTTGGATTCATCTTTCTCC	59.2	45.5	22
Lu2802	1	p3	(AAG)7	2	316 319	Lu2802F	CCCATGCCACATAATGTATCC	59.9	47.6	19+21
						Lu2802R	TGCATCTGGAACTGAACAGC	60	50	20
Lu2803	1	p2	(AT)13	4	344 346 352 356	Lu2803F	TGCAGCTCGTTTTTCTTCG	60.3	47.4	19+19
						Lu2803R	ACGGTTCGTTTTTACTTCAAGC	59.7	40.9	22
Lu2807	1	p3	(TAT)10	3	221 224 227	Lu2807F	TGATTCAGTGGCTTGTCACC	59.7	50	19+20
						Lu2807R	AATTAATGGGGCTGGCTAGG	60.3	50	20
Lu2808	1	p3	(TTG)6	2	266 269	Lu2808F	ATCTCAAGGTTCGGATGCAG	60.2	50	19+20
						Lu2808R	AAGCACTTGCTTCTCCCTCTC	60.1	52.4	21
Lu2809a	2	p2	(AG)10	2	347 349	Lu2809F	GGTCAAGGCTGAGAAATTGG	59.7	50	19+20
Lu2809b				5	374 372 370 352 350	Lu2809R	ACGCATCCTTAGTGGACCTG	60.1	55	20
Lu2810	1	p2	(AT)15	6	259 256 254 242 240 238	Lu2810F	ACGATGAAACCACAAGACCAG	60	47.6	19+21
						Lu2810R	GACGTGTAAAACGGGTTTGG	60.3	50	20
Lu2812	1	p3	(CAG)13	3	322 303 297	Lu2812F	GGGATTATTGGGGTTGTGG	59.9	52.6	19+19
						Lu2812R	AAGGCTCTTTGCTTCCTTCC	60	50	20
Lu2813	1	p3	(AAT)8	2	213 207	Lu2813F	GAGACCCCTAGTCGAACTGC	58.9	60	19+20
						Lu2813R	TTTGCTTCCGTTATCTCTTGC	59.5	42.9	21
Lu2814	1	p3	(GGA)6	2	208 202	Lu2814F	TCTGCACCGTCATAATCACC	59.5	50	19+20
						Lu2814R	GGGAGAGGGAGAAGAAGAGC	59.5	60	20
Lu2815	1	p3	(TCA)7	2	null 296	Lu2815F	ACGTGACTCCTGGAGATTGC	60.3	55	19+20
						Lu2815R	TACCCGCGTTTTTCACTTTC	60.1	45	20

Lu2819	1	p2	(TA)10	2	321 323	Lu2819F	GCCTCTAAAGCTAATTGCATAGG	58.7	43.5	19+23
						Lu2819R	AGGGACAATGAACAGTCCTACAC	59.4	47.8	23
Lu2820	1	p3	(TCT)6	2	351 354	Lu2820F	AGTCGTTTTCTAATCGGCCTAA	59.3	40.9	19+22
						Lu2820R	ATCGAACGGAGAATGTACGG	60	50	20
Lu2822	1	p3	(TAT)11	2	295 298	Lu2822F	AATGAGGCCACTCCATAACG	60	50	19+20
						Lu2822R	TTTGGATGTGAGCTTCCTGTT	59.7	42.9	21
Lu2823	1	p3	(TTG)6	2	287 293	Lu2823F	AAATTGGTGGAATCCGAATG	59.6	40	19+20
						Lu2823R	AAAAGAACGCGACGATTAGC	59.5	45	20
Lu2824	1	p3	(AAG)6	2	204 207	Lu2824F	CTCCCAAGGTGAAGAAATCG	59.7	50	19+20
						Lu2824R	AGAGCTGAAGCAATCTGAACG	59.8	47.6	21
Lu2825a	2	p2	(AT)16	2	311 313	Lu2825F	CTTTGCTTGCATTTGAGAACC	59.9	42.9	19+21
Lu2825b				6	395 397 409 411 413 415	Lu2825R	CTGAGAACGCAAGCTATCAGG	60.2	52.4	21
Lu2827	1	p3	(GAA)6	2	336 348	Lu2827F	TGCCCCATATCTCAGAGGTC	60	55	19+20
						Lu2827R	AGAATGCCACCATTGCTCTC	60.2	50	20
Lu2828	1	c	(GAA)7ggaaataattaagtacgaaggatgac ggtcaga(GCT)7	2	204 207	Lu2828F	TTGCATGGCTGTTGAATAGG	59.7	45	19+20
						Lu2828R	TTGGGCCACAACAGATCC	60.5	55.6	18
Lu2829a	2	p4	(GAAG)5	2	322 326	Lu2829F	GAAAAAGCGGTGACAAGGAG	59.9	50	19+20
Lu2829b				2	305 309	Lu2829R	TGGTGCAGGTCTCACTTCTG	60	55	20
Lu2832	1	p2	(TA)25	7	421 427 437 457 459 477 479	Lu2832F	GAACCCGATTTAACAGTGTGG	59.3	47.6	19+21
						Lu2832R	CGATTTGAGATTTTGAATCAACC	59.8	34.8	23
Lu2833	1	p3	(TAA)6	2	275 284	Lu2833F	GAGAAATGGAGGAGACGAACC	60.1	52.4	19+21
						Lu2833R	TCGAGTCGAGGTGAACCTATG	60.3	52.4	21
Lu2835	1	p3	(TCT)6	2	222 225	Lu2835F	ACGAGGAATTGGAGAATGACC	60.3	47.6	19+21
						Lu2835R	TGAGGAGGATTTGAAGTGACG	60.2	47.6	21
Lu2836	1	p3	(TTC)6	2	192 195	Lu2836F	ATCTTGCAAGGAACCTGACG	60.3	50	19+20
						Lu2836R	TCTCCTTCTCCGTTGATTCG	60.3	50	20
Lu2837a	2	p2	(AG)12	2	null 355	Lu2837F	TGATGTGACTCTGCCTCTGG	60	55	19+20
Lu2837b				2	null 353	Lu2837R	GTCTGTAGGTGCTGCTGACG	59.6	60	20
Lu2838	1	p3	(CCG)6	2	null 254	Lu2838F	CTGTCCGACGCCCTAATATC	59.6	55	19+20
						Lu2838R	CATGCCGACCTAGTGTTTACC	59.5	52.4	21
Lu2839	1	c	(ATA)11ctaatattaaaagacataaagaaatta taacataaattcattaatttatcaactgctaccgt(A TA)6	2	353 358	Lu2839F	TTCATTAATTTATCAACTGCTACCG	58.7	32	19+25
						Lu2839R	TGCCACCATCATTTTATCAAG	58.5	38.1	21
Lu2840	1	p3	(ATG)6	2	300 324	Lu2840F	TGCAGGCAGTTATGATCAGC	60	50	19+20
						Lu2840R	TCATCCTGCTCCACTGTGTC	59.8	55	20
Lu2843	1	p2	(TC)11	2	null 346	Lu2843F	ACCTGGTTTTCTTGTCTGTTCC	59.5	45.5	19+22
						Lu2843R	TCCAAACATCCACAATCGTC	59.3	45	20
Lu2847	1	p3	(TGA)6	2	355 358	Lu2847F	GCACAAAGAGGGAAAATTGG	59.5	45	19+20
						Lu2847R	GTTCGGACAAGGGACAAGAG	59.7	55	20
Lu2848	1	p3	(TTC)6	2	256 257	Lu2848F	GACCAGCTTCCTCTGTTTCG	60	55	19+20

						Lu2848R	CATCATCTCCGAGAAACTTGC	59.8	47.6	21
Lu2850	1	p6	(TTGGGA)7	3	258 270 276	Lu2850F	TAGCTCAGCTCCGAAAATGG	60.5	50	19+20
						Lu2850R	GGCTAATTGAGGGACCAAATC	59.8	47.6	21
Lu2851a	2	p4	(ACAA)5	2	491 521	Lu2851F	GCCTACAATAAGAAGCCAAATCC	60.3	43.5	19+23
Lu2851b				2	301 326	Lu2851R	GGCTTTTCCCACATGATCC	60.3	52.6	19
Lu2853	1	p2	(AT)14	5	283 291 293 297 305	Lu2853F	TCATTCCACTGTTCTCCAAGC	60.2	47.6	19+21
						Lu2853R	GAAAATTTGCATGCCTGGTC	60.5	45	20
Lu2855	1	p4	(CAGT)5	2	360 364	Lu2855F	ATGAAGGTCCCGTCACTGAG	60.1	55	19+20
						Lu2855R	AATGAGTGCTCTGGAAACGTG	60.3	47.6	21
Lu2857	1	p2	(GA)11	2	252 254	Lu2857F	TCCTCATCCTCATCCTCCTG	60.2	55	19+20
						Lu2857R	GCTTGCTGGAGGAATTTGAC	59.8	50	20
Lu2858	1	p3	(GAG)6	2	186 195	Lu2858F	CAGGGATGTCTGCTAGAGAGG	59.1	57.1	19+21
						Lu2858R	ATCCCACCATCACCATCTTC	59.6	50	20
Lu2859	1	p3	(GAT)9	2	267 273	Lu2859F	GGGGGAAGAAGAAGAAGACG	60.2	55	19+20
						Lu2859R	TTTGGTCTTGTATCAGCAGTAAGC	59.9	41.7	24
Lu2861	1	c	(TCCAG)5tcccccaccattccctccctcccaa accctctctagagcaatcaatt(GCA)7gagag atatcatcaaagagaaagacaaagaaaagcgt cagagaaagaagccctatctt(CAG)7	3	313 328 333	Lu2861F	ACCCCTCTCTTCCTTTTTGC	59.7	50	19+20
						Lu2861R	GAGGGGGAGTAACAGTTGAGG	60	57.1	21
Lu2862	1	p3	(AAT)7	2	201 207	Lu2862F	ATTAAATTGAGGCACCACCAC	58.8	42.9	19+21
						Lu2862R	CAGCCGCAAACATTAACTTG	59.4	45	20
Lu2863	1	p2	(AT)18	9	353 357 359 365 367 373 375 379 381	Lu2863F	CTTTGCATGTGCTTAGCTTCC	60	47.6	19+21
						Lu2863R	GTTGACAATTAAATCTGACCAGACC	60.2	40	25
Lu2865	1	p4	(TATT)7	2	306 302	Lu2865F	GCTCGAGTTCGACCAGTAGG	60	60	19+20
						Lu2865R	CACATGCATTTCCAAGTTGC	60.1	45	20
Lu2866	1	p3	(TCT)7	2	357 360	Lu2866F	TGCTGGACAGTAGAAGATGTTAGC	60	45.8	19+24
						Lu2866R	GTCCTGCTTTATGCCTTTGC	59.9	50	20
Lu2867	1	p3	(TGG)6	3	306 309 318	Lu2867F	CCGAGGTAGCAAGTGACTCC	59.9	60	19+20
						Lu2867R	CCCTCTGCATCAACCTCTTC	59.8	55	20
Lu2868	1	p6	(TTTTTG)5	2	312 314	Lu2868F	GGAACGAAATCTTGCTGGAC	59.7	50	19+20
						Lu2868R	CGGTGGTTTAAGCTCCATTG	60.5	50	20
Lu2870	1	c	(AGA)7g(GAA)6gag(GAA)7	2	280 317	Lu2870F	AGTCGGGACCCTTGAACTG	60.1	57.9	19+19
						Lu2870R	ATTCGGCAGCTCTGTTGTTC	60.4	50	20
Lu2871	1	p3	(AGC)6	2	341 344	Lu2871F	TCTTGGACCATCATTTTCACC	59.8	42.9	19+21
						Lu2871R	ATGACTGCGCAAAATGTCC	59.7	47.4	19
Lu2873	1	p2	(CT)13	3	304 306 312	Lu2873F	ACAGCGCACACCTACTTGG	59.9	57.9	19+19
						Lu2873R	TTCCGATTCTGTTGCAAGG	59.8	47.4	19
Lu2874	1	p3	(CTT)8	2	256 259	Lu2874F	TAACCTGGAGGAGGATGGTG	59.9	55	19+20
						Lu2874R	ATAAACCAGCCAACGGAATG	59.8	45	20
Lu2875	1	p3	(TAA)10	2	214 217	Lu2875F	TGTTGAGACTTGAGAGCTTCCTC	60.2	47.8	19+23

						Lu2875R	TACCGGTGGCTTTCCAGTAG	60.1	55	20
Lu2878	1	p5	(ATTAA)5	2	308 313	Lu2878F	TATTGGCCGTTGGATCTTTC	59.9	45	19+20
						Lu2878R	ACAAAGCGGATAGCTTGTCG	60.4	50	20
Lu2880	1	p3	(GCT)8	2	387 396	Lu2880F	CCACAGCCATTGTTTATTTGG	60.2	42.9	19+21
						Lu2880R	GCCATTGATGAGTGATGTCG	60.1	50	20
Lu2881	1	c	(TCT)12tcg(TCT)6	2	321 325	Lu2881F	AAGAAACTCGGTGGTGATGG	60	50	19+20
						Lu2881R	ATTTGGAGGAGCCACAAGG	60.1	52.6	19
Lu2883	1	p3	(CTC)7	3	203 206 209	Lu2883F	CGCTCACGTGATACCATACG	60.2	55	19+20
						Lu2883R	GATCCCGTCAAATCTATTCTGC	59.9	45.5	22
Lu2884	1	p3	(TAT)10	2	499 505	Lu2884F	CCCTTCATGAATCATTTTTCTCT	58.6	34.8	19+23
						Lu2884R	TGCTTGGTCTCTTGGCATTA	59.4	45	20
Lu2885	1	p4	(TTCT)9	2	277 285	Lu2885F	TAACATGTGGATGCCGAGAG	59.7	50	19+20
						Lu2885R	AGCCAATTAGGTTCCCCAAC	60.2	50	20
Lu2886	1	p3	(TCC)6	2	399 402	Lu2886F	TGCAGCTCAAATACGGACAG	60	50	19+20
						Lu2886R	GCCAACGAGATCAGCTAAGG	60	55	20
Lu2887	1	p2	(AT)10	2	300 320	Lu2887F	CGGGACGAACTTGAAAAGG	60.6	52.6	19+19
						Lu2887R	GAAGAAAAGGAAGAGCATCAGC	59.6	45.5	22
Lu2888	1	p3	(AAC)7	2	259 262	Lu2888F	TTATTGGCCTGCCTCTTAGC	59.5	50	19+20
						Lu2888R	TTGGAAGACGATGCAGTTTG	59.8	45	20
Lu2889	1	p2	(AG)11	2	297 299	Lu2889F	CTAAGCTCCCGCTGTTGTTC	60	55	19+20
						Lu2889R	TGCCTGCTATACACACAGTTCAC	60.2	47.8	23
Lu2890	1	p2	(TA)15	3	190 208 212	Lu2890F	TGTCATCATTATACCAACTTTGTGA	58.5	32	19+25
						Lu2890R	GATGTTTTTACAGAGGTATGTTTTCC	58.6	34.6	26
Lu2894	1	p3	(TGC)8	2	197 206	Lu2894F	TCGGAAATTGTGGGTAATACG	59.7	42.9	19+21
						Lu2894R	CCCTAAACCACACGAACACC	60.3	55	20
Lu2895a	2	c	(GAA)6gaggaaatagaattgcagagaagaa actgggggaaatggcttagaattgaagtgctgag tcgaagaagaaga(AGT)6	2	216 219	Lu2895F	TGGTGAGTTTACCTCGAGTCC	59.2	52.4	19+21
Lu2895b				2	206 212	Lu2895R	TTCATTCACATCTCCCTTTCAC	59	40.9	22
Lu2898	1	p2	(AT)10	2	213 215	Lu2898F	GAAAGCCCACGGTATTGACT	59.1	50	19+20
						Lu2898R	GTTGGAGTTGGCCCTATGAA	59.9	50	20
Lu2899	1	p2	(TA)10	3	null 274 280	Lu2899F	AAACGTTATCCGGCCAATC	59.8	47.4	19+19
						Lu2899R	AAGCCGTTTTTCTTTCAGAGAC	59.1	40.9	22
Lu2901	1	p2	(GA)17	4	232 234 236 244	Lu2901F	TGCTTCTGCAAATTAGGATCG	60.4	42.9	19+21
						Lu2901R	TGACAGCAGGACACTGAAGC	59.1	45	20
Lu2905	1	p4	(ATGT)6	2	322 326	Lu2905F	CACTACTAGAAATGCCCCAACC	59.9	50	19+22
						Lu2905R	AAGCTGCAATGTGGAGTGC	60	52.6	19
Lu2907	1	p2	(GA)11	2	288 290	Lu2907F	GGTTGAAGCAACCAAAAAGG	59.6	45	19+20
						Lu2907R	TGGCATGCTTTACTTGTACCC	60	47.6	21
Lu2908	1	p2	(AT)11	4	348 350 352 356	Lu2908F	TGCAGCACCAATATAATTCCAC	59.9	40.9	19+22
						Lu2908R	TCGGTGGTTAGTGTGATTGC	59.6	50	20
Lu2909	1	p3	(ATT)9	2	347 353	Lu2909F	AGGGCCGATTTGTTAACTTG	59.1	45	19+20

						Lu2909R	CCTTCACCTAGCTCACTTTACTTG	58.7	45.8	24
Lu2911	1	p2	(CA)10	2	277 279	Lu2911F	CGATGCTTGTTTTGAAGTGC	59.5	45	19+20
						Lu2911R	CTGACACGAATGGAACTTGC	59.3	50	20
Lu2912	1	p4	(AGAA)8	2	316 332	Lu2912F	CAAAGGGACAAAACAGAAGAGG	60.1	45.5	19+22
						Lu2912R	TTCCCTTTCCCATCTCTGG	60	52.6	19
Lu2913	1	p3	(CTT)7	3	215 218 224	Lu2913F	GCTCTGCTCGCTGGTTAGAG	60.4	60	19+20
						Lu2913R	GAGAGCAGCTGCAGTGATTG	59.9	55	20
Lu2914	1	p3	(TCT)6	2	223 226	Lu2914F	TTCGGCAGACAGTTCTCCTC	60.5	55	19+20
						Lu2914R	TGGTTGCTTAGGGAATGATTG	59.9	42.9	21
Lu2916	1	p3	(GAA)8	2	207 213	Lu2916F	CTGTGAACATTCCCCAACG	59.9	52.6	19+19
						Lu2916R	CAAGCAACCCACGTTTCC	60.1	55.6	18
Lu2917a	2	p3	(GAA)9	3	200 209 215	Lu2917F	TCGTCGAGAATCATGTCTTCC	60.2	47.6	19+21
Lu2917b				2	191 194	Lu2917R	TCACTTCCCAATCCAAAATACC	60.1	40.9	22
Lu2918	1	p3	(TCT)6	3	285 291 294	Lu2918F	ACGTACCAGACAGGCAGACC	60.2	60	19+20
						Lu2918R	GGCTAGAAAGAGGGCAAAATG	60.2	47.6	21
Lu2921	1	p3	(AAT)9	4	317 320 323 332	Lu2921F	CTGTGGCAGAAGATGCAGAG	59.7	55	19+20
						Lu2921R	TGAGCCACCAGTTTCATTTG	59.7	45	20
Lu2923	1	p2	(AT)12	5	219 227 229 235 237	Lu2923F	TTGCACCCGATACATATTCC	58.3	45	19+20
						Lu2923R	CTAGCCTTTCTTGGTTGAAGG	58.1	47.6	21
Lu2926	1	p2	(TA)21	3	205 225 235	Lu2926F	TGGATTTGGAGCAGTGATACC	59.9	47.6	19+21
						Lu2926R	GAGCCAGCTACTGCCGATAC	60	60	20
Lu2927	1	p3	(AGA)11	3	187 190 193	Lu2927F	ATTTGTTTATGGAGAAGAATCTGATG	59	30.8	19+26
						Lu2927R	TGTCAATATCTAGTGAGACAGACACG	59.8	42.3	26
Lu2928	1	p3	(AGA)6	2	196 199	Lu2928F	ACGGGTTTGTCTTGAAATGC	60	45	19+20
						Lu2928R	TTGGAGTATGTGTGGGGTTTC	59.7	47.6	21
Lu2929	1	p3	(AGA)8	2	230 233	Lu2929F	TCTTCCTTTACCGTGAAAAACG	60.5	40.9	19+22
						Lu2929R	CCTTGCATCGAGAAGTTTCC	59.8	50	20
Lu2930	1	p3	(GAG)6	2	302 308	Lu2930F	CTTCGAACCCAATTTTCTGC	59.7	45	19+20
						Lu2930R	TCCTCTCTCACTGTTTCTCTCAAC	59.2	45.8	24
Lu2931	1	p2	(AG)9	2	222 224	Lu2931F	CCAAACATGAAACCAGAGAGC	59.7	47.6	19+21
						Lu2931R	CTTGGATCTTCCGGTACTGC	59.7	55	20
Lu2932a	2	p5	(AATAG)5	2	null 338	Lu2932F	TATTGCAAACGGACAAGTGC	59.7	45	19+20
Lu2932b				2	null 318	Lu2932R	CGTATGACATGCAACCAAGC	60.1	50	20
Lu2935	1	p4	(AATT)6	2	187 195	Lu2935F	GACGTGAAGAAGTGGGAAGC	59.9	55	19+20
						Lu2935R	GGTGAATTGTCAGCAAATGG	59	45	20
Lu2936	1	p2	(AG)12	2	353 355	Lu2936F	GGTCAAGGCTGAGAAATTGG	59.7	50	19+20
						Lu2936R	TGCCAAACACGTCCTTAGTG	59.8	50	20
Lu2937	1	p3	(GAT)6	2	238 244	Lu2937F	CCAATGTCTGCATTTCTTGC	59.3	45	19+20
						Lu2937R	ACCGATTCGAACCAGAAGG	60.1	52.6	19
Lu2938	1	p3	(GGT)6	2	336 339	Lu2938F	CCGAAGTCAGCGAGAAAAAG	60.1	50	19+20
						Lu2938R	CATTTGTTGGGCGGTTTATC	60.2	45	20
Lu2939a	2	p3	(TGT)6	2	311 314	Lu2939F	ATTCGTGTTGGTCACTGAAGG	60	47.6	19+21

Lu2939b				2	288 291	Lu2939R	AGCAGCAACAAGGTCAACG	60	52.6	19
Lu2940	1	p3	(AAG)7	2	261 282	Lu2940F	AGGTGGCTGATTGAAAAATCC	60.3	42.9	19+21
						Lu2940R	CAAAATTACTTCAGGGGAATGC	59.8	40.9	22
Lu2942	1	p2	(CT)9	2	191 195	Lu2942F	AAGGAAAGGGAAGGCTCATC	59.7	50	19+20
						Lu2942R	TCGTGGAATTAATTGCGTCTC	60.1	42.9	21
Lu2943	1	p3	(GAA)6	4	194 209 218 224	Lu2943F	CCGATGATCATGCCCTAAAG	60.4	50	19+20
						Lu2943R	AGAGATCGGTGATGGGTTTG	59.9	50	20
Lu2944	1	p2	(TA)12	4	259 261 263 267	Lu2944F	TTTTCACGTCGACAAAATGC	59.7	40	19+20
						Lu2944R	CGATATGGACAACCCAACG	59.8	52.6	19
Lu2945	1	p2	(TA)9	2	317 319	Lu2945F	CAGAAAGCCGACAGAAAAGG	60	50	19+20
						Lu2945R	GCCATCTTCAGTCTTGTGTATCC	60	47.8	23
Lu2947	1	p3	(TTC)9	2	271 274	Lu2947F	GTTGCAGTTGTGCATTCTGG	60.3	50	19+20
						Lu2947R	TCTGCAGCTTCCAATTCCTC	60.5	50	20
Lu2948	1	p3	(AGT)10	2	284 287	Lu2948F	ACGCAGGACGTTCTTATTCAC	59.3	47.6	19+21
						Lu2948R	GAAGCTGAAGGGTGCAGAAC	60	55	20
Lu2949	1	p3	(GAA)6	2	272 275	Lu2949F	GAAAAACGGGAGGGAGAAAG	60	50	19+20
						Lu2949R	ATCACCGGCTTCATTTCATC	59.9	45	20
Lu2950	1	p2	(TA)13	5	316 324 338 344 358	Lu2950F	TGTGGGTGTACCGAAGTTCA	60	50	19+20
						Lu2950R	GCCACATCATCATGACCACT	59.4	50	20
Lu2951	1	p2	(TA)17	2	235 237	Lu2951F	TACCGTGGGAACTGGTAAGC	60	55	19+20
						Lu2951R	TGAGCCAATGCTCACAAAAC	59.8	45	20
Lu2955	1	p2	(AG)10	2	279 285	Lu2955F	TGTTCCCGCTCTCACTTCTC	60.5	55	19+20
						Lu2955R	TGATTACGCCGCCTCTATTC	60.2	50	20
Lu2956a	2	p3	(TTA)6	2	282 291	Lu2956F	AGCCAATGGAAATTCTGGTG	59.9	45	19+20
Lu2956b				2	229 232	Lu2956R	TGCTTTGCTACATGCGAAAC	60	45	20
Lu2957	1	p2	(AG)18	4	354 356 364 366	Lu2957F	GAGGTTGTCAATTCCGTTGG	60.4	50	19+20
						Lu2957R	GTGTTTACGCTCATCCTTTGC	59.8	47.6	21
Lu2959	1	p3	(AAG)6	2	330 336	Lu2959F	ACCCTGCAACCTATCTGTGG	60	55	19+20
						Lu2959R	AGGATGTTATTGGGCAATCG	59.8	45	20
Lu2963	1	p3	(ATC)6	2	231 234	Lu2963F	GCTTAGGTGGAGCAGGTCAG	60	60	19+20
						Lu2963R	ATGAAATCAGGGGCAGTTTG	59.9	45	20
Lu2965	1	p3	(AAT)8	2	352 355	Lu2965F	GTCGGCGTTCTTGAATTAGG	59.7	50	19+20
						Lu2965R	AGGCCTCTCTTCCATCTTCC	59.8	55	20
Lu2966	1	p2	(AT)10	3	280 288 290	Lu2966F	TTTAGTTGGCAGATCGAGAG	55.7	45	19+20
						Lu2966R	ATACACATTTCACTTAGCTTTGTTAC	55.3	30.8	26
Lu2968	1	p3	(TAA)9	4	334 355 358 361	Lu2968F	TGATGAATTGATGGTGGTGAG	59.4	42.9	19+21
						Lu2968R	CCAACCTTGCATGAACACTG	60.2	50	20
Lu2971	1	p2	(AT)10	3	224 226 230	Lu2971F	CTGCAGCAGAGAACATACGG	59.6	55	19+20
						Lu2971R	ATTTTGCATTTCCACGTTCC	59.8	40	20
Lu2972	1	p2	(AT)9	2	307 309	Lu2972F	ATAACGTCCGACCCTCTCG	60.1	57.9	19+19
						Lu2972R	TTTTGCAGCCTTAGATTCTCG	59.6	42.9	21
Lu2974	1	p2	(TA)15	4	235 237 239 241	Lu2974F	AGAAAAGCTTCTGGCATTCG	59.6	45	19+20

						Lu2974R	CCCCATTGCTATGTTCATCC	60.2	50	20
Lu2975	1	p3	(TCT)7	2	null 204	Lu2975F	GCTTGAGCAGCAAGCTAACC	60.3	55	19+20
						Lu2975R	TGGGCGGAAGAGATAATCAG	60.2	50	20
Lu2976	1	p2	(AG)9	2	344 348	Lu2976F	ATGTCGTCCATTGATTTCCTG	59.8	42.9	19+21
						Lu2976R	TCAACGCCATTCTTTCTTCC	60.2	45	20
Lu2979	1	p3	(TCC)6	2	319 322	Lu2979F	GCCGTAGCACCTTAGATTCG	59.9	55	19+20
						Lu2979R	CAGCAAAGCTGTGAAGAGTCC	60.2	52.4	21
Lu2980	1	p2	(AT)15	2	215 217	Lu2980F	TCTGGATTTGGATTGGTTCTG	59.9	42.9	19+21
						Lu2980R	CTGCTCAATGTTCAGGTCCTC	59.9	52.4	21
Lu2981	1	p3	(AAT)14	3	306 315 318	Lu2981F	AGCAAAAAGCAAACGTAGGG	59.4	45	19+20
						Lu2981R	GGAATCATCTGTAGGTAGGTGAGG	60.3	50	24
Lu2983	1	c	(TAT)7(TGT)9	4	260 263 266 272	Lu2983F	GGACACCACCAACAACAGC	60	57.9	19+19
						Lu2983R	TTCCCTTTTCCATTCATCTCC	60.3	42.9	21
Lu2984	1	p2	(TC)20	2	206 218	Lu2984F	CACCTTTGTGTTTTGCATCC	59	45	19+20
						Lu2984R	ATGGTGTTTGTGTGCGTAGC	59.6	50	20
Lu2985	1	p2	(TA)19	3	null 335 337	Lu2985F	AGGAAACAAGCATGGAGAGG	59.3	50	19+20
						Lu2985R	TTGAATGACATGGTATCACACATAC	58.7	36	25
Lu2991	1	p2	(AG)12	3	245 257 259	Lu2991F	GGGAGAAAGGGGAAAGGAG	60	57.9	19+19
						Lu2991R	TTTGCAGTTTTGGGGGATAG	59.9	45	20
Lu2992	1	p2	(AT)12	4	309 317 319 321	Lu2992F	TTCTACGTCGAGGGTTGAATG	60.1	47.6	19+21
						Lu2992R	CACAATTTTGACACGATGTGG	59.9	42.9	21
Lu2993	1	p2	(CT)16	3	221 231 233	Lu2993F	GATCAGCCACCATCTCTATGC	59.7	52.4	19+21
						Lu2993R	TTTGGATCCTCAACCATTCC	59.7	45	20
Lu2995	1	p3	(CTC)7	2	367 370	Lu2995F	CATAACATGTCGGGCATCC	59.7	52.6	19+19
						Lu2995R	CACAGTGATCTCCACCATCG	60.1	55	20
Lu2996	1	p3	(GAA)11	4	238 247 250 253	Lu2996F	AGCCTCTGCGTTTCTTTCAG	59.8	50	19+20
						Lu2996R	GGCAGACTCTCGCTGGTTAG	60.2	60	20
Lu2997	1	p3	(GAC)6	2	264 267	Lu2997F	AAGAGCATGTGGACCTCACC	60.1	55	19+20
						Lu2997R	TCACAACCCAACCCAACC	60.2	55.6	18
Lu2999a	2	p3	(TTC)10	2	362 365	Lu2999F	TCCCTGCATCACTAAAACACC	60	47.6	19+21
Lu2999b				3	337 339 341	Lu2999R	ACAGGATTTATGGCGACTGG	60	50	20
Lu3001	1	p2	(AT)19	5	305 307 311 320 325	Lu3001F	GAAAGCTGGTAAAAGTGAAGTCAAC	59.8	40	19+25
						Lu3001R	CAAAAGCCGGAATGAAAAAG	59.7	40	20
Lu3003	1	p3	(AAT)12	2	273 276	Lu3003F	AGAATCGGACTCCCTTTTCC	59.5	50	19+20
						Lu3003R	TTTTAGACGTTATCACAGGAGTGC	59.7	41.7	24
Lu3004	1	p3	(AGA)6	2	340 343	Lu3004F	TCTTCAATTGGTGTGCAACG	60.7	45	19+20
						Lu3004R	GAACTCGCTGAGTCGAATGG	60.9	55	20
Lu3007	1	p3	(TCA)7	2	251 257	Lu3007F	TCTCTGCTGACCCTGCATC	60.1	57.9	19+19
						Lu3007R	TCCGTCTTCAGCACAGATTG	60	50	20
Lu3010	1	p2	(CT)9	2	333 337	Lu3010F	CAATCCTTCCATCGATCTCC	59.4	50	19+20
						Lu3010R	TCTAGGATTGCCCGAGAAAG	59.4	50	20
Lu3013	1	p2	(CT)16	3	180 198 200	Lu3013F	GGCATGATATGAGTGTTTAGTTGG	59.8	41.7	19+24

						Lu3013R	TTGATAGAGCTCACAACCTTTCC	59.8	43.5	23
Lu3014	1	p2	(AG)9	2	202 204	Lu3014F	CAAAACCTGTGGTGGTTGG	59.8	52.6	19+19
						Lu3014R	TTTTCTCCAGCTTCCAATCG	60.3	45	20
Lu3015	1	p2	(TA)9	2	285 287	Lu3015F	TTACAGCAATGCAGCTGACC	60	50	19+20
						Lu3015R	TCAAAAGAGACAGAAGAGCAAGC	60.3	43.5	23
Lu3016	1	p3	(TGA)6	2	256 262	Lu3016F	TGCAGCTGTTTGTGCTAACC	60.1	50	19+20
						Lu3016R	GATGGGGTCTTGATGTTTGG	60.2	50	20
Lu3017	1	p3	(TTC)10	4	347 350 460 463	Lu3017F	CAATGAAGATTGCCATCACG	60.1	45	19+20
						Lu3017R	TACCCAGGTCGGAAAAGTTG	60	50	20
Lu3019	1	p3	(CTT)6	2	286 289	Lu3019F	GCATTCTATATGCGCTGCTTC	60	47.6	19+21
						Lu3019R	GGAATAGGATTCGGGCTAGG	59.9	55	20
Lu3020	1	p2	(TA)14	4	269 271 273 275	Lu3020F	TCAAAATTGATGCAAGTATCACAG	59.2	33.3	19+24
						Lu3020R	GGAATTCTGTGCCTTTGAGC	59.8	50	20
Lu3022	1	p3	(AGA)7	3	352 361 367	Lu3022F	GAGGAATTGAGCTCACAGCAG	60.1	52.4	19+21
						Lu3022R	TGCCTCAGCGTGTTTATGAG	60	50	20
Lu3023	1	p6	(CCAAGA)5	2	364 372	Lu3023F	CCAACCAACACCATCATTTG	59.7	45	19+20
						Lu3023R	ACATTGAACCCTTCCACCTG	59.8	50	20
Lu3024	1	p3	(TTC)6	2	null 375	Lu3024F	GAACTTCATTTTCCAGGTACGG	59.9	45.5	19+22
						Lu3024R	TTCATCAGCTTCCCATCTCC	60.2	50	20
Lu3025	1	p3	(TTC)7	2	308 320	Lu3025F	GCACCCCAATTTGGTTAGAG	59.4	50	19+20
						Lu3025R	TTTCCGGCCATGAATTAAAC	59.8	40	20
Lu3026	1	p3	(AAC)6	2	344 350	Lu3026F	GTCAGGATTCGGCTTGTCTC	59.8	55	19+20
						Lu3026R	AGGATGCCAACATCTTTTCG	60.1	45	50
Lu3027	1	p3	(AAG)6	2	349 352	Lu3027F	GTCTCGAAGGAACGGCTATG	59.8	55	19+20
						Lu3027R	AATCCCACCAAACAAACCTG	59.7	45	20
Lu3028	1	p3	(ATA)9	3	252 258 261	Lu3028F	GGCAAGGAGAAACAAAGATCC	60.1	47.6	19+21
						Lu3028R	ACTCTCACTCCCCAAACAGC	59.3	55	20
Lu3030	1	p3	(GAA)7	2	205 208	Lu3030F	AGGAAGAGCGACAAGACGAG	59.7	55	19+20
						Lu3030R	GGTTGATGGCCACGATAGAT	59.8	50	20
Lu3031	1	p3	(TCT)6	2	306 312	Lu3031F	TGCAAAAGGACCATGAATCTC	60.1	42.9	19+21
						Lu3031R	TTTGTTGGTTTGTCCCCTTC	59.8	45	20
Lu3033	1	p2	(AG)11	2	296 302	Lu3033F	TCAAGAAAGCATATGCAAGAACTC	59.9	37.5	19+24
						Lu3033R	TGTGGTACCTCCAGATGTCG	59.5	55	20
Lu3034	1	p3	(AAT)9	3	330 333 339	Lu3034F	AGCAGTCCAAGACGACCAAT	59.7	50	19+20
						Lu3034R	CCTGAAACCAAGCAAGTTCC	59.7	50	20
Lu3035	1	c	(AGA)9ggaggagagattctttcctcaatcatcaaactttggagatggcattttgctttttcatattttt(TC)11	3	280 283 286	Lu3035F	GGGGGAACGAAGAGAGAATC	60	55	19+20
						Lu3035R	GCTTCAGGGAAATCTGCAAC	59.8	50	20
Lu3036	1	p4	(AGAA)5	3	322 330 346	Lu3036F	TCAGATGGTTTGCTGCTGAC	60	50	19+20
						Lu3036R	ATCACCCTTCCCCAAAACTC	60.2	50	20
Lu3038	1	p3	(ATA)7	4	300 303 312 315	Lu3038F	TACCCTTGGACCCAAGACAG	60	55	19+20

						Lu3038R	GCAAAGGTTGGTTTGAATGG	60.3	45	20
Lu3039	1	p3	(CAA)11	2	298 301	Lu3039F	TTCACCGTCATCTCACATCC	59.5	50	19+20
						Lu3039R	TAAGCTCCGGAGACCAAAAC	59.3	50	20
Lu3040	1	p4	(CTAG)5	2	350 362	Lu3040F	CAATGATGAGCCACGTTGAC	60.1	50	19+20
						Lu3040R	TTTGTGGGGAATATTGTCTGG	59.7	42.9	21
Lu3041	1	p3	(CTG)8	2	268 277	Lu3041F	TTTTGGGAATCCGAATGAAG	59.9	40	19+20
						Lu3041R	ATGGCGTTCTCATTTTCGAC	60.1	45	20
Lu3042a	2	p2	(GA)11	3	null 264 266	Lu3042F	CATCCCTCTCTCCAAAGTGC	59.8	55	19+20
Lu3042b				2	null 248	Lu3042R	AGTTTGGAAGTGGGCTAACG	59.2	50	20
Lu3043	1	p2	(GA)16	2	187 201	Lu3043F	TTCCGAGAGAGGAGAGTTGG	59.5	55	19+20
						Lu3043R	CAGAGGCGGGCAGATATAAA	60.2	50	20
Lu3044	1	p3	(GAA)8	2	282 288	Lu3044F	GACCCTCCTCCCAATAATCC	59.6	55	19+20
						Lu3044R	ACATTATGAATCGGCTGTTGG	59.8	42.9	21
Lu3045	1	c	(GCA)8ggatcgtttgaaagcattggagccgga tacatctccctctctctcatcatc(TCT)7	2	299 302	Lu3045F	CCCTTTCCAAGCCTGATAAAC	60	47.6	19+21
						Lu3045R	TCCGTCTTCTCTCTGTTGACC	59.4	52.4	21
Lu3046	1	p4	(TATC)7	2	247 259	Lu3046F	GGCCATTCTTGATTTCTTGC	59.7	45	19+20
						Lu3046R	AAGTCCCCTCCACATGTCTG	60	55	20
Lu3047	1	p3	(TCA)6	2	205 208	Lu3047F	TTTGCTCCGTCTCTGCATC	60.1	52.6	19+19
						Lu3047R	TCTTTTCTGGGTTGTGTCCAG	60.1	47.6	21
Lu3049	1	p3	(CAA)13	3	301 304 307	Lu3049F	GATCCGCCTGATGATGATG	60	52.6	19+19
						Lu3049R	ACCCAACGGAGACCAAATC	59.8	52.6	19
Lu3052	1	p2	(TA)12	4	271 277 281 283	Lu3052F	CTCCACGCTCCTACGTTCTC	60	60	19+20
						Lu3052R	TGTTCCTTAGCTTGCTGCTG	59.4	50	20
Lu3053	1	p2	(AT)15	4	226 228 232 236	Lu3053F	TGGATTCATGCAAACGACTC	59.7	45	19+20
						Lu3053R	TTGGCACCAACAATATAGCATC	59.9	40.9	22
Lu3056	1	p5	(GAAGA)7	2	275 285	Lu3056F	CACAATGGAGGCTCTGATACC	59.6	52.4	19+21
						Lu3056R	GCAACATTACCCAGCAACG	60.1	52.6	19
Lu3057a	2	p3	(AAT)7	2	351 354	Lu3057F	GAACTGTTGGGATAGCGAGTG	59.7	52.4	19+21
Lu3057b				2	287 293	Lu3057R	ATGAGAAGGTCGGGCAAAC	60.1	52.6	19
Lu3059	1	p3	(TTC)8	3	297 306 309	Lu3059F	GAAAAGGCCGATTCTTTGC	59.8	47.4	19+19
						Lu3059R	TGTGGCATATCCACCACAAT	59.7	45	20
Lu3060	1	p2	(AT)11	4	334 336 338 344	Lu3060F	CGAATACAATGGAACCAACG	58.9	45	19+20
						Lu3060R	TTGACCTTCGATGTGTTAAACC	59	40.9	22
Lu3061	1	p3	(GAT)6	2	357 360	Lu3061F	TGGAGGAGACAAGGAGGATG	60.2	55	19+20
						Lu3061R	AATCTACCCCCTGCAAGTCC	60.3	55	20
Lu3063	1	p3	(CAT)6	2	279 291	Lu3063F	AGTGGGTCATCCCTTCAATG	59.8	50	19+20
						Lu3063R	CCGAGGGATCAAAAGATCAA	60	45	20
Lu3064	1	p3	(TTG)10	3	195 201 207	Lu3064F	TCCCTTCATTTGACCAATTC	57.4	40	19+20
						Lu3064R	CAACCAGGAATCAAATGGAC	57.8	45	20
Lu3066	1	p2	(AT)18	2	313 317	Lu3066F	GGGTGAGACGTTCCTTTACG	59.6	55	19+20
						Lu3066R	CACATGTAGATCGCTAGCATACG	59.8	47.8	23

Lu3068	1	p3	(GAA)13	4	348 351 354 357	Lu3068F	TCAGGGTGTTTTGAGTTTCAG	57.8	42.9	19+21
						Lu3068R	GACTCGGTCAAGAATCATGG	58.1	50	20
Lu3074	1	p3	(TGA)10	3	328 332 338	Lu3074F	AGAACCCTGCAGGATCATTG	60.1	50	19+20
						Lu3074R	ACTCCTTTCCGATTCCATCC	60.3	50	20
Lu3077	1	p3	(GAA)6	2	356 359	Lu3077F	AGAGGACCGACCAAAATGC	60.1	52.6	19+19
						Lu3077R	CCTCTCTTCGTGCATTCCTC	60	55	20
Lu3078	1	p2	(TC)19	2	270 284	Lu3078F	AGGGGGAAGACACAGATGAG	59.1	55	19+20
						Lu3078R	CTTAAGGCTGTAAGAAGGTCAATG	58.6	41.7	24
Lu3080	1	p3	(TCT)6	2	209 215	Lu3080F	TTGAACGAAACCCCAGTCTC	60.1	50	19+20
						Lu3080R	CGTCACCGTAACCACATCAG	60	55	20
Lu3082	1	p2	(TA)12	3	190 192 194	Lu3082F	ACACATTGGAGTGTAGCTCAAG	57	45.5	19+22
						Lu3082R	TCACATCACACTGTTTAGTAGATGG	58.3	40	25
Lu3083	1	p2	(TA)13	3	359 361 363	Lu3083F	TGGTGTGGCAATATGGTTTG	60.2	45	19+20
						Lu3083R	TGAACCATCTTCAAGTGCTGAC	60.3	45.5	22
Lu3085	1	p3	(TGT)6	2	328 334	Lu3085F	CAGTGGGAAAGGTCAGGAAC	59.5	55	19+20
						Lu3085R	TCGGTACCCAAACAAAAAGC	60	45	20
Lu3090	1	p2	(CT)10	2	188 190	Lu3090F	GGTGGAGAAGAGGAGCTGTG	60	60	19+20
						Lu3090R	CTAATCCCCGCTCTCAAAATC	60	47.6	21
Lu3091	1	p2	(TA)18	5	263 265 269 273 277	Lu3091F	GATTGGAAATGGGAGACTGG	59.3	50	19+20
						Lu3091R	GTAACACTAACAGCGCCAACC	59.7	52.4	21
Lu3092	1	p3	(ATA)12	3	214 220 223	Lu3092F	ATGATACCACCAGCACAGCA	60.1	50	19+20
						Lu3092R	CGAGGTTAAAATGGAATACACACC	60.8	41.7	24
Lu3094	1	p2	(GA)10	3	192 194 196	Lu3094F	TGGTTGGGAGTGTTTTTGAAC	59.9	42.9	19+21
						Lu3094R	CTTCCTCGAGATCCGAACAC	59.8	55	20
Lu3095	1	p4	(GTTA)6	2	339 343	Lu3095F	CCCACTATTGGGTCGACAAC	60.2	55	19+20
						Lu3095R	CAACACTTTATTTACCAACCCACAC	60.8	40	25
Lu3097	1	p3	(AGA)11	4	280 295 298 301	Lu3097F	AAGGAGGGGGAGAAAACAAC	59.4	50	19+20
						Lu3097R	GGGTTGTTGGGAAGAAGAAAC	59.8	47.6	21
Lu3099	1	p2	(AT)9	2	247 249	Lu3099F	AAGTTGCGCCATTAGTGTCC	60.1	50	19+20
						Lu3099R	GAACCACCCGCTAAACTCAG	59.7	55	20
Lu3100a	2	p2	(CT)9	2	313 317	Lu3100F	CGTTTCAATCGGTGTCTGG	60.1	52.6	19+19
Lu3100b				2	304 306	Lu3100R	CAGGCCATGTACGATGTTTG	60	50	20
Lu3103	1	p3	(TGA)6	2	343 346	Lu3103F	CCATCAAACACCATTCAAACC	60.1	42.9	19+21
						Lu3103R	CCCAACTCACTCACCTCCTC	59.7	60	20
Lu3109	1	p3	(AAG)11	2	285 288	Lu3109F	AGCAAATCTGCAGGAAAAGC	59.6	45	19+20
						Lu3109R	GCAAATTTTGAAGGCTCCTG	59.8	45	20
Lu3111	1	p2	(TA)9	5	null 290 292 294 296	Lu3111F	TCCCATCTAGGTGGAAAGGAC	60.3	52.4	19+21
						Lu3111R	TGTGGAAAGGGTATGGCTTC	59.9	50	20
Lu3113	1	p2	(AG)11	3	251 253 261	Lu3113F	CTTGGTGACTGGCAATCTAGC	59.9	52.4	19+21
						Lu3113R	GTCCCACCCTTCAATTCTTG	59.4	50	20
Lu3114	1	p2	(GA)11	2	268 270	Lu3114F	GGATTCATGGACGAGAGGAG	59.6	55	19+20
						Lu3114R	TCCCTCCCACTCATCAAAAC	59.9	50	20

Lu3116	1	p2	(TA)9	3	342 344 348	Lu3116F	AGGTAACGTACCGCTTATATTTTACA	58.7	34.6	19+26
						Lu3116R	ATACGCGAGATGGACACGTA	59.2	50	20
Lu3117	1	p2	(GA)13	2	206 208	Lu3117F	CCAAAAAGAAAGACGGTTGG	59.6	45	19+20
						Lu3117R	TCCTAAAAACTTCCACCACTCC	59.5	45.5	22
Lu3119	1	p2	(AT)19	5	315 317 323 325 329	Lu3119F	ATCGTTTTGCATCGTGATTG	59.5	40	19+20
						Lu3119R	GATCGAGCTATTGTATTGTTCACG	60	41.7	24
Lu3120	1	p2	(AT)17	3	277 279 283	Lu3120F	AAACAGGCAAACCAATGTCC	59.8	45	19+20
						Lu3120R	ACTCGACCCACATCCAACTC	60	55	20
Lu3123	1	p2	(TA)13	4	355 357 359 363	Lu3123F	CATTGAGTGTGTTAGTCGAACCAT	60.3	41.7	19+24
						Lu3123R	GAGATATCCATTGATACGCTCCA	60.3	43.5	23
Lu3125	1	p3	(AAG)11	2	313 316	Lu3125F	TTTTTACCAGGGGATTGCAG	59.9	45	19+20
						Lu3125R	AAAGACGGTCGATGGTTCTG	60.1	50	20
Lu3127	1	p2	(TA)15	3	227 241 245	Lu3127F	AACGCTGCCCTAGTGATTTG	60.3	50	19+20
						Lu3127R	ATCTCCCCAAACGCATCTC	60	52.6	19
Lu3130	1	p3	(ATT)8	2	267 270	Lu3130F	TTCACCGTGGTTTGTTGTAGA	59.1	42.9	19+21
						Lu3130R	GTTTTGGGTCGGATAGCAAG	59.6	50	20
Lu3132	1	p2	(AT)13	4	198 200 202 230	Lu3132F	AGATCTTATCCTCAAACGAACAGG	60	41.7	19+24
						Lu3132R	TGTTGTTGCTCAAACTTAGTACTGG	59.8	40	25
Lu3134	1	p3	(CAG)8	2	240 249	Lu3134F	ACAATCGACATGGGAAAAGC	59.9	45	19+20
						Lu3134R	CAAGTAAAACAGCACCAACAGC	59.9	45.5	22
Lu3143a	2	p2	(GA)9	2	null 291	Lu3143F	CGAATCTTCTTCGCTTCACC	60	50	19+20
Lu3143b				2	null 284	Lu3143R	CGAGACCGAGACAGTGAAATC	59.9	52.4	21
Lu3144	1	p3	(TAC)6	2	258 264	Lu3144F	TCACACCGCCCAATCATAC	60.3	52.6	19+19
						Lu3144R	TGCACACGCTCTAACGAATC	60	50	20
Lu3146	1	p2	(AT)12	2	321 325	Lu3146F	AACCCAGATGCCTACGTTTG	60	50	19+20
						Lu3146R	CCGAACCCACTTTAACCTAGC	60	52.4	21
Lu3148	1	p6	(CTGCGA)5	2	250 262	Lu3148F	ACGTGGTCGACGTAATACCC	59.7	55	19+20
						Lu3148R	AACCCGCTACTCACCGACT	59.7	57.9	19
Lu3149	1	p3	(TCC)8	2	250 253	Lu3149F	TCCTCAATCTCTCCCTCACG	60.3	55	19+20
						Lu3149R	TTGCAGACAGTCACACGTCA	60.1	50	20
Lu3150	1	p3	(TCG)7	3	360 366 369	Lu3150F	CCGCAGCTGAATTATATTTGG	59.6	42.9	19+21
						Lu3150R	TCGACGACCTACCCATTTTC	59.9	50	20
Lu3151	1	p3	(AAT)6	2	325 328	Lu3151F	TTTCGAGTCGGTGTTCATTTC	60.1	42.9	19+21
						Lu3151R	AAGTGTAGAATCCCCCACCAC	60.1	52.4	21
Lu3152	1	p3	(ATA)10	2	246 261	Lu3152F	TCCTCGCATTACAAGTCTTCC	59.3	47.6	19+21
						Lu3152R	ACCGTGAATTGCTTGATCG	59.7	47.4	19
Lu3153	1	p4	(ATAA)6	3	270 278 284	Lu3153F	TCCACGGGTGGATATTTCA	59.7	47.4	19+19
						Lu3153R	GTACGGAAGGGGATGATGTG	60.2	55	20
Lu3156	1	p3	(AGA)11	3	333 339 342	Lu3156F	TCGCCAGCCTTCTTCTTTAG	59.7	50	19+20
						Lu3156R	AAACGCGGGTATTAACAACG	59.9	45	20
Lu3157	1	p2	(AT)11	7	220 224 226 232 234 236 238	Lu3157F	ACGCATCCTTAGTGCAATCC	60.1	50	19+20
						Lu3157R	CGTAGCCCTTGTCTTCTTGC	60	55	20

Lu3160	1	p2	(AG)9	2	null 249	Lu3160F	GGCTTTGATTCCTGGATGG	60.4	52.6	19+19
						Lu3160R	CTGAGTTGGTATAGCTTCTTTGAGG	59.9	44	25
Lu3165	1	c	(AGA)6ggtatgtcgatctcggttgcatcatcgtc aacagcaagatcttcatctggactttgggaactttg ctcgccgccgatctcctcaccggac(TCA)6	2	null 292	Lu3165F	ACTTAGCCACCGACAACGAC	60.2	55	19+20
						Lu3165R	GGGGTTTAGGGTGATGGTG	60	57.9	19
Lu3167	1	p2	(CT)16	4	345 347 351 353	Lu3167F	ACGTGGCCAAGTAAACGAAA	60.5	45	19+20
						Lu3167R	AACGGGATTTAACAGCAGGA	59.6	45	20
Lu3169	1	p6	(TGGGCT)5	2	216 234	Lu3169F	CTGGCCCATCAACTCATTG	60.1	52.6	19+19
						Lu3169R	GCCGACGAGGTAAATGGTAA	60	50	20
Lu3171	1	p4	(GGTT)10	2	224 228	Lu3171F	TTATGGGGCGCTTAAGTCTG	60.2	50	19+20
						Lu3171R	TGCATGCTTTGATGCTTACC	59.8	45	20
Lu3176	1	p3	(CTG)6	2	236 242	Lu3176F	CGTTTGTGCTAGTGGGGACT	60.2	55	19+20
						Lu3176R	TTCTTCCTTCCTTCCCTTTTG	59.7	42.9	21
Lu3178a	2	c	(AAT)6tcacctcaatatatatctcaaacaccac gtacattag(TA)12	2	237 239	Lu3178F	ACTCCACGAACACCACTTCC	60	55	19+20
Lu3178b				3	276 278 280	Lu3178R	CAGGTACTCATTTCATTCCCTTG	59.9	43.5	23
Lu3180	1	p2	(CT)20	6	246 260 262 264 266 268	Lu3180F	ACGGTCAAAGACGAAAGGTG	60.1	50	19+20
						Lu3180R	GAAAGGACAAAGCTGCCATC	59.8	50	20
Lu3181	1	p4	(ACTC)5	3	358 360 364	Lu3181F	ATGGGTTAGTCACGGAGTCG	60	55	19+20
						Lu3181R	TGCTGTGCATGGCATATTG	60.2	47.4	19
Lu3182	1	p4	(CTAT)5	2	244 256	Lu3182F	GTTTCATTTTTCTGGGCATTTC	59.8	36.4	19+22
						Lu3182R	CCCCTCCATCTCTATCTCTATATATCC	60	44.4	27
Lu3184	1	p3	(TGA)6	3	356 362 368	Lu3184F	CTGCTGAAGCTGCCTTAGGT	59.8	55	19+20
						Lu3184R	AAACCTTGACCTCCCATTCC	60.2	50	20
Lu3185	1	p3	(ATT)7	2	370 373	Lu3185F	GCCTGTCTGATAAGGACAAGAAA	59.8	43.5	19+23
						Lu3185R	CCGAACAGGAAATCATTTAACG	60.7	40.9	22
Lu3186	1	p2	(TA)13	4	335 337 339 341	Lu3186F	GACTGTTGTCCAACGAGGTG	59.2	55	19+20
						Lu3186R	GAACTGACCGTTGCCAAGTT	60.2	50	20
Lu3187	1	p3	(GTT)8	2	223 226	Lu3187F	CCTTGAGCTAGATGCCAAGC	60.1	55	19+20
						Lu3187R	CAAAAGAGAGGGCAGCTGAG	60.3	55	20
Lu3188	1	p2	(AT)12	2	250 254	Lu3188F	CTAAATCGTAAAAATTAAACGACAC	55.7	28	19+25
						Lu3188R	ACGTAAACATACCAGTGATGC	55.2	42.9	21
Lu3189	1	p3	(ATA)6	3	239 245 248	Lu3189F	TCCAACTCGGCTTTGATACC	60.1	50	19+20
						Lu3189R	GTTAGATACGGACAGCTTGCAG	59.1	50	22
Lu3192	1	p3	(TCA)6	2	220 223	Lu3192F	TACCAGCATTGGATCGAACA	60.1	45	19+20
						Lu3192R	AACGGGAACTCAAGTTGGTG	60	50	20
Lu3193	1	p3	(TCC)6	2	334 340	Lu3193F	GAATCGACATCGCTCTCCTC	59.9	55	19+20
						Lu3193R	TAGTGGTGCTGCTTCAAACG	60	50	20
Lu3195	1	p4	(AATA)5	3	263 266 270	Lu3195F	TGGGGTTTAAGGCTTCACTG	60.1	50	19+20
						Lu3195R	GCCCACCCCATCAAGTTAC	60.2	57.9	19

Lu3199a	2	p3	(TCT)7	3	223 229 232	Lu3199F	TCCTCCTCTTCCACTCTTCTTTC	60.4	47.8	19+23
Lu3199b				3	329 335 338	Lu3199R	TGAAGCAATGGAGGAAAACC	60.1	45	20
Lu3200	1	p3	(TTC)9	2	240 243	Lu3200F	TGTAGCACAGCCGACTCTTG	60.2	55	19+20
						Lu3200R	CTCTGGATGGGAAGCCATT	60	52.6	19
Lu3201	1	p3	(AAT)9	3	214 235 238	Lu3201F	CCCTTTTAATGCATGCTTGG	60.4	45	19+20
						Lu3201R	CACCTCATGTCGCTTAACCA	59.7	50	20
Lu3202	1	p3	(ATT)9	2	237 240	Lu3202F	TGGCTGAAAGCATTCAAGTG	60	45	19+20
						Lu3202R	TCTCGTTACTCCCCACCAAC	60	55	20
Lu3204	1	p3	(TTC)9	2	362 365	Lu3204F	ACCCCACAACCTTTTTCTCC	60.2	50	19+20
						Lu3204R	GTTGTTTGTGGGGACAATCC	60.1	50	20
Lu3205	1	p2	(TA)11	3	352 354 356	Lu3205F	GCATTGCTTTCATCCATGTG	60.1	45	19+20
						Lu3205R	TGTCCAAATGAAGTCCATGC	59.5	45	20
Lu3206	1	p2	(AG)14	2	274 282	Lu3206F	ATCCCGTCGTCTTGTACAGC	60.1	55	19+20
						Lu3206R	TGCGTGCGGCGTTT	60.7	64.3	14
Lu3207	1	p2	(TC)15	2	234 236	Lu3207F	ATCCAAATCACGAACACAAGG	59.8	42.9	19+21
						Lu3207R	TCGTGCCACTTTATCACTGG	59.7	50	20
Lu3209	1	p2	(TA)9	4	206 214 224 226	Lu3209F	CATCCAAGCCAATGGTCTCT	60.1	50	19+20
						Lu3209R	ATAGGGCTGGAGCAGTGAGA	60	55	20
Lu3210	1	p2	(TA)10	3	236 238 242	Lu3210F	TTTTGATCATTTCCCCAAGC	59.9	40	19+20
						Lu3210R	GCCAGTTGCTTTCTTTGGTC	59.9	50	20
Lu3211	1	p3	(TAT)8	2	315 321	Lu3211F	AATAGAAGCAGACAGGCTGAGG	60	50	19+22
						Lu3211R	AACAACTGAATCACCCATTCG	59.8	42.9	21
Lu3212	1	p2	(AT)20	5	231 239 249 251 255	Lu3212F	GGTTTAATTTGCAGTCCAGTACG	59.9	43.5	19+23
						Lu3212R	GAAAAAGCCAGGAGTCATGC	59.8	50	20
Lu3213	1	p2	(AT)10	2	313 315	Lu3213F	TTCTCCTGAAATCATGCTTCG	60.3	42.9	19+21
						Lu3213R	AGTCCAGAGGCGATTCAAAG	59.4	50	20
Lu3216	1	p2	(TA)10	5	265 273 275 277 279	Lu3216F	GAGGTGGAGAAATTTGGTTGA	59	42.9	19+21
						Lu3216R	CGTAGGTTATCGCTTGAGAGC	59.1	52.4	21
Lu3217	1	p4	(AAAG)7	2	294 298	Lu3217F	AAAAGGGAGAGTTCCCCAAG	59.5	50	19+20
						Lu3217R	AGCAGCCAGCAATCTGTACC	60.4	55	50
Lu3218	1	p3	(CAG)8	3	212 218 224	Lu3218F	ATGATGGGGATTCCTACCG	59.6	52.6	19+19
						Lu3218R	GTTCCACTTACCGCTTCACC	59.6	55	20
Lu3219a	2	p3	(TCT)6	2	362 374	Lu3219F	CCAGTTTTTCTCCAGCAACC	59.7	55	19+20
Lu3219b				2	312 315	Lu3219R	TCACCAGCTCTTCACTACAAGG	59.5	50	22
Lu3220	1	p2	(TC)15	3	332 334 336	Lu3220F	GTGACGATTGGAATGACGTG	60	50	19+20
						Lu3220R	TCACTTACCGGCGATAGAGC	60.4	55	20
Lu3222	1	p2	(TC)14	2	274 278	Lu3222F	ACCCAGACGGAAAGACTGC	60.3	57.9	19+19
						Lu3222R	ACCACCAATCCTCGTACACC	59.7	55	20
Lu3223	1	p2	(AG)13	3	359 361 363	Lu3223F	GTTCTCCTTCGCCTTCTTCC	60.3	55	19+20
						Lu3223R	CAGCCAAATCTCCTCTCACC	59.8	55	20
Lu3225	1	p4	(ATGG)8	2	215 223	Lu3225F	CAGTTCCCCAAAATCACGTC	60.4	50	19+20
						Lu3225R	TTGCAGCTAGCGTACAGTGC	60.4	55	20

Lu3226	1	c	(ATTA)5ctaatatcagcacttggttcgtctatgtc accgac(AG)13	2	280 282	Lu3226F	CGACGCGAATGTGTTCATAC	60.1	50	19+20
						Lu3226R	CAGCACCGACAGTAAGGAGAG	60.1	57.1	21
Lu3228	1	p3	(AGA)17	5	183 189 195 198 201	Lu3228F	GGCTGATGACATGGTGATTG	59.9	50	19+20
						Lu3228R	CTTTGGATTCATCGCCTCTC	59.8	50	20
Lu3229	1	p2	(AT)12	3	332 336 338	Lu3229F	AATATCCAAGCAGCCTTTGC	59.3	45	19+20
						Lu3229R	GTGGTCTCGGAGCAAGACTC	60	60	20
Lu3231	1	p3	(ACC)6	2	296 299	Lu3231F	TGCCATGAAGCAGAGGAAG	60.1	52.6	19+19
						Lu3231R	AGGTCGAACTTGCACATGG	59.7	52.6	19
Lu3232	1	p3	(AGG)6	2	265 268	Lu3232F	AACAGGGATGGTGATTGCTC	59.9	50	19+20
						Lu3232R	TAACCTGGAGGAGGATGGTG	59.9	55	20
Lu3234	1	p5	(CTTCT)5	2	250 325	Lu3234F	CGCCATCTTCCATGTTTTCT	60.1	45	19+20
						Lu3234R	GACAAGAACATGGGCCACTT	60	50	20
Lu3235	1	p2	(GA)11	2	215 219	Lu3235F	CAACATCAACGCAGAAGAAGAG	60.1	45.5	19+22
						Lu3235R	AATTGGCGAGAAACCCAAC	59.9	47.4	19
Lu3236	1	p2	(TA)9	2	218 220	Lu3236F	CCGTTCAATGCCGAATTTAC	60.3	45	19+20
						Lu3236R	GCCAAACGGATTGGAAAAC	60.3	47.4	19
Lu3237	1	p3	(CAG)9	2	234 237	Lu3237F	GGAGAAGAGGGGAATTGAGC	60.2	55	19+20
						Lu3237R	TTGCAGCCTTTCTTTGGTTC	60.4	45	20
Lu3238	1	p3	(GAA)7	3	308 311 317	Lu3238F	AATGGAGGTGAGTGTGAGTGG	60	52.4	19+21
						Lu3238R	GAAGGGGAATTCTGGTACAGC	60	52.4	21
Lu3239	1	p2	(CT)9	2	363 365	Lu3239F	GACTGAATTGGAGCTTTGTGG	59.7	47.6	19+21
						Lu3239R	GTCCGACCATGCAGAATAGG	60.5	55	20
Lu3240	1	p2	(TA)11	3	294 304 306	Lu3240F	ATGAACCGAGGATGACGAAG	60.1	50	19+20
						Lu3240R	TTTGTAGCATTCCACACACCTC	60	45.5	22
Lu3244	1	p2	(TA)11	3	260 300 304	Lu3244F	ATCAAGCAAACCCCGACTC	60.1	52.6	19+19
	1	p2	(AG)16	3	194 196 198	Lu3244R	TGCTGAAGGATATTGGTGTCTG	60.1	45.5	22
Lu3248	1	p3	(GGA)10	2	209 212	Lu3248F	GCCAACGAGATCAGCTAAGG	60	55	19+20
						Lu3248R	CTTCCGATCATGGAGAAAGC	59.8	50	20
Lu3250	1	p6	(TGGGCC)5	3	162 174 180	Lu3250F	CTTAATAGGCTGGTGGTGCAG	59.8	52.4	19+21
						Lu3250R	AAACAAGCCTCCAAAACACG	60.1	45	20
Lu3251	1	p3	(TCA)6	2	297 300	Lu3251F	TGCAGCAAAGTTAGCAGGTG	60.2	50	19+20
						Lu3251R	CTGATCACGCTTTTGGAGTG	59.4	50	20
Lu3252	1	c	(AT)18aactcttatctccttc(TCT)6	4	202 204 210 212	Lu3252F	AAGACGTCCCCTTTGGTATG	58.9	50	19+20
						Lu3252R	GGAATCGCTGAAGATGGAAC	59.6	50	20
Lu3256	1	p3	(AAT)7	2	273 276	Lu3256F	TCCAACCAGGCTCTGATACC	60.1	55	19+20
						Lu3256R	GGAACAGGAAGGAAGGAACC	59.9	55	20
Lu3262	1	p3	(AAT)12	2	195 198	Lu3262F	GTCGACATTAGTAGGTGTTCCTG	57.8	47.8	19+23
						Lu3262R	TTTCCCTGCTATTGGATAAGAG	57.6	40.9	22
Lu3266	1	p3	(GTT)10	4	199 205 211 214	Lu3266F	TTACTTGTATGTAGCTTCGAG	50.2	38.1	19+21
						Lu3266R	GCTTAATCTCTGACTTCCTC	50.8	45	20
Lu3267	1	p2	(TA)11	5	347 349 351 355 357	Lu3267F	CTCTTTGGGATGATGACTTGC	59.7	47.6	19+21

						Lu3267R	ATCTGTCGGCTACGACAAGG	60.3	55	20
Lu3269	1	c	(AT)15(AG)18	3	264 266 277	Lu3269F	CACAATTGCAGATCCTGTGG	60.1	50	19+20
						Lu3269R	CTTTCTTCTGTGGTCCTCACG	59.9	52.4	21
Lu3270	1	c	(ATC)6ggtgtttcatttcatcatcatttgcagagct ggttcaagagctcctt(CTG)6	2	296 300	Lu3270F	ACGCGCTGTGTATTATCCATC	60	47.6	19+21
						Lu3270R	ATTGCGACAATCTCCCAGTC	60.1	50	20
Lu3271	1	p2	(AT)12	2	362 368	Lu3271F	GGAAAAAGGTCTCCAATGAGG	59.9	47.6	19+21
						Lu3271R	GGAGGAAGATTTGGTCCTTTG	59.9	47.6	21
Lu3276	1	p4	(AAAG)10	2	344 348	Lu3276F	TTGGGGCAGAACTTAACTGG	60.1	50	19+20
						Lu3276R	CGACTAATTCCCGCCTAGC	59.8	57.9	19
Lu3278	1	p3	(AGA)7	2	215 224	Lu3278F	CGTCCGAGTCCTTGAAGAAG	60	55	19+20
						Lu3278R	CTCCCTCAACAACCATACCG	60.4	55	20
Lu3279	1	p2	(AG)9	2	240 244	Lu3279F	AGGAAGCAGCTTTGACTTGC	59.8	50	19+20
						Lu3279R	CACATGCCATACTCCACACC	59.8	55	20
Lu3280	1	c*	(AT)17(ATGT)9*	7	329 358 360 375 415 424 454	Lu3280F	CCACCTCCAACTTCTTCTCG	59.8	55	19+20
						Lu3280R	CGCCGTACGAATTACTCTTTTC	60.1	45.5	22
Lu3281	1	p2	(CT)16	3	234 236 238	Lu3281F	CACCATTGACATCGATCTGG	59.9	50	19+20
						Lu3281R	TTTCGCCTTTATCCTGTTGG	60.1	45	20
Lu3283	1	p3	(TAA)8	3	336 339 348	Lu3283F	AGGCTAAATGCACTCAATAATCTG	58.9	37.5	19+24
						Lu3283R	GTACGCACGCTTGTACTTGG	59.4	55	20
Lu3287	1	p4	(CAAC)5	3	310 312 314	Lu3287F	ATCCACAGCATCGCTTCAC	59.8	52.6	19+19
						Lu3287R	TTTCCAGGTCAGGCTTATGG	60.1	50	20
Lu3288	1	p4	(GAAG)5	2	305 309	Lu3288F	AAGGAAGTGGGTTGTGGTTG	59.9	50	19+20
						Lu3288R	CACCACTGTGTCCCTGTGTC	60	60	20
Lu3289	1	p2	(AG)15	3	213 221 225	Lu3289F	GATTCCGACGTGATGATCG	60	52.6	19+19
						Lu3289R	TCCATCCATCCATCCTCTTC	59.8	50	20
Lu3290	1	p3	(CTT)7	2	263 269	Lu3290F	CCAGACCCAAGAAGACAACC	59.5	55	19+20
						Lu3290R	CGTGGAAGAACAGAAGAGACG	60	52.4	21
Lu3291	1	p3	(AAT)13	2	339 342	Lu3291F	AAGTAAGCACGGGGAGAGG	59.3	57.9	19+19
						Lu3291R	CGCAGCTAAAACTCGTAGGG	60	55	20
Total	720			1966						
Average				2.73						

EST-SSRs

Marker	No loci	SSR type	SSR sequence	No alleles	Observed allele size	Primer	Sequence (5' -> 3')	Tm (˚C)	GC content (%)	Length (bp)
Lu853	1	p2c	(A)12gag(GA)10	2	136 140	lu853F	GAAGGAAATTAAAAGAAGTGAGAGAAG	58.7	33.3	19+27
						lu853R	GCTCGCAATCTTCCCGTCT	62.8	57.9	19
Lu857	1	p2c	(GA)7gggttagttt(GA)7gggagagagagaaaaagataaaaagagagagagaaagacggagagacacagagagagaaaggcgaaatttttttgt(AG)11tagcaggggccagggcaggcggtggaagtggtggtgtttgctgtttggtttgtttgtttcttctta(T)10	3	354 362 376	lu857F	GAGAAGGTGGGAGAGGGTTA	58.2	55.0	19+20
						lu857R	CGTCGGTGAGTTAGGGTTTG	60.5	55.0	20
Lu859	1	p3/6c	(ATT)14gtatatgaatat(AATATG)5	4	197 200 206 209	lu859F	GATTCTCCGCAGCTCTGTTC	60.1	55.0	19+20
						lu859R	TACAGCAAACAGCTCCATCC	58.9	50.0	20
Lu861	1	p3c	(ACG)6cagattaataacaacacacaaaccaaattggattttcatggattttttccaatttcttttaaagggttttcc(TCT)5	2	372 378	lu861F	CCAGAGGTGGTGGAAGAAGA	60.2	55.0	19+20
						lu861R	GGTGGAGAGGAGATGGTGAA	60.0	55.0	20
Lu866	1	p3c	(CTC)6catcttttatcccttcttctgaaacacttgt(TTC)5	2	342 345	lu866F	CGTCCGAGTTCAGTCAGGAT	60.3	55.0	19+20
						lu866R	AAGAAGAAGCCGCAGAAATG	59.6	45.0	20
Lu867	1	p3c	(CTC)7cagcgccgccgttaactcctccgccaccc(CCT)5tccgccaccgatgatgtcgaacatgtctg(ATT)7gatgttaac(ATT)5(GTT)6	2	381 393	lu867F	GACGCTAGCTTTGTCCCTTG	60.0	55.0	19+20
						lu867R	ATTCTGGTCCACGTCGAAAC	60.0	50.0	20
Lu868	1	p3c	(CTT)5ctcttcaaatgtgccatc(GCT)5gttattatcattg(TTA)9	2	277 280	lu868F	TTGTGCCCGATGATGATAGA	60.0	45.0	19+20
						lu868R	ATGTGCCCTCAGATCAGTCC	60.1	55.0	20
Lu869	1	p3c	(CTT)5ttccaatcctcttacca(TTC)7	2	195 201	lu869F	TGATCAAAAGTTCCTCTTATCTTTCA	59.7	30.8	19+26
						lu869R	GGTTGTACTGGGGTTGATGG	60.1	55.0	20
Lu870	1	p3c	(GAA)6(AAG)7	2	241 244	lu870F	TGAAACGAAGAACTGGTCCAA	60.7	42.9	19+21
						lu870R	ACGTCTTCGAAAGTGCCTGT	59.9	50.0	20
Lu871	1	p3c	(GAA)6gac(GAA)12	2	391 394	lu871F	AAGAGTATTGCGGGAGAGCA	60.0	50.0	19+20
						lu871R	AAGAAACTCGGTGGTGATGG	60.0	50.0	20
Lu872	1	p3c	(GGA)5cggcggaggaatgattatctcggaagcgcagaaggttctgcttgcagagctggataagttgagatccgaggagatgg(AGA)7	2	385 388	lu872F	AGGAAGCGAGGAGAAGAAGG	60.1	55.0	19+20
						lu872R	GATTCCGATGAACTCGAGGA	60.2	50.0	20

Lu873	1	p3c	(T)18aatttattactattactactattattattattaatt caaccacaacgcataatttattag(TAT)6	2	null 299	lu873F	GTTTTCCCAGTCACGACGTT	60.0	50.0	19+20
						lu873R	AGCTTGTGATGCTGATGGTG	59.9	50.0	20
Lu885	1	p2	(CA)9	2	480 490	lu885F	TTTTGCAACACCACCAATGT	59.9	40.0	19+20
						lu885R	GCAGCATCAATGAAGAAGCA	60.1	45.0	20
Lu886	1	p2	(AT)9	6	195 197 203 211 223 231	lu886F	CTCCAAACACTCTCGTTTATTTATTT	58.4	30.8	19+26
						lu886R	GTGAATGCTCTTGGCCTTGT	60.3	50.0	20
Lu887	1	p2	(AT)9	2	196 204	lu887F	TTTTAGACAATCGCAATA	47.4	27.8	19+18
						lu887R	GGGTTCATCCATTAGGTCCA	59.6	50.0	20
Lu891	1	p2	(TA)9	3	216 216 220	lu891F	ACAACAAGCGTCAAGTGCAG	60.1	50.0	19+20
						lu891R	AAATTAAAGGGCTGGCAACA	59.6	40.0	20
Lu893	1	p2	(TA)9	2	218 222	lu893F	GAGGGGGAAGAGAAAAGAACA	59.7	47.6	19+21
						lu893R	TGAACTTCGCTCCCATTTTT	59.7	40.0	20
Lu895	1	p2	(CT)9	3	317 319 333	lu895F	CGTCCGAGAGAGTGAAACCT	59.5	55.0	19+20
						lu895R	CCGTTTGGATTCAGGTTCTG	60.5	50.0	20
Lu896	1	p2	(AT)9	2	272 278	lu896F	GTTTGCTTGCTGGACTCCTC	60.0	55.0	19+20
						lu896R	TCCTAATCCCTCGGAACAAA	59.5	45.0	20
Lu897	1	p2	(TA)9	2	341 343	lu897F	CTAACGACCGGTTCATGGAT	59.8	50.0	19+20
						lu897R	AATTTACATACAAGCTACACAAAAA	54.5	24.0	25
Lu899	1	p2	(AT)9	3	209 211 213	lu899F	CTGCATGCATGTCCAGTACG	61.3	55.0	19+20
						lu899R	CAGGCTTGGAGGATTATTGG	59.5	50.0	20
Lu900	1	p2	(AT)9	2	344 354	lu900F	CTTTCTTCAACATAACCGCAAC	58.8	40.9	19+22
						lu900R	AGCTTGCAAAAGTTGGCTTC	59.6	45.0	20
Lu902	1	p2	(CT)10	3	null 206 216	lu902F	AAGAAACCATCGACGGTGAG	60.1	50.0	19+20
						lu902R	GGATACAAAGGCAGATTCTT	53.5	40.0	20
Lu903	1	p2	(AT)10	2	214 216	lu903F	AGTTTCCCAAGTTCCCCAAG	60.3	50.0	19+20
						lu903R	CGATTAGATGGAAGGGAGGAG	60.0	52.4	21
Lu905	1	p2	(AT)10	2	209 211	lu905F	GCGTAACGCCTTATTTCATAATTCC	63.0	40.0	19+25
						lu905R	CCAATTCGACGAAGAGAAGC	60.0	50.0	20
Lu906	1	p2	(TA)10	2	null 248	lu906F	TGGTGTCTGTGCCTCTGAAG	60.0	55.0	19+20
						lu906R	TGAAGATGACAACAGTGGAAGA	58.3	40.9	22
Lu908	1	p2	(TC)10	2	296 314	lu908F	CACCCACCCATCTTACAACTG	60.3	52.4	19+21
						lu908R	GACGACGACGAGCAGTATGA	60.0	55.0	20
Lu910	1	p2	(AT)11	3	482 486 488	lu910F	TCACTGCAGTACAGCACACC	58.4	55.0	19+20
						lu910R	TTAACCCTCACGCCTACACC	60.0	55.0	20
Lu912	1	p2	(TA)11	3	null 390 392	lu912F	AAGCTGGAGGCATATGTTGG	60.1	50.0	19+20
						lu912R	CCTTTGCTACCCCATCTTCA	60.1	50.0	20
Lu913	1	p2	(AT)11	2	216 222	lu913F	TGCCAGTCACGATTCAGAAG	60.0	50.0	19+20
						lu913R	GCATCTCCCTAAATCAAAAT	53.5	35.0	20
Lu917	1	p2	(AT)12	2	349 365	lu917F	GTTTCTTCTCCTGCGTCCAC	59.9	55.0	19+20
						lu917R	AACCCATTCGAACATCCCTA	59.2	45.0	20

Lu918	1	p2	(GA)12	2	null 223	lu918F	GCACAGACGACTAGTACCGAGA	59.6	54.5	19+22
						lu918R	CCTACTCCCCCTCCTTCTTG	60.1	60.0	20
Lu919	1	p2	(TA)12	3	266 268 272	lu919F	GGGATTCCAGTGACCAAAAT	58.7	45.0	19+20
						lu919R	TGAAGGAAGATAGGCGATGG	60.2	50.0	20
Lu920	1	p2	(GA)12	3	360 362 364	lu920F	TTAACACAGGGGTTGGAGGA	60.3	50.0	19+20
						lu920R	AGCAGGAAGCAGAATCAGCA	61.2	50.0	20
Lu921	1	p2	(CT)12	2	368 370	lu921F	GTGCTGTGCCTGTGAAAAAC	59.3	50.0	19+20
						lu921R	AGTCCAATGGAGGTGTGGAG	60.0	55.0	20
Lu922	1	p2	(TC)12	3	204 236 250	lu922F	GCAACTCCTCTCCACTCCAA	60.4	55.0	19+20
						lu922R	AAGTTGTCGGAGGTGGTGTC	60.0	55.0	20
Lu923	1	p2	(TC)13	2	379 381	lu923F	CAACGACACCACCCTTTTCT	60.0	50.0	19+20
						lu923R	AGGATGGTGAACTGGAAAGC	59.1	50.0	20
Lu925	1	p2	(GA)13	2	460 462	lu925F	GGGGCAAACAAGTGAAGCTA	60.2	50.0	19+20
						lu925R	GGAGCACAATATGCGAAGGT	60.1	50.0	20
Lu926Ba	2	p2	(AG)14	2	null 670	lu926BF	TCTGAGCACAGATGCATTATCA	59.4	40.9	19+22
Lu926Bb				2	594 596	lu926BR	TTATCAGCTGTGTGGCCTTG	59.9	50.0	20
Lu927	1	p2	(TA)14	3	174 176 180	lu927F	CGGTTTGGCTTTGCTTTTT	60.2	42.1	19+19
						lu927R	TCCAGCAACGGAAATTGAA	60.2	42.1	19
Lu928	1	p2	(CT)15	3	241 243 245	lu928F	TCCCCAAAGTAATATTGAGCAG	58.3	40.9	19+22
						lu928R	CTGGGCCCTACGTGATGTAT	59.8	55.0	20
Lu932	1	p2	(AT)20	4	306 308 312 328	lu932F	CTGGGATGTTAGGAGGTGGA	59.9	55.0	19+20
						lU932R	CCAAACCGATGATCCATACC	60.0	50.0	20
Lu933	1	p2	(AG)20	4	242 250 252 254	lu933F	TCGAACGAGGATGAATGACA	60.2	45.0	19+20
						lu933R	GAAGCTGAAGCTGGGTAACG	60.0	55.0	20
Lu934	1	p2	(TC)20	3	213 215 217	lu934F	GCTTCAACACCAGTCACCAA	59.7	50.0	19+20
						lu934R	CCGGTCCAAAGCTTGAAGA	61.3	52.6	19
Lu935	1	p2	(AT)20	4	346 354 358 360	lu935F	CCAGCAATCATCAGCAGCTA	60.1	50.0	19+20
						lu935R	CAACCTCTATTTATTAATTTAGCAAC	54.5	26.9	26
Lu940	1	p3	(AGG)6	2	null 389	lu940F	ATGGAGGTGCAGAGGATCAC	60.1	55.0	19+20
						lu940R	TAACTCCCTCCTCCCATCCT	59.9	55.0	20
Lu943	1	p3	(TAT)6	4	null 271 274 273	lu943F	AAATAACCACCAAAGCTTGA	55.0	35.0	19+20
						lu943R	AAATCGTGCGATCCGAATAC	59.9	45.0	20
Lu944	1	p3	(CTT)6	3	306 309 312	lu944F	ACGGGTGGCTATGACCAATA	60.2	50.0	19+20
						lu944R	ATCACAGTATGCGGGAAAGG	60.0	50.0	20
Lu951	1	p3	(GCT)6	2	null 288	lu951F	CATGGTATCAGTGGGGCTCT	60.0	55.0	19+20
						lu951R	ATGGAAGAGGAGAAGCAGCA	60.1	50.0	20
Lu955	1	p3	(TTC)6	2	381 445	lu955F	AGATGGACCAGACCGAAGTG	60.1	55.0	19+20
						lu955R	ACCGGAAACGATTTTACCAA	59.3	40.0	20
Lu958	1	p3	(AGT)6	2	386 389	lu958F	AACGGAGCTGCACTGATTCT	60.0	50.0	19+20
						lu958R	CCGGTTAGTTCCTTCCTTCC	59.9	55.0	20
Lu959	1	p3	(GCT)6	2	236 239	lu959F	AAATCGCTGGTCTCTGCTGT	60.0	50.0	19+20
						lu959R	CAACCGGGGAGGGAATAAT	60.9	52.6	19

Lu961B	1	p3	(TTC)6	2	752	755		lu961BF	AATTTTGAAACCCTCGTGGA	59.4	40.0	19+20
								lu961BR	GTCAATCATCCCTTGCCACT	59.9	50.0	20
Lu963	1	p3	(GAT)6	2	369	372		lu963F	CAAAACCTGCTCCTCCCATA	60.1	50.0	19+20
								lu963R	CCACAACCAAATGGGAAATC	60.0	45.0	20
Lu965	1	p3	(CCG)6	2	573	576		lu965F	CATCGGTCTGGCAATTCTTT	60.1	45.0	19+20
								lu965R	GTGCAATGGGAACTCCTGAT	59.9	50.0	20
Lu966	1	p3	(ACA)6	2	377	383		lu966F	CCAAACCTCTGATGGGAAAA	59.9	45.0	19+20
								lu966R	GTTATCAGGTGGGCAGGGTA	59.8	55.0	20
Lu968	1	p3	(CTT)6	2	303	306		lu968F	CCATCCCTTCCTTCTCATCA	60.0	50.0	19+20
								lu968R	GGTGGTGTTAGGTCCCTTCA	59.8	55.0	20
Lu970	1	p3	(TCT)6	2	null	253		lu970F	AATCAGCGGGGAAAGAAGAC	60.6	50.0	19+20
								lu970R	TCTCCCCTCCAACATTTCTG	60.0	50.0	20
Lu980	1	p3	(CGC)6	3	257	263	266	lu980F	CCACACGACACAACACAACA	60.1	50.0	19+20
								lu980R	GCAAGGAGATTATCCGGTGA	60.0	50.0	20
Lu981	1	p3	(TTC)6	2	829	835		lu981F	AACTCCTGCACCAAAGCCTA	59.9	50.0	19+20
								lu981R	GGAGGCAAAAACCAATTGAA	59.9	40.0	20
Lu987	1	p3	(AAG)6	3	285	288	294	lu987F	AAAATTGGAGTGACCGATGC	59.9	45.0	19+20
								lu987R	GAGGAAAGGAGCAAATGCAG	60.0	50.0	20
Lu988	1	p3	(CAC)6	2	null	251		lu988F	ACTCTCACCATGTGCCCTTC	60.1	55.0	19+20
								lu988R	GGTGGGTTGAGATGAATGCT	59.9	50.0	20
Lu989	1	p3	(TGA)6	2	331	334		lu989F	ACGTCGGCTCTGTTGACTCT	60.1	55.0	19+20
								lu989R	TACGGCCCTTACAACAAACC	59.9	50.0	20
Lu990	1	p3	(CCT)6	2	null	383		lu990F	GTCTCCGTCCTGAAAAGCAG	60.0	55.0	19+20
								lu990R	GATCCTATCACCCCCATCAA	59.6	50.0	20
Lu991	1	p3	(CCG)6	2	340	343		lu991F	TCGATTTAATCCTCGCCAAG	60.2	45.0	19+20
								lu991R	CGACAGAGGTGAAGACGACA	60.0	55.0	20
Lu994	1	p3	(CGG)6	2	287	293		lu994F	GTGGTGGAGTAGGCGGAGTA	60.1	60.0	19+20
								lu994R	TCCCTCAACGTTCCATTTTC	59.9	45.0	20
Lu996	1	p3	(TAT)6	2	222	225		lu996F	CGTCCAAACAAAAGATGCAA	59.7	40.0	19+20
								lu996R	GCCTGCAGTTTAGTCGTTGG	60.8	55.0	20
Lu998	1	p3	(TTA)6	3	null	367	382	lu998F	CGTCCGGATTTTGATTCTTC	59.5	45.0	19+20
								lu998R	GAGAGGGAATCGAGGAGGAG	60.3	60.0	20
Lu999	1	p3	(CGG)6	3	593	596	599	lu999F	CTGCGGTTGTTCGGATAAGT	60.1	50.0	19+20
								lu999R	GCTTTCTCGTTCGTTTGCTC	60.1	50.0	20
Lu1001	1	p3	(ATC)6	2	249	252		lu1001F	TGGGTCCAAAGTACCAACAA	58.9	45.0	19+20
								lu1001R	AGCCGTACCAGATGAGGTTG	60.1	55.0	20
Lu1002B	1	p3	(CTT)6	2	null	239		lu1002BF	TCTTCCTTGGCTCTTCTTCG	59.7	50.0	19+20
								lu1002BR	AGCTGGCCAATCATGTTTCT	59.7	45.0	20
Lu1006	1	p3	(TCA)6	2	778	790		lu1006F	TTCTCTGGCTGTGCTTTCCT	60.1	50.0	19+20
								lu1006R	AACATGGGCAAAGAACAAGG	60.0	45.0	20
Lu1007	1	p3	(GCC)6	2	328	331		lu1007F	TTCTGGCAAAACTCCAAACC	60.1	45.0	19+20
								lu1007R	CATCCAGAAGCAATGCAGAA	59.9	45.0	20

Lu1015	1	p3	(GGA)6	2	264 267	lu1015F	GACTTGTTCCTGGGGATTGA	59.9	50.0	19+20
						lu1015R	AAGCGCCATTGTCAGTTAGC	60.4	50.0	20
Lu1022a	2	p3	(AAG)6	3	335 338 341	lu1022F	AGGACCAAAATCGGGAAAAC	60.2	45.0	19+20
Lu1022b				3	null 326 329	lu1022R	GGGGAGGGAGGTTCTAACAA	60.3	55.0	20
Lu1028	1	p3	(CTG)6	2	378 381	lu1028F	TCGTTCTTCTGCAACAATGC	60.0	45.0	19+20
						lu1028R	CCCAACTTGAATGCAGGACT	60.1	50.0	20
Lu1034	1	p3	(TCC)6	2	291 306	lu1034F	CAACCCTTTAGCAGGAGCTG	60.0	55.0	19+20
						lu1034R	GTATGACGGGTCGGATTACG	60.2	55.0	20
Lu1037	1	p3	(TGC)6	2	540 546	lu1037F	TCCCATGAATTGTTGCAGAA	60.0	40.0	19+20
						lu1037R	CAATAGCTGCCCTTGTCCAT	60.1	50.0	20
Lu1039	1	p3	(GCA)6	2	352 355	lu1039F	TGATCCCATGTTCTGGAATG	59.3	45.0	19+20
						lu1039R	AAGTACTTCTGGGCGTCGAA	59.9	50.0	20
Lu1041	1	p3	(TCT)6	3	127 151 154	lu1041F	TTGCGACTGTTCTTGTTGTTG	59.9	42.9	19+21
						lu1041R	CATCTCATTTTGGTTTTGCAG	58.3	38.1	21
Lu1042	1	p3	(AGC)6	2	231 234	lu1042F	TCCGGTGGGAGATCAGTTAG	60.1	55.0	19+20
						lu1042R	GGAAAAGCTCCAGTGTTCCA	60.2	50.0	20
Lu1043	1	p3	(CAT)8	2	385 391	lu1043F	TGCAGGGTTATTCTGGGAAC	59.9	50.0	19+20
						lu1043R	CTGGCAAGTGATCTGCTCAA	60.1	50.0	20
Lu1044B	1	p3	(TTC)6	2	null 486	lu1044BF	CTGTGAAAAAGGCTGCGACT	60.6	50.0	19+20
						lu1044BR	TCCCCCTACTTCAGGTCAAA	59.5	50.0	20
Lu1049	1	p3	(AAT)6	3	null 300 388	lu1049F	AAGGAGTACTGGACGATAATGGA	59.0	43.5	19+23
						lu1049R	ATCCCGACGTTTGTTCCAC	60.8	52.6	19
Lu1050	1	p3	(AAT)6	2	null 229	lu1050F	CCCAGCAGTCGTCTCTCTTC	60.1	60.0	19+20
						lu1050R	AGGAAATCCCTCGTCAACCT	59.9	50.0	20
Lu1052	1	p3	(ATC)6	2	365 371	lu1052F	GCAGTGGAGAGAACCAAAGC	60.0	55.0	19+20
						lu1052R	CACAGGAAAAAGGTGGTGGT	59.9	50.0	20
Lu1055	1	p3	(TTC)6	2	null 450	lu1055F	TTGATGAAGCCGTCAAAATC	58.7	40.0	19+20
						lu1055R	GAGAGGAACGAAGCATGGAG	60.0	55.0	20
Lu1060	1	p3	(TAG)6	2	203 239	lu1060F	GGAGACCCCATTGACACAAC	60.2	55.0	19+20
						lu1060R	GCTCCATTCCTTGCTCTTTG	60.0	50.0	20
Lu1066	1	p3	(CTT)6	3	213 219 222	lu1066F	TTGATCCGGTCATCATCTCA	60.0	45.0	19+20
						lu1066R	AGCTAGGGCGATACGTCTGA	60.0	55.0	20
Lu1071	1	p3	(ATT)7	2	223 226	lu1071F	GGAGCAAGCATTCATGT	52.5	47.0	19+17
						lu1071R	CGTCGTTTCATGTGTTTTGG	60.0	45.0	20
Lu1073	1	p3	(TCT)7	2	873 879	lu1073F	GCACAACCACTACCGGAGAT	60.0	55.0	19+20
						lu1073R	ATCGCGAAAGAAATTGAGGA	59.8	40.0	20
Lu1077	1	p3	(GGT)7	4	329 332 334 335	lu1077F	TCACAGTGCGAGGAATGAAG	60.0	50.0	19+20
						lu1077R	CATAGGGCCTAGCCTCATCA	60.2	55.0	20
Lu1086	1	p3	(ACA)7	2	427 430	lu1086F	CTGACACACCCAATCATTGAA	59.4	42.9	19+21
						lu1086R	ATTTCGGGTTGATGTTGGAG	59.8	45.0	20
Lu1087	1	p3	(ATA)7	2	360 363	lu1087F	AAGTCGGCCAAATCTTTGAT	58.6	40.0	19+20
						lu1087R	GAAGAAGTGGCAGGAGATCG	60.0	55.0	20

Lu1089	1	p3	(TCT)7	2	196	199		lu1089F	TCCACTTCTAACTTCCAACA	53.3	40.0	19+20
								lu1089R	GCGAATGAGCTTTTCGTCTT	59.6	45.0	20
Lu1094	1	p3	(CTC)7	2	357	360		lu1094F	TCAAGGAACAAACCCAATCC	59.8	45.0	19+20
								lu1094R	GCCAATTGTGCCTATGTTGA	59.5	45.0	20
Lu1100	1	p3	(TGC)7	2	261	267		lu1100F	AATCCGGCCAGACAATGTAG	60.0	50.0	19+20
								lu1100R	CTACCACTGCCCCCAAGATA	59.9	55.0	20
Lu1101	1	p3	(GTG)7	2	351	360		lu1101F	TTTCCTTCTCCGTGCGTACT	59.9	50.0	19+20
								lu1101R	GCGTCAGAACTCCAAGAGGA	60.5	55.0	20
Lu1103	1	p3	(TAG)7	2	390	396		lu1103F	GCAACAACTCCACCCTGACT	60.2	55.0	19+20
								lu1103R	CGCAGTTTGAATGTTGAACG	60.3	45.0	20
Lu1104	1	p3	(ATG)7	2	333	339		lu1104F	CAAAACAAAGGGATGGGAAA	59.8	40.0	19+20
								lu1104R	AAGAACAAAGCGCAGAAAGC	59.8	45.0	20
Lu1106	1	p4	(AGTA)5	2	296	300		lu1106F	GGGCAGGTGAAATGACAAGT	60.0	50.0	19+20
								lu1106R	GGAGTGGAGACAGCAAGAGC	60.1	60.0	20
Lu1107	1	p3	(CTT)7	3	283	286	290	lu1107F	ACGGGTGGCTATGACCAATA	60.2	50.0	19+20
								lu1107R	AGACTGCCACGTTCCATTTC	60.1	50.0	20
Lu1112	1	p3	(CAG)7	2	267	270		lu1112F	GCAGCAGTCAATGCATCAGT	60.0	50.0	19+20
								lu1112R	AAGAGGCATCCCCTTGAGAT	60.0	50.0	20
Lu1115	1	p3	(CGG)7	2	null	375		lu1115F	CGGAGCAATTGAAGAAGGAG	59.9	50.0	19+20
								lu1115R	AATAGCAATTCCGTCCGATG	59.9	45.0	20
Lu1116	1	p3	(GAA)7	2	241	244		lu1116F	GATGGAAATAGCAGCGTGGT	60.1	50.0	19+20
								lu1116R	CCGAAGTAGCCATTGTTGGT	60.0	50.0	20
Lu1117	1	p3	(AAG)9	3	200	203	206	lu1117F	GCGGCCATTCTCTATTTCCT	60.6	50.0	19+20
								lu1117R	CTCTTGCTGTTGTTGTTGTTGA	59.0	40.9	22
Lu1119B	1	p3	(TCA)7	2	712	715		lu1119BF	TTCTGAGTTTTTCCCCTCTTTC	58.9	40.9	19+22
								lu1119BR	TAACAACCCTCTCCCGATTG	59.9	50.0	20
Lu1121	1	p3	(TTC)8	2	249	252		lu1121F	GCTATGGCGGAGCTATTGAG	60.0	55.0	19+20
								lu1121R	GCTTTCATTCCCCTGAACAA	60.1	45.0	20
Lu1124	1	p3	(TTC)8	2	305	308		lu1124F	CGATGGTGGATTTGTTTTCC	60.2	45.0	19+20
								lu1124R	GTGGGGAGAAATTGGAAGGT	60.2	50.0	20
Lu1125	1	p3	(ACA)8	2	201	204		lu1125F	CCAAACTGTCCCCACAAATC	60.2	50.0	19+20
								lu1125R	TGCTGAGATGCTGAAAGGAA	59.7	45.0	20
Lu1127	1	p3	(TCT)8	2	523	526		lu1127F	TGGTGCCACAGCTATCAAAG	59.9	50.0	19+20
								lu1127R	GGAGGAGGAGCATAGCACTG	60.0	60.0	20
Lu1130	1	p3	(TAT)8	2	501	507		lu1130F	CAGTTCCATGTGTTTCATTCCA	60.8	40.9	19+22
								lu1130R	ATCCAGAACCAGTTGCAAGG	60.1	50.0	20
Lu1133	1	p3	(GAA)8	2	null	272		lu1133F	TGCCAAGAAAGCAGAAACAA	59.6	40.0	19+20
								lu1133R	GAGTGGAGAGAGGGACGATG	59.8	60.0	20
Lu1134	1	p3	(AAG)8	2	197	200		lu1134F	CGGCTCCCTGAATCCTTAC	59.6	57.9	19+19
								lu1134R	CCAGGTGATGAGCAGCAGTA	60.0	55.0	20
Lu1135	1	p3	(AAT)8	3	287	296	299	lu1135F	CAGTAAGCAATGAAAAGTTCCAGAC	60.5	40.0	19+25
								lu1135R	GAGCCATTGCCTGAGTTCAC	60.8	55.0	20

Lu1136	1	p3	(GCT)8	2	null 260	lu1136F	GTGCAAGAATGGTGGTGATG	60.0	50.0	19+20
						lu1136R	TCCCAACTTCAAACATGACG	59.5	45.0	20
Lu1138	1	p3	(GCT)8	2	332 335	lu1138F	TGGTACAACACGAGCAGCA	60.0	52.6	19+19
						lu1138R	TGGCAGCTATGTGTCTCCAG	60.0	55.0	20
Lu1140a	2	p3	(TGT)8	2	454 457	lu1140F	CACACTTCCCCCATATCCAT	59.5	50.0	19+20
Lu1140b				2	415 444	lu1140R	TCACAAACTGACACACCAATCA	60.0	40.9	22
Lu1144	1	p3	(GAG)8	3	309 312 315	lu1144F	AACTGCAATTAGCCGACGAC	60.3	50.0	19+20
						lu1144R	GCAAACCTCAGCTTTTCTGG	60.0	50.0	20
Lu1145	1	p3	(TCT)8	2	269 275	lu1145F	GGAAATCAACGGTCATGGTT	59.7	45.0	19+20
						lu1145R	GGAGGAGAGGGAGAAATTGG	60.0	55.0	20
Lu1146a	2	p3	(CTG)9	2	307 319	lu1146F	AACCAAATCCGCACTTTGAC	60.0	45.0	19+20
Lu1146b				2	291 303	lu1146R	GAGCAGAGCAGATCAGAATCAG	59.3	50.0	22
Lu1147	1	p3	(AAT)9	2	366 378	lu1147F	AGTGTAGAAGCACCTTGAGATATTATT	57.4	33.3	19+27
						lu1147R	GCGTAGGAGCTGAAACTTGG	60.0	55.0	20
Lu1148	1	p3	(TTC)9	4	267 273 282 288	lu1148F	TGGGACAAAATACGCAGTGA	60.1	45.0	19+20
						lu1148R	TGAGCATTGTGAAGGAGACG	60.0	50.0	20
Lu1151	1	p3	(AAG)9	2	454 466	lu1151F	CGTATGTGGTGATGCCAAAG	60.0	50.0	19+20
						lu1151R	CCGCCTCTTTCAGAATTCAA	60.3	45.0	20
Lu1157	1	p3	(TCT)9	2	389 392	lu1157F	AAGAAACTCGGTGGTGATGG	60.0	50.0	19+20
						lu1157R	AAGAGTATTGCGGGAGAGCA	60.0	50.0	20
Lu1158	1	p3	(CTT)10	2	317 320	lu1158F	ACTTGGACCACATCACAACC	58.2	50.0	19+20
						lu1158R	GTTGGGTACAATGCGGGTAG	60.2	55.0	20
Lu1160	1	p3	(CTT)10	3	381 399 402	lu1160F	CCTACAAACTAAAACGGGACCA	60.3	45.5	19+22
						lu1160R	GGAGAGTGCATCGTCTCACA	60.0	55.0	20
Lu1161	1	p3	(TAT)11	2	216 231	lu1161F	CTTTGATTTGAATAGCGGGTTA	58.3	36.4	19+22
						lu1161R	GCTATTGGCCTCTGCTTCTG	60.1	55.0	20
Lu1162	1	p3	(TTC)11	4	674 677 681 684	lu1162F	GAGTTTTTCCCCCGCAAG	60.6	55.6	19+18
						lu1162R	TGCAAAGGTGATCAAGTCCA	60.2	45.0	20
Lu1163	1	p3	(ATC)11	4	258 264 267 270	lu1163F	GAACACAGTTGGCTCGAACA	59.9	50.0	19+20
						lu1163R	GAAAGCCATCCAGCAAAGAG	60.0	50.0	20
Lu1165	1	p3	(TTA)12	2	304 319	lu1165F	CCTTTCCGCCTTCTCTCTCT	60.1	55.0	19+20
						lu1165R	TACCGGAAGCTACCCATCAC	60.0	55.0	20
Lu1168	1	p3	(TCT)12	3	792 801 804	lu1168F	CACCCCCTTATCGCTCTGTA	60.1	55.0	19+20
						lu1168R	CCATCGCTTCAGGAATGAGT	60.2	50.0	20
Lu1169	1	p3	(CTT)13	2	309 312	lu1169F	CTCCTTGCATGACTCCTTCC	59.8	55.0	19+20
						lu1169R	CTTGGGTGAGAGGCTCTTTG	60.0	55.0	20
Lu1170	1	p3	(CTT)13	2	203 215	lu1170F	GGGAATTGATGGCTACTGGA	59.9	50.0	19+20
						lu1170R	TTACACAGAACGGGAGTGGA	59.1	50.0	20
Lu1171	1	p3	(ATA)13	3	265 268 271	lu1171F	CAAATTTTAAATGAATACAAAACATGA	57.8	18.5	19+27
						lu1171R	GTTGGCCAGAGAGAGATTCG	60.0	55.0	20
Lu1172	1	p3	(TTC)14	2	446 464	lu1172F	TCCATCATTCTTCCAAAGAT	54.6	35.0	19+20
						lu1172R	TGAGGATATGCGAGGAAAGG	60.2	50.0	20

Lu1173	1	p3	(CTT)14	4	176 179 197 200	lu1173F	TGGTCACTTTCCTCCAGTAACTC	59.7	47.8	19+23
						lu1173R	ACCGTTTCTGGTTTGGTTTG	59.9	45.0	20
Lu1174	1	p4	(GAAA)5	2	328 330	lu1174F	TCTCGAACAGGATCCAAACC	60.0	50.0	19+20
						lu1174R	TAGCCAGCATTCCAGGATCT	59.8	50.0	20
Lu1175	1	p4	(ATTA)6	2	277 289	lu1175F	GCAAATGCTGGTGTATATAGATCGT	60.6	40.0	19+25
						lu1175R	TATACCCAAGGTGGCAAAGG	59.8	50.0	20
Lu1176	1	p4	(ACAA)5	2	278 382	lu1176F	TCTGCGATATGAATTGATTGTG	58.7	36.4	19+22
						lu1176R	TTTCATCTTTTGCAGCATGG	59.8	40.0	20
Lu1177	1	p4	(CAAC)5	2	316 320	lu1177F	GTGCGTACCAGATTGCACTG	60.3	55.0	19+20
						lu1177R	CAACGACCAGACTATTCCAACA	60.0	45.5	22
Lu1178	1	p4	(AAGA)5	2	291 299	lu1178F	GCCCTGAATGAGCTACCAGT	59.3	55.0	19+20
						lu1178R	CTTGAATGCAGACGCAGTGT	60.1	50.0	20
Lu1179	1	p4	(TTAA)5	2	405 413	lu1179F	TTTCTTGGCTTTCCTCATCG	60.3	45.0	19+20
						lu1179R	AACTCACCTGGGGGATCAAT	60.6	50.0	20
Lu1182B	1	p4	(GTCT)5	2	211 215	lu1182BF	GATGGTCATGAGTGGCTGAA	59.6	50.0	19+20
						lu1182BR	TACCATGGTGGCTAGGCTGA	61.6	55.0	20
Lu1192	1	p4	(AGCC)5	2	296 300	lu1192F	GCAGCCATCTGGGCACTTA	62.3	57.9	19+19
						lu1192R	CAGCAAATATCCTCCCTCCA	60.0	50.0	20
Total	149			352						
Average				2.36						
