# Supplementary material for: Simple sequence repeat marker development from bacterial artificial chromosome end sequences and expressed sequence tags of flax (Linum usitatissimum L.)
Source: Theor Appl Genet. 2012 Apr 7;125(4):685–94. doi: 10.1007/s00122-012-1860-4 (PMC3405236; doi:10.1007/s00122-012-1860-4)
Supplement: Supplementary file 4 — Supplementary material 4 (PDF 246 kb) [file 122_2012_1860_MOESM4_ESM.pdf]

Supplementary Data Table S2: SSR description based on motifs, source, number of repeats and polymorphism

	No of repeats		No of SSRs		Polymorphic				Monomorphic				DNW			
Motif	BES	EST	BES	EST	No BES	% BES	No EST	% EST	No BES	% BES	No EST	% EST	No BES	% BES	No EST	% EST
**Dinucleotides**																
AT/AT	9 - 22	9 - 20	111	14	92	83	12	86	18	16	1	7	1	1	1	7
TA/TA	9 - 25	9 - 14	96	7	73	76	7	100	21	22	0	0	2	2	0	0
**AT/TA**	**9 - 25**	**9 - 20**	**207**	**21**	**165**	**80**	**19**	**90**	**39**	**19**	**1**	**5**	**3**	**1**	**1**	**5**
AG/CT	9 - 25	9 - 23	82	15	57	70	6	40	23	28	2	13	2	2	7	47
GA/TC	9 - 20	9 - 20	54	15	40	74	7	47	14	26	3	20	0	0	5	33
**AG/GA/CT/TC**	**9 - 25**	**9 - 23**	**136**	**30**	**97**	**71**	**13**	**43**	**37**	**27**	**5**	**17**	**2**	**1**	**12**	**40**
AC/GT	9	-	1	-	1	100	0	-	0	0	0	-	0	0	0	-
CA/TG	9 - 10	9	2	1	2	100	1	100	0	0	0	0	0	0	0	0
**AC/CA/TG/GT**	**9 - 10**	**9**	**3**	**1**	**3**	**100**	**1**	**100**	**0**	**0**	**0**	**0**	**0**	**0**	**0**	**0**
CG/CG	-	-	-	-	0	-	0	-	0	-	0	-	0	-	0	-
GC/GC	-	-	-	-	0	-	0	-	0	-	0	-	0	-	0	-
**CG/GC**	**-**	**-**	**0**	**0**	**0**	**-**	**0**	**-**	**0**	**-**	**0**	**-**	**0**	**-**	**0**	**-**
Di- sub-total			**346**	**52**	**265**	**77**	**33**	**63**	**76**	**22**	**6**	**12**	**5**	**1**	**13**	**25**
**Trinucleotides**																
AAG/CTT	6 - 16	6 - 14	67	30	35	52	15	50	32	48	14	47	0	0	1	3
AGA/TCT	6 - 17	6 - 18	60	23	39	65	8	35	21	35	10	43	0	0	5	22
GAA/TTC	6 - 17	6 - 14	102	38	50	49	12	32	51	50	22	58	1	1	4	11
**AAG/AGA/GAA/CTT/**	**6 - 17**	**6 - 18**	**229**	**91**	**124**	**54**	**35**	**38**	**104**	**45**	**46**	**51**	**1**	**0**	**10**	**11**
AAT/ATT	6 - 14	6 - 9	50	7	32	64	5	71	18	36	2	29	0	0	0	0
ATA/TAT	6 - 16	6 - 13	33	7	24	73	6	86	9	27	1	14	0	0	0	0
TAA/TTA	6 - 18	6 - 12	29	5	14	48	2	40	14	48	1	20	1	3	2	40
**AAT/ATA/TAA/ATT/T**	**6 - 18**	**6 - 13**	**112**	**19**	**70**	**63**	**13**	**68**	**41**	**37**	**4**	**21**	**1**	**1**	**2**	**11**
ATG/CAT	6 - 10	6 - 8	20	10	9	45	2	20	11	55	7	70	0	0	1	10
ATC/GAT	6 - 9	6 - 11	35	13	14	40	4	31	21	60	8	62	0	0	1	8
TCA/TGA	6 - 10	6 - 8	34	6	16	47	3	50	18	53	3	50	0	0	0	0
**ATG/TGA/GAT/CAT/**	**6 - 10**	**6 - 11**	**89**	**29**	**39**	**44**	**9**	**31**	**50**	**56**	**18**	**62**	**0**	**0**	**2**	**7**
AGG/CCT	6 - 7	6 - 7	9	7	4	44	2	29	5	56	5	71	0	0	0	0
GGA/TCC	6 - 10	6 - 8	27	9	13	48	2	22	14	52	7	78	0	0	0	0
CTC/GAG	6 - 9	6 - 9	13	9	7	54	2	22	6	46	4	44	0	0	3	33
**AGG/GGA/GAG/CCT**	**6 - 10**	**6 - 9**	**49**	**25**	**24**	**49**	**6**	**24**	**25**	**51**	**16**	**64**	**0**	**0**	**3**	**12**
AGC/GCT	6 - 10	6 - 8	14	9	4	29	5	56	10	71	4	44	0	0	0	0
GCA/TGC	6 - 8	6 - 8	8	7	2	25	3	43	6	75	4	57	0	0	0	0
CAG/CTG	6 - 13	6 - 9	22	11	12	55	3	27	10	45	6	55	0	0	2	18
**AGC/GCA/CAG/GCT**	**6 - 13**	**6 - 9**	**44**	**27**	**18**	**41**	**11**	**41**	**26**	**59**	**14**	**52**	**0**	**0**	**2**	**7**
AAC/GTT	6 - 10	6 - 7	7	5	4	57	0	0	3	43	5	100	0	0	0	0
ACA/TGT	6 - 8	6 - 9	8	7	4	50	4	57	4	50	3	43	0	0	0	0
CAA/TTG	6 - 13	6 - 7	24	5	10	42	0	0	14	58	5	100	0	0	0	0
**AAC/ACA/CAA/GTT/**	**6 - 13**	**6 - 9**	**39**	**17**	**18**	**46**	**4**	**24**	**21**	**54**	**13**	**76**	**0**	**0**	**0**	**0**

ACC/GGT	6 - 7	7	15	2	5	33	1	50	8	53	1	50	2	13	0	0
CCA/TGG	6	6 - 7	8	3	1	13	0	0	7	88	2	67	0	0	1	33
CAC/GTG	6	6 - 7	3	4	0	0	2	50	3	100	2	50	0	0	0	0
**ACC/CCA/CAC/GGT/**	**6 - 7**	**6 - 7**	**26**	**9**	**6**	**23**	**3**	**33**	**18**	**69**	**5**	**56**	**2**	**8**	**1**	**11**
GTA/TAC	6 - 8	6	6	1	3	50	0	0	3	50	1	100	0	0	0	0
ACT/AGT	6 - 10	6 - 8	9	3	5	56	1	33	3	33	2	67	1	11	0	0
CTA/TAG	6 - 7	6 - 9	5	4	2	40	2	50	3	60	2	50	0	0	0	0
**AGT/GTA/TAG/ACT/(**	**6 - 10**	**6 - 9**	**20**	**8**	**10**	**50**	**3**	**38**	**9**	**45**	**5**	**63**	**1**	**5**	**0**	**0**
GCC/GGC	6 - 7	6	3	1	0	0	1	100	3	100	0	0	0	0	0	0
CCG/CGG	6 - 8	6 - 7	9	7	3	33	5	71	6	67	1	14	0	0	1	14
CGC/GCG	6 - 8	6	3	1	3	100	1	100	0	0	0	0	0	0	0	0
**GGC/GCG/CGG/GCC**	**6 - 8**	**6 - 7**	**15**	**9**	**6**	**40**	**7**	**78**	**9**	**60**	**1**	**11**	**0**	**0**	**1**	**11**
ACG/CGT	-	-	-	-	0	-	0	-	0	-	0	-	0	-	0	-
CGA/TCG	6 - 7	-	8	-	2	25	0	-	6	75	0	-	0	0	0	-
GAC/GTC	6 - 7	8	5	1	2	40	0	0	3	60	1	100	0	0	0	0
**ACG/CGA/GAC/CGT**	**6 - 7**	**8**	**13**	**1**	**4**	**31**	**0**	**0**	**9**	**69**	**1**	**100**	**0**	**0**	**0**	**0**
Tri- sub-total			**636**	**235**	**319**	**50**	**91**	**39**	**312**	**49**	**123**	**52**	**5**	**1**	**21**	**9**
**Tetranucleotides**																
AAAG/CTTT	5 - 10	5 - 7	10	3	5	50	0	0	4	40	3	100	1	10	0	0
AAGA/TCTT	5 - 8	5	3	2	1	33	1	50	2	67	1	50	0	0	0	0
AGAA/TTCT	5 - 9	5	3	1	3	100	0	0	0	0	1	100	0	0	0	0
GAAA/TTTC	5 - 9	5 - 6	4	3	1	25	1	33	3	75	2	67	0	0	0	0
**AAAG/AAGA/AGAA/(**	**5 - 10**	**5 - 7**	**20**	**9**	**10**	**50**	**2**	**22**	**9**	**45**	**7**	**78**	**1**	**5**	**0**	**0**
AATT/AATT	5 - 6	-	4	0	2	50	0	-	2	50	0	-	0	0	0	-
ATTA/TAAT	5 - 7	6	8	1	3	38	1	100	5	63	0	0	0	0	0	0
TTAA/TTAA	5	5	1	1	0	0	1	100	1	100	0	0	0	0	0	0
**AATT/ATTA/TTAA/T/**	**5 - 7**	**5 - 6**	**13**	**2**	**5**	**38**	**2**	**100**	**8**	**62**	**0**	**0**	**0**	**0**	**0**	**0**
AAAC/GTTT	5 - 6	-	2	0	0	0	0	-	1	50	0	-	1	50	0	-
AACA/TGTT	5	-	3	0	1	33	0	-	2	67	0	-	0	0	0	-
ACAA/TTGT	5 - 6	5	2	2	1	50	1	50	1	50	1	50	0	0	0	0
CAAA/TTTG	5	-	3	0	0	0	0	-	3	100	0	-	0	0	0	-
**AAAC/AACA/ACAA/(**	**5 - 6**	**5**	**10**	**2**	**2**	**20**	**1**	**50**	**7**	**70**	**1**	**50**	**1**	**10**	**0**	**0**
AAAT/ATTT	5	-	3	0	0	0	0	-	3	100	0	-	0	0	0	-
AATA/TATT	5 - 7	-	3	0	2	67	0	-	1	33	0	-	0	0	0	-
TAAA/TTTA	5 - 6	-	4	0	1	25	0	-	3	75	0	-	0	0	0	-
**AAAT/AATA/ATAA/T**	**5 - 7**	**-**	**10**	**0**	**3**	**30**	**0**	**-**	**7**	**70**	**0**	**-**	**0**	**0**	**0**	**-**
ATAG/CTAT	5	-	1	0	1	100	0	-	0	0	0	-	0	0	0	-
TAGA/TCTA	6	-	1	0	0	0	0	-	1	100	0	-	0	0	0	-
ATCT/AGAT	5	-	2	0	1	50	0	-	1	50	0	-	0	0	0	-
GATA/TATC	5 - 7	-	3	0	1	33	0	-	2	67	0	-	0	0	0	-
**ATAG/TAGA/AGAT/(**	**5 - 7**	**-**	**7**	**0**	**3**	**43**	**0**	**-**	**4**	**57**	**0**	**-**	**0**	**0**	**0**	**-**
AAGG/CCTT	5	5	1	1	0	0	0	0	1	100	1	100	0	0	0	0
AGGA/TCCT	6	-	1	0	1	100	0	-	0	0	0	-	0	0	0	-

GGAA/TTCC	5 - 6	-	2	0	2	100	0	-	0	0	0	-	0	0	0	-
CTTC/GAAG	5	-	2	0	2	100	0	-	0	0	0	-	0	0	0	-
**AAGG/AGGA/GGAA/**	**5 - 6**	**5**	**6**	**1**	**5**	**83**	**0**	**0**	**1**	**17**	**1**	**100**	**0**	**0**	**0**	**0**
AGCT/AGCT	5	-	1	0	1	100	0	-	0	0	0	-	0	0	0	-
GCTA/TAGC	5	-	2	0	0	0	0	-	1	50	0	-	1	50	0	-
CTAG/CTAG	5	-	3	0	2	67	0	-	1	33	0	-	0	0	0	-
**AGTC/GCTA/CTAG/A**	**5**	**-**	**6**	**0**	**3**	**50**	**0**	**-**	**2**	**33**	**0**	**-**	**1**	**17**	**0**	**-**
ATCC/GGAT	5	5	1	1	0	0	0	0	1	100	1	100	0	0	0	0
ATGG/CCAT	8	-	1	0	1	100	0	-	0	0	0	-	0	0	0	-
CATC/GATG	5	-	3	0	0	0	0	-	3	100	0	-	0	0	0	-
**ATCC/TCCA/CCAT/C**	**5 - 8**	**5**	**5**	**1**	**1**	**20**	**0**	**0**	**4**	**80**	**1**	**100**	**0**	**0**	**0**	**0**
AACC/GGTT	5 - 10	-	3	0	3	100	0	-	0	0	0	-	0	0	0	-
ACCA/TGGT	5	-	1	0	0	0	0	-	1	100	0	-	0	0	0	-
CAAC/GTTG	5	5	1	1	1	100	1	100	0	0	0	0	0	0	0	0
**AACC/ACCA/CCAA/C**	**5 - 10**	**5**	**5**	**1**	**4**	**80**	**1**	**100**	**1**	**20**	**0**	**0**	**0**	**0**	**0**	**0**
ACAT/ATGT	5 - 6	-	2	0	1	50	0	-	1	50	0	-	0	0	0	-
ATAC/GTAT	5 - 6	-	2	0	0	0	0	-	2	100	0	-	0	0	0	-
TACA/TGTA	5	-	1	0	0	0	0	-	1	100	0	-	0	0	0	-
**ACAT/CATA/ATAC/T**	**5 - 6**	**-**	**5**	**0**	**1**	**20**	**0**	**-**	**4**	**80**	**0**	**-**	**0**	**0**	**0**	**-**
ACTG/CAGT	5	-	1	0	1	100	0	-	0	0	0	-	0	0	0	-
GTCA/TGAC	5	-	2	0	1	50	0	-	1	50	0	-	0	0	0	-
AGTC/GACT	5	-	1	0	0	0	0	-	1	100	0	-	0	0	0	-
**ACTG/CTGA/TGAC/C**	**5**	**-**	**4**	**0**	**2**	**50**	**0**	**-**	**2**	**50**	**0**	**-**	**0**	**0**	**0**	**-**
ACTC/GAGT	5	-	3	0	2	67	0	-	1	33	0	-	0	0	0	-
GTGA/TCAC	6	-	1	0	0	0	0	-	1	100	0	-	0	0	0	-
**ACTC/CTCA/TCAC/C**	**6**	**-**	**4**	**0**	**2**	**50**	**0**	**-**	**2**	**50**	**0**	**-**	**0**	**0**	**0**	**-**
AGCC/GGCT	-	5	0	1	0	-	1	100	0	-	0	0	0	-	0	0
GCCA/TGGC	5	-	1	0	0	0	0	-	1	100	0	-	0	0	0	-
CAGC/GCTG	5	5	1	1	0	0	0	0	1	100	1	100	0	0	0	0
**AGCC/GCCA/CCAG/**	**5**	**5**	**2**	**2**	**0**	**0**	**1**	**50**	**2**	**100**	**1**	**50**	**0**	**0**	**0**	**0**
AATG/CATT	5	-	1	0	0	0	0	-	1	100	0	-	0	0	0	-
ATCA/TGAT	5	-	1	0	0	0	0	-	1	100	0	-	0	0	0	-
TGAA/TTCA	5	-	1	0	0	0	0	-	1	100	0	-	0	0	0	-
**AATG/ATGA/TGAA/C**	**5**	**-**	**3**	**0**	**0**	**0**	**0**	**-**	**3**	**100**	**0**	**-**	**0**	**0**	**0**	**-**
CGTA/TACG	5	-	2	0	0	0	0	-	2	100	0	-	0	0	0	-
GTAC/GTAC	5	-	1	0	1	100	0	-	0	0	0	-	0	0	0	-
**CGTA/GTAC/TACG/A**	**5**	**-**	**3**	**0**	**1**	**33**	**0**	**-**	**2**	**67**	**0**	**-**	**0**	**0**	**0**	**-**
CTCC/GGAG	6	-	1	0	1	100	0	-	0	0	0	-	0	0	0	-
CCTC/GAGG	5	6	1	1	0	0	0	0	1	100	0	0	0	0	1	100
**AGGG/GGGA/GGAG**	**5 - 6**	**6**	**2**	**1**	**1**	**50**	**0**	**0**	**1**	**50**	**0**	**0**	**0**	**0**	**1**	**100**
AGGT/ACCT	6	-	1	0	0	0	0	-	1	100	0	-	0	0	0	-
CTAC/GTAG	6	-	1	0	0	0	0	-	1	100	0	-	0	0	0	-

**AGGT/GGTA/GTAG/**	**6**	**-**	**2**	**0**	**0**	**0**	**0**	**-**	**2**	**100**	**0**	**-**	**0**	**0**	**0**	**-**
GTTA/TAAC	6	-	2	0	1	50	0	-	1	50	0	-	0	0	0	-
**GTTA/TTAG/TAGT/A**	**6**	**-**	**2**	**0**	**1**	**50**	**0**	**-**	**1**	**50**	**0**	**-**	**0**	**0**	**0**	**-**
AGCA/TGCT	5	-	1	0	0	0	0	-	1	100	0	-	0	0	0	-
**AGCA/GCAA/CAAG/**	**5**	**-**	**1**	**0**	**0**	**0**	**0**	**-**	**1**	**100**	**0**	**-**	**0**	**0**	**0**	**-**
ATGC/GCAT	5	-	1	0	0	0	0	-	1	100	0	-	0	0	0	-
**ATGC/TGCA/GCAT/(**	**5**	**-**	**1**	**0**	**0**	**0**	**0**	**-**	**1**	**100**	**0**	**-**	**0**	**0**	**0**	**-**
CGAG/CTCG	5	-	1	0	0	0	0	-	1	100	0	-	0	0	0	-
**CGAG/GAGC/AGCG/**	**5**	**-**	**1**	**0**	**0**	**0**	**0**	**-**	**1**	**100**	**0**	**-**	**0**	**0**	**0**	**-**
CGGA/TCCG	5	-	1	0	0	0	0	-	1	100	0	-	0	0	0	-
**CGGA/GGAC/GACG/**	**5**	**-**	**1**	**0**	**0**	**0**	**0**	**-**	**1**	**100**	**0**	**-**	**0**	**0**	**0**	**-**
TCAA/TTGA	5	-	1	0	0	0	0	-	1	100	0	-	0	0	0	-
**TCAA/CAAT/AATC/A**	**5**	**-**	**1**	**0**	**0**	**0**	**0**	**-**	**1**	**100**	**0**	**-**	**0**	**0**	**0**	**-**
AGAC/GTCT	-	5	0	1	0	-	1	100	0	-	0	0	0	-	0	0
**AGAC/GACA/ACAG/(**	**-**	**5**	**0**	**1**	**0**	**-**	**1**	**100**	**0**	**-**	**0**	**0**	**0**	**-**	**0**	**0**
AGTA/TACT	-	5	0	1	0	-	1	100	0	-	0	0	0	-	0	0
**AGTA/GTAA/TAAG/A**	**-**	**5**	**0**	**1**	**0**	**-**	**1**	**100**	**0**	**-**	**0**	**0**	**0**	**-**	**0**	**0**
Tetra- sub-total			**114**	**21**	**44**	**39**	**9**	**43**	**67**	**59**	**11**	**52**	**3**	**3**	**1**	**5**
**Pentanucleotides**																
AAAAG/CTTTT	5	-	1	-	0	0	0	-	1	100	0	-	0	0	0	-
AACCC/GGGTT	5	-	1	-	1	100	0	-	0	0	0	-	0	0	0	-
AAGAG/CTCTT	7	-	1	-	1	100	0	-	0	0	0	-	0	0	0	-
AATAG/CTATT	5	-	1	-	1	100	0	-	0	0	0	-	0	0	0	-
AGAAC/GTTCT	5	-	1	-	0	0	0	-	1	100	0	-	0	0	0	-
AGAAG/CTTCT	5	-	1	-	1	100	0	-	0	0	0	-	0	0	0	-
AGAGG/CCTCT	5	-	1	-	0	0	0	-	1	100	0	-	0	0	0	-
AGGAT/ATCCT	5	-	1	-	0	0	0	-	1	100	0	-	0	0	0	-
ATATA/TATAT	5	-	1	-	1	100	0	-	0	0	0	-	0	0	0	-
ATTAA/TTAAT	5	-	1	-	1	100	0	-	0	0	0	-	0	0	0	-
ATTAT/ATAAT	7	-	1	-	0	0	0	-	1	100	0	-	0	0	0	-
CAAAA/TTTTG	5	-	1	-	0	0	0	-	1	100	0	-	0	0	0	-
CATCA/TGATG	5	-	1	-	1	100	0	-	0	0	0	-	0	0	0	-
CATGA/TCATG	5	-	1	-	1	100	0	-	0	0	0	-	0	0	0	-
CCATA/TATGG	5	-	1	-	0	0	0	-	1	100	0	-	0	0	0	-
CGTAG/CTACG	6	-	1	-	1	100	0	-	0	0	0	-	0	0	0	-
GAACA/TGTTC	5	-	1	-	1	100	0	-	0	0	0	-	0	0	0	-
GAAGA/TCTTC	7	5	1	1	1	100	0	0	0	0	1	100	0	0	0	0
GAGAC/GTCTC	6	-	1	-	1	100	0	-	0	0	0	-	0	0	0	-
GATTA/TAATC	5	-	1	-	1	100	0	-	0	0	0	-	0	0	0	-
GTTCA/TGAAC	5	-	1	-	0	0	0	-	1	100	0	-	0	0	0	-
Penta- sub-total			**21**	**1**	**13**	**62**	**0**	**0**	**8**	**38**	**1**	**100**	**0**	**0**	**0**	**0**
**Hexanucleotides**																

AGCCCA/TGGGCT	5	-	1	-	1	100	0	-	0	0	0	-	0	0	0	-
ATTAGG/CCTAAT	9	-	1	-	1	100	0	-	0	0	0	-	0	0	0	-
CAAAAT/ATTTTG	5	-	1	-	1	100	0	-	0	0	0	-	0	0	0	-
CAAAAA/TTTTTG	5	-	1	-	1	100	0	-	0	0	0	-	0	0	0	-
CCAAGA/TCTTGG	5	-	1	-	1	100	0	-	0	0	0	-	0	0	0	-
CTCATC/GATGAG	5	-	1	-	1	100	0	-	0	0	0	-	0	0	0	-
CTGCGA/TCGCAG	5	-	1	-	1	100	0	-	0	0	0	-	0	0	0	-
GGCCCA/TGGGCC	5	-	1	-	1	100	0	-	0	0	0	-	0	0	0	-
GTCCAA/TTGGAC	5	-	1	-	0	0	0	-	1	100	0	-	0	0	0	-
TAATGA/TCATTA	6	-	1	-	1	100	0	-	0	0	0	-	0	0	0	-
TCCCAA/TTGGGA	7	-	1	-	1	100	0	-	0	0	0	-	0	0	0	-
Hexa- sub-total			**11**	**-**	**10**	**91**	**0**	**-**	**1**	**9**	**0**	**-**	**0**	**0**	**0**	**-**
**Compound**																
all motifs			**36**	**33**	**22**	**61**	**12**	**36**	**14**	**39**	**12**	**36**	**0**	**0**	**9**	**27**
Compound sub-total			**36**	**33**	**22**	**61**	**12**	**36**	**14**	**39**	**12**	**36**	**0**	**0**	**9**	**27**
**Grand total**			**1164**	**342**	**673**	**58**	**145**	**42**	**478**	**41**	**153**	**45**	**13**	**1**	**44**	**13**
